# A unifying equation for fermentation sustainability across the titer-rate-yield landscape

**DOI:** 10.1038/s41467-026-75285-1

**Published:** 2026-07-10

**Authors:** Sarang S. Bhagwat, Christopher V. Rao, Huimin Zhao, Vijay Singh, Jeremy S. Guest

**Affiliations:** 1 DOE Center for Advanced Bioenergy and Bioproducts Innovation (CABBI), University of Illinois Urbana-Champaign, Urbana, IL USA; 2 The Grainger College of Engineering, Department of Civil and Environmental Engineering, University of Illinois Urbana-Champaign, Urbana, IL USA; 3 Department of Chemical and Biomolecular Engineering, University of Illinois Urbana-Champaign, Urbana, IL USA; 4 Department of Agricultural and Biological Engineering, University of Illinois Urbana-Champaign, Urbana, IL USA; 5 Institute for Sustainability, Energy, and Environment (iSEE), University of Illinois Urbana-Champaign, Urbana, IL USA

**Keywords:** Chemical engineering, Industrial microbiology, Applied mathematics

## Abstract

Industrial fermentation is central to the sustainable production of fuels and chemicals, yet commercial viability of emerging technologies hinges on improving fermentation titer, rate, and yield (TRY). How these metrics shape system cost remains difficult to generalize due to complex interactions among feedstocks, fermentation, separations, catalytic upgrading, waste management, and facility design. Here, we systematically map theoretical fermentation performance spaces (formed by all potential TRY combinations) for 32 representative biomanufacturing facilities—spanning distinct choices for feedstocks, fermentation regimes and products, separations, and catalytic upgrading—by simulating and evaluating them (via techno-economic analysis, TEA) under uncertainty (600,000 Monte Carlo simulations) and across TRY combinations (7500 TRY combinations for each of 32 configurations). Across this wide design and thermodynamic simulation space, we find the relationship between fermentation TRY and system cost is captured by a simple, generalizable mathematical equation (R^2^ of 0.992 − 1.000 across our simulations; 0.954 − 1.000 when validated against prior studies that used different tools). We use this equation to elucidate key drivers that shape cost sensitivity to fermentation performance, generating widely applicable insights. By demonstrating a unifying relationship governs the impact of fermentation on biomanufacturing economics, this work establishes a foundation for agile, holistically predictive, resource-efficient strategies to prioritize fermentation research and development needs and accelerate commercialization of emerging biomanufacturing technologies.

## Abstract (web summary)

Industrial fermentation’s success hinges on improving titer, rate, and yield (TRY), but how TRY relates to system cost is unknown. Here, the authors map the performance of 32 biomanufacturing facilities and find a generalizable mathematical equation relating TRY and system cost.

## Introduction

Microbial conversions have been used to manufacture products of commercial interest and societal benefit, including pharmaceuticals, nutraceuticals, and other speciality chemicals, as well as fuel ethanol^1^. Industrial fermentation—the use of naturally occurring or genetically engineered microorganisms to produce targeted chemicals—has been identified as a promising family of technologies to convert substrates obtained from renewable feedstocks (e.g., terrestrial crops, agricultural residue, and food waste) into fuels and commodity chemicals^1–4^. A wide range of industrial fermentation processes is being actively developed by the scientific community as promising strategies to produce at least 7 of the “top 10 value-added chemicals” and as components in at least 2 of the 9 potential sustainable aviation fuel production pathways identified by the United States Department of Energy (US DOE)^5,6^.

Rapid ongoing advancements in the biological production of fuels and commodity chemicals are enabled by the metabolic engineering of microorganisms, including yeasts, bacteria, and fungi^1,7^. For emerging fermentation technologies to be successfully commercialized, it will be critical to prioritize fermentation advancements that improve the financial viability and environmental benefits of biorefineries (biomanufacturing facilities) that could leverage these technologies^1,8,9^. Several previous studies have stressed the importance of fermentation yield, titer, and rate (or productivity) to system cost^1,7,9–19^. By employing techno-economic analysis (TEA) and life cycle assessment (LCA) under uncertainty, fermentation yield, titer, and productivity have been identified as key fermentation performance metrics driving biomanufacturing facility financial viability and environmental benefits^10–18^. Although it is difficult to perform rigorous uncertainty and sensitivity analyses using commercial TEA-LCA tools such as Aspen Plus or SuperPro (we are aware of only two studies^19,20^ that evaluated system cost or environmental impacts over multiple yield-titer-productivity combinations using these tools), agile computational frameworks such as the open-source BioSTEAM^21^ platform can enable users to design, simulate, and evaluate biomanufacturing facilities over entire theoretical fermentation spaces (formed by all potential combinations of yield, titer, and productivity values) to set quantitative fermentation performance targets^10–18^. Uncertainty analyses and global sensitivity analyses can greatly help reveal the influence of biomanufacturing facility design decisions, technological performance, and contextual parameters on system cost and environmental impacts.

Despite the growing use of uncertainty and sensitivity analyses in TEA-LCA of emerging fermentation technologies, it is not fundamentally clear how microbial fermentation interacts with the rest of the biomanufacturing facility to influence system cost and environmental impacts. Although quantitative targets for fermentation yield and titer have been identified for individual feedstock-to-product pathways, these targets change substantially under alternative assumptions. Upstream feedstock choices, the design of downstream separation and catalytic upgrading processes, the biomanufacturing facility’s wastewater resource recovery (WWRR) and combined heat and power (CHP) generation facilities, and the uncertainties in the technological and contextual parameters associated with each of these processes together give rise to complex interactions that alter how fermentation performance influences system sustainability^10,12–14,16,18^, limiting the utility of TEA-LCA to prioritize early-stage fermentation research needs. There is a need for generalizable insights into how fermentation yield, titer, and productivity influence system cost and environmental impacts under uncertainty for any feedstock-to-product pathway.

In this work, we provide generalizable mathematical insights into how microbial fermentation interacts with the rest of the biomanufacturing facility to influence system cost under uncertainty. We perform 240,000 simulations across theoretical fermentation spaces for 32 biomanufacturing facilities with mutually distinct combinations of feedstock choice, fermentation regime, fermentation product, separations, and catalytic upgrading. We propose a generalized mathematical equation to robustly capture the relationships between fermentation performance and the minimum product selling price (MPSP, $∙kg^−1^) from TEA. We validate the proposed mathematical equation using external TEA results from studies that used other, commercial tools such as Aspen Plus and SuperPro. Further, we perform 600,000 Monte Carlo simulations across theoretical fermentation spaces for 24 biomanufacturing facilities. By combining uncertainty analyses and global sensitivity analyses with the proposed mathematical equation, we elucidate how biomanufacturing facility configuration and uncertainties in biomanufacturing facility technological and contextual parameters not only influence the system cost, but also influence the relationships between system cost and fermentation performance. Finally, we use the developed equation to analytically compute local sensitivities (as partial derivatives) of system cost with respect to fermentation yield and titer for 32 biomanufacturing facilities and reveal the relative influence of these two parameters on system cost to prioritize decision-making in fermentation research, development, and deployment.

## Results

### Generalized trends in how fermentation performance influences system cost

**Fig. 1:**
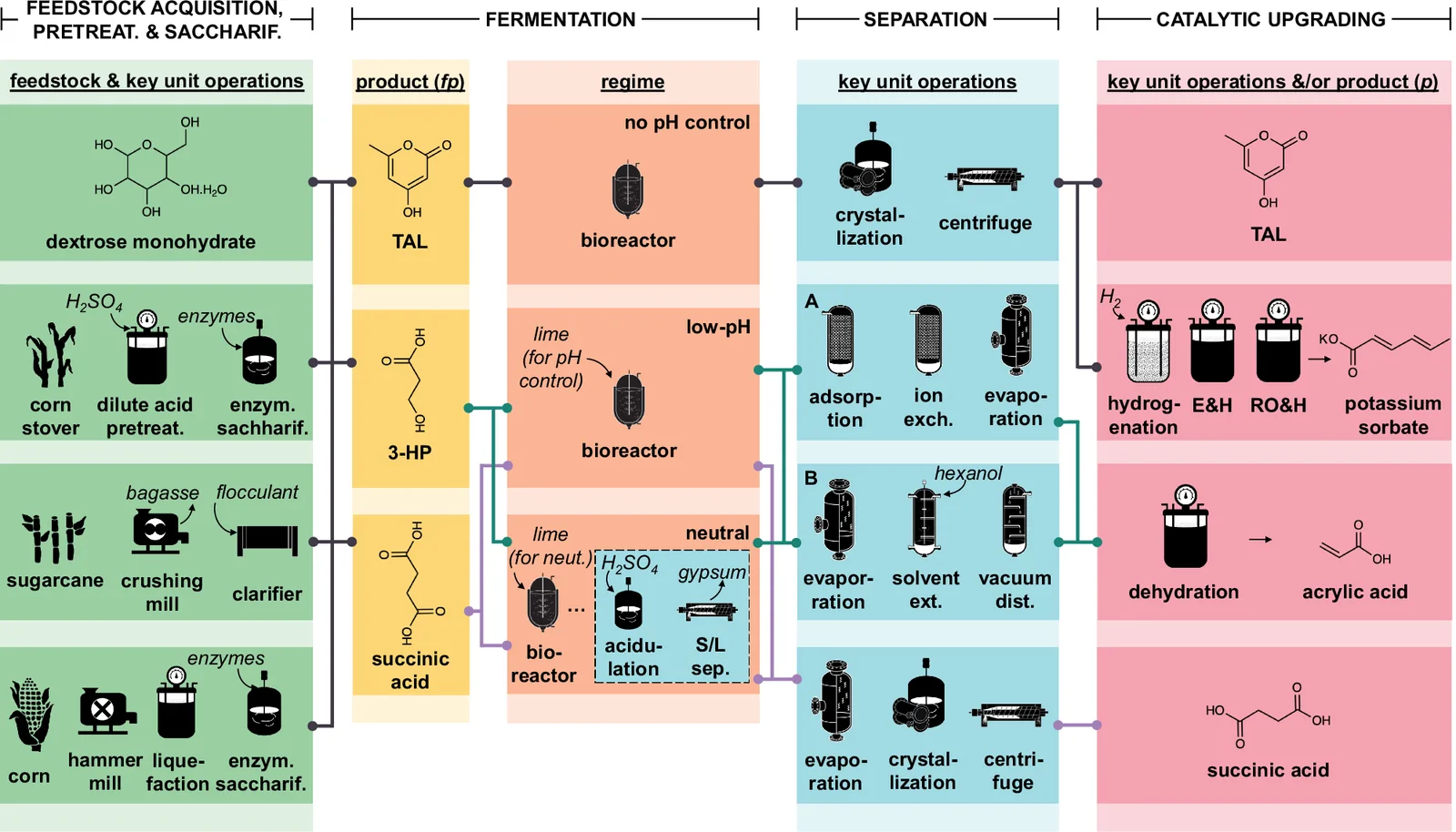
Biomanufacturing facility configurations evaluated in this study. Overview of the 32 biomanufacturing facility configurations evaluated in this study, formed by combinations of discrete design decisions. Abbreviations denote pretreatment (*pretreat*.), saccharification (*saccharif*.), enzymatic (*enzym*.), triacetic acid lactone (*TAL*), 3-hydroxypropionic acid (*3HP*), solid-liquid separation (*S/L sep*.), neutralization (*neut*.), ion exchange (*ion exch*.), solvent extraction (*solvent ext*.), vacuum distillation (*vacuum dist*.), etherification & hydrolysis (*E&H*), and ring-opening & hydrolysis (*RO&H*). Fermentation product (*fp*) choices limited downstream alternatives, as indicated by the colors of circular-headed lines (teal for 3HP, purple for succinic acid); e.g., 3HP biomanufacturing facilities could only be configured with separation process A (*A*) or B (*B*). All separation alternatives included centrifugation of cell mass (not depicted) upstream to the key unit operations listed. Under neutral fermentation regimes, separation alternatives additionally included acidulation and gypsum removal immediately downstream of centrifugation of cell mass. For catalytic upgrading alternatives with no unit operations depicted, the fermentation product (*fp*) is the same as the main biomanufacturing facility product (*p*; i.e., for which minimum product selling prices are estimated). In addition to the depicted processes, each configuration included: wastewater treatment; facilities for heating, cooling, and power utility generation; a heat exchanger network optimized to each simulation; a process water center (for water reuse); and other auxiliary units for storage, air distribution, and clean-in-place. Models for all process alternatives and facilities were from previous studies. Further details on all processes and facilities are available in the Methods under System description. All listed models are available open-source in the online repositories^36–39^.

To consider the influence of biomanufacturing facility design decisions, we chose 32 biomanufacturing facilities designed from combinations of four feedstocks (dextrose monohydrate, sugarcane, corn stover, and corn), three fermentation products (triacetic acid lactone, TAL; 3-hydroxypropionic acid, 3HP; and succinic acid), four separation processes (separation process A and B for 3HP biomanufacturing facilities, and one choice each for TAL and succinic acid biomanufacturing facilities), two fermentation regimes (neutral and low-pH for 3HP and succinic acid biomanufacturing facilities), and two catalytic upgrading processes (3HP was upgraded to acrylic acid for all 3HP biomanufacturing facilities; TAL biomanufacturing facilities either sold TAL as the main product or catalytically upgraded it to potassium sorbate). All biomanufacturing facility configurations are shown in Fig. 1 and listed in Supplementary Data 1.

Fermentation yield (kg *fp*·kg^−1^ sugars), titer (kg *fp*·m^−3^ broth), and productivity (kg *fp*·m^−3^ broth·h^−1^) may be expressed as follows:

1 $${{{\rm{yield}}}}=\frac{{\dot{m}}_{{fp}}}{{\dot{m}}_{{{{\rm{sugars}}}}}}$$

2 $${{{\rm{titer}}}}=\frac{{\dot{m}}_{{fp}}}{{Q}_{{{{\rm{broth}}}}}}$$

3 $${{{\rm{productivity}}}}=\frac{{{{\rm{titer}}}}}{{\tau }_{{{{\rm{fermentation}}}}}}$$

where ${\dot{m}}_{{fp}}$ is the mass flow rate of the fermentation product (e.g., 3HP), ${fp}$, effluent from the fermentation process (kg *fp*·h^−1^), ${\dot{m}}_{{{{\rm{sugars}}}}}$ is the mass flow rate of fermentable sugars diverted to the fermentation process (kg sugars·h^−1^), ${Q}_{{{{\rm{broth}}}}}$ is the volumetric flow rate of the broth effluent from the fermentation process (m^3^ broth·h^−1^), and ${\tau }_{{{{\rm{fermentation}}}}}$ is the fermentation time (h).

**Fig. 2:**
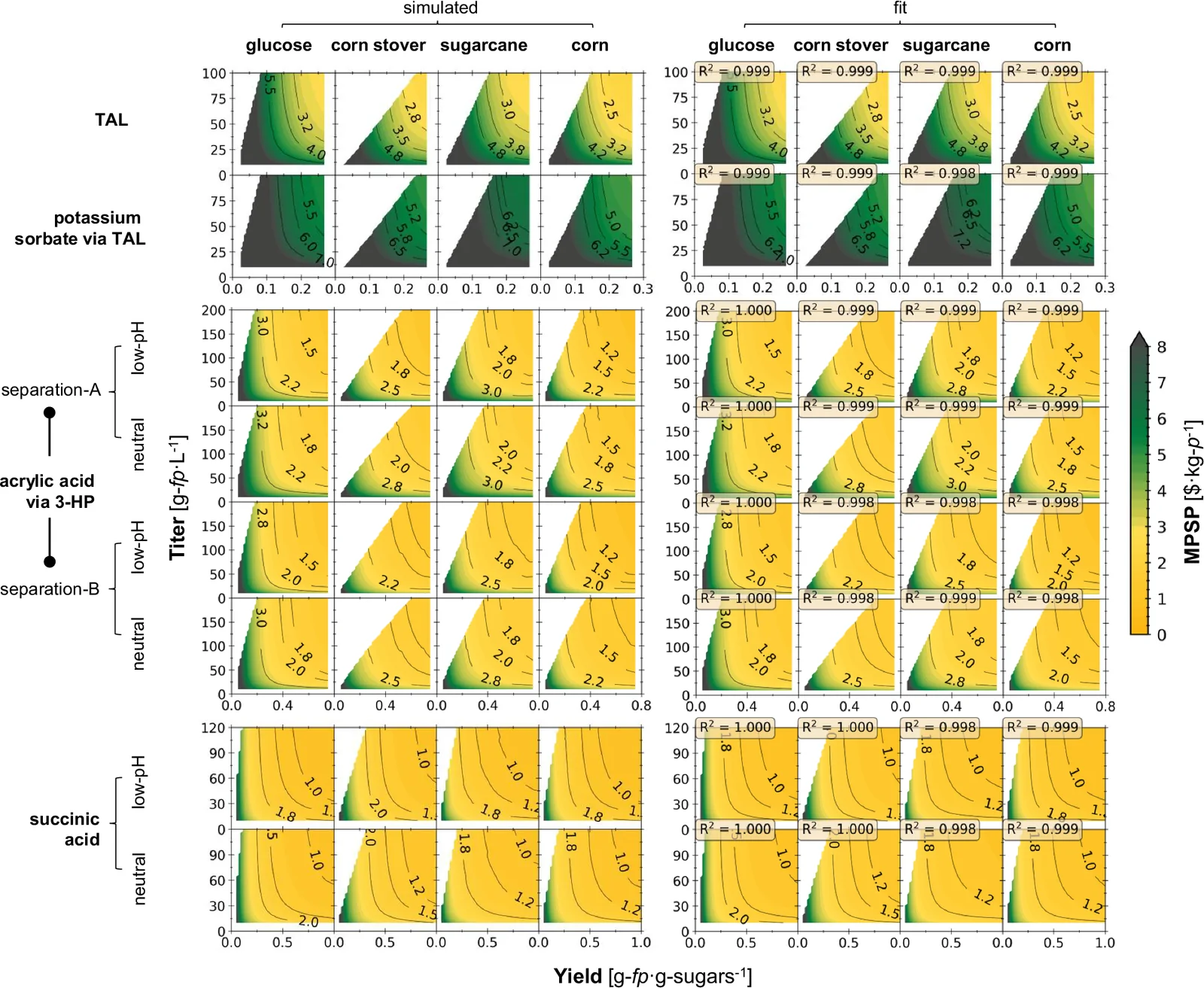
Minimum product selling price against fermentation yield and titer for 32 configurations from simulation and regression. Minimum product selling price (*MPSP*) of the main biomanufacturing facility product (*p*) across fermentation yields (*x*-axes) and titers (*y*-axes) at baseline productivity values (0.12 g-*fp*·L^−1^·h^−1^ for triacetic acid lactone biomanufacturing facilities, 0.28 g-*fp*·L^−1^·h^−1^ for 3-hydroxypropionic acid biomanufacturing facilities, and 0.66 g-*fp*·L^−1^·h^−1^ for succinic acid biomanufacturing facilities) for fermentation products (*fp*). For a given point on the figure, the *x-*axis value represents the overall fermentation yield (g-*fp*·g-sugars^−1^), the *y-axis* value represents the titer (g-*fp*·L^−1^), and the color represents MPSP. The white region to the upper left of each plot represents infeasible yield-titer combinations (described in Supplementary Note 1.1 in the Supplementary Information). Panels in the first four columns show results from simulation and evaluation of biomanufacturing facilities at 2500 fermentation yield-titer combinations each, while panels in the last four columns show results from fitting the empirical coefficients a, b, c, and d in Eq. 4 to the results from simulation and evaluation (maximizing *R*^2^, shown to the upper left of each figure; baseline values of empirical coefficients are provided in Supplementary Data 2). Rows represent biomanufacturing facilities producing triacetic acid lactone (*TAL*), potassium sorbate via triacetic acid lactone (*potassium sorbate* via *TAL*), acrylic acid via 3-hydroxypropionic acid (*acrylic acid* via *3HP*) under low-pH (*low-pH*) and neutral fermentation regimes (*neutral*) with separation process A (*separation-A*)^22^ or B (*separation-B*)^12^, and succinic acid (*succinic acid*) under low-pH (*low-pH*) and neutral fermentation regimes (*neutral*). Columns represent biomanufacturing facilities accepting dextrose monohydrate (*glucose*), sugarcane (*sugarcane*), corn stover (*corn stover*), and corn (*corn*) as the feedstock. All biomanufacturing facility configurations are described in Fig. 1. Source data for this figure are available in the *Source Data* file.

First, we simulated all 32 biomanufacturing facilities at 7500 distinct fermentation yield-titer-productivity combinations each, and estimated (by TEA) the minimum product selling price (MPSP, $·kg-*p*^−1^) of the main biomanufacturing facility product p derived from the fermentation product $${fp}$$ at each point (32 left-hand side panels in Fig. 2 and Supplementary Figs. 1 and 2 in the Supplementary Information). For all 32 biomanufacturing facilities, at constant productivity values, we found the following equation to describe the influence of fermentation yield and titer on the MPSP displayed remarkable goodness of fit to the results from evaluation across fermentation yield and titer (coefficient of determination, *R*^2^ of 0.992 − 1.000 across 7500 yield-titer-productivity combinations each for 32 biomanufacturing facilities; 32 right-hand side panels in Fig. 2 and Supplementary Figs. 1 and 2 in the Supplementary Information):

4 $${{{\rm{MPSP}}}}=a+\frac{b}{{{{\rm{yield}}}}}+\frac{c}{{{{\rm{titer}}}}}+\frac{d}{{{{\rm{yield}}}}*{{{\rm{titer}}}}}$$

where a, b, c, and d are empirical coefficients (baseline values from fitting are provided in Supplementary Data 2), and $${{{\rm{MPSP}}}}$$ is the minimum product selling price ($·kg-*p*^−1^) of the main biomanufacturing facility product p derived from the fermentation product $${fp}$$. Note $$p={fp}$$ if $${fp}$$ is not catalytically upgraded (e.g., for biomanufacturing facilities with p as TAL or succinic acid). Fermentation yield-titer combinations were simulated with productivities fixed at baseline values (0.12 g-*fp*·L^−1^·h^−1^ for TAL biomanufacturing facilities, 0.28 g-*fp*·L^−1^·h^−1^ for 3HP biomanufacturing facilities, and 0.66 g-*fp*·L^−1^·h^−1^ for succinic acid biomanufacturing facilities from baseline values reported in previous studies^10,16,18^ for current state-of-technology assumptions; Fig. 2), one-fifth the baseline values (Supplementary Fig. 1 in the Supplementary Information), and five times the baseline values (Supplementary Fig. 2 in the Supplementary Information).

Rearranging the terms in Eq. 4, we obtain:

5 $$\left({{{\rm{yield}}}}-\frac{b}{{{{\rm{MPSP}}}}-a}\right)\left({{{\rm{titer}}}}-\frac{c}{{{{\rm{MPSP}}}}-a}\right)=\frac{d+\frac{b*c}{{{{\rm{MPSP}}}}-a}}{{{{\rm{MPSP}}}}-a}$$

**Fig. 3:**
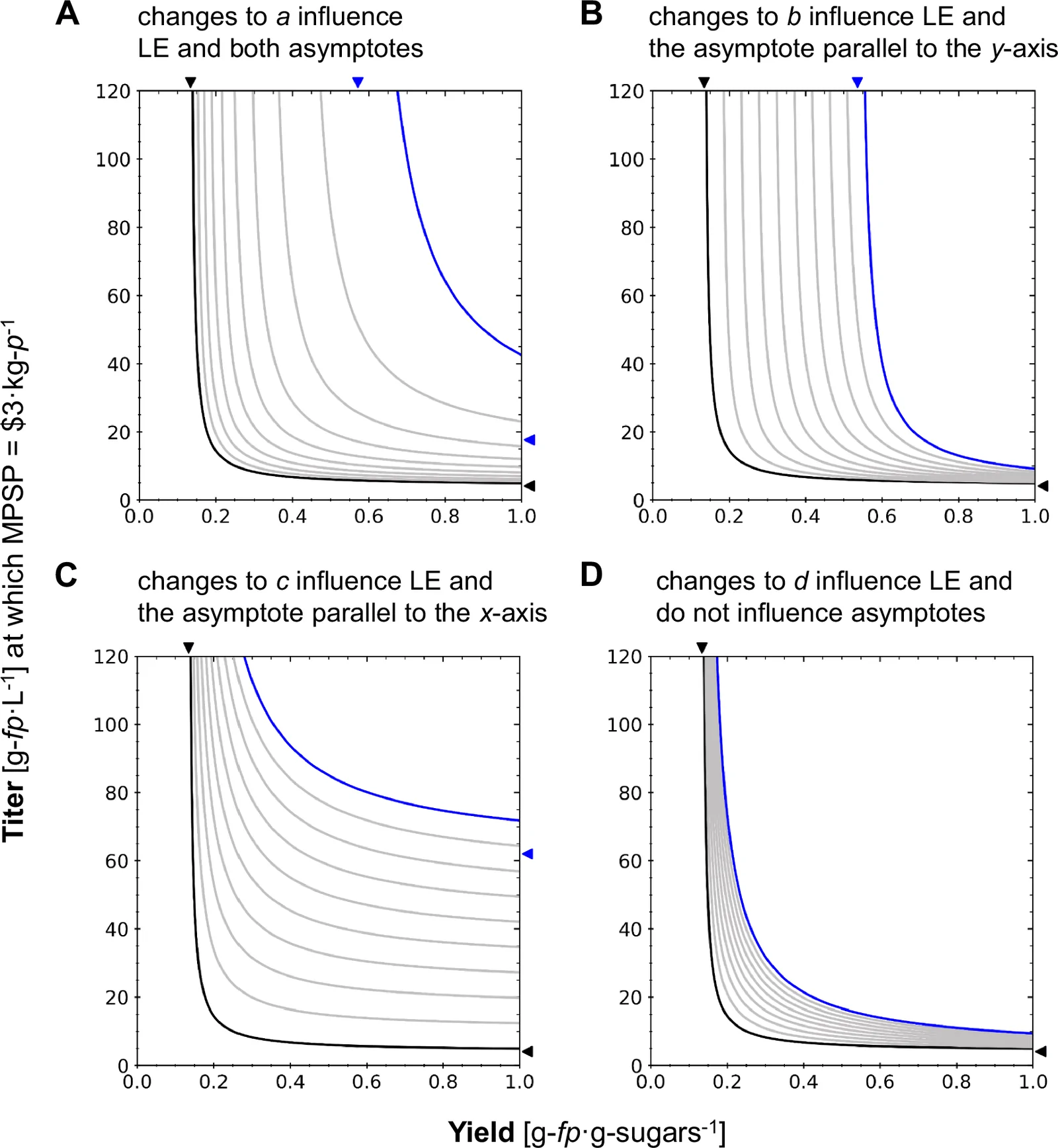
Geometric interpretation of the relationship between minimum product selling price and fermentation performance. Rectangular hyperbolae (black lines) representing the fermentation titer (*y*-axes) required to achieve a minimum product selling price (*MPSP*) of $3∙kg-*p*^−1^ at alternative values for fermentation yield (*x*-axes) for low-pH glucose-to-succinic acid biomanufacturing facilities (a = 0.31, b = 0.36, c = 10.84, and d = 0.54) and the associated asymptotes (black triangle markers). Rectangular hyperbolae (blue lines) representing the fermentation titer (*y*-axes) required to achieve an MPSP of $3∙kg-*p*^−1^ at alternative values for fermentation yield (*x*-axes) under hypothetical changes to biomanufacturing facility coefficients (**A**) a, (**B**) b, (**C**) c, and (**D**) d (by factors of 7.5, 4.0, 15.0, and 30.0, respectively, for demonstration purposes), and the associated asymptotes (blue triangle markers) if changed relative to glucose-to-succinic acid biomanufacturing facilities. Rectangular hyperbolae (gray lines) representing the fermentation titer (*y*-axes) required to achieve an MPSP of $3∙kg-*p*^−1^ at alternative values for fermentation yield (*x*-axes) under eight values for coefficients (**A**) a, (**B**) b, (**C**) c, and (**D**) d intermediate between those for the black hyperbolae and blue hyperbolae. The asymptotes (Supplementary Equations 1.3.1 and 1.3.2) determine the position of each hyperbola, and the linear eccentricity (LE; Supplementary Equation 1.3.3 in the Supplementary Equation) determines the shape of each hyperbola.

Equation 5 represents a rectangular hyperbola with asymptotes parallel to the titer and yield axes (Supplementary Equations 1.3.1 and 1.3.2 in the Supplementary Information) and the center shifted from the origin to $\left(\frac{b}{{{{\rm{MPSP}}}}-a},\frac{c}{{{{\rm{MPSP}}}}-a}\right)$. The shape of the hyperbola is determined by its linear eccentricity, $2*\sqrt{(\frac{d+\frac{b*c}{{{{\rm{MPSP}}}}-a}}{{{{\rm{MPSP}}}}-a})}$. The shape of the hyperbola depends on the ${{{\rm{MPSP}}}}$ with which it is associated as well as the values of all four coefficients a, b, c, and d (Fig. 3). In addition, changes in the value of b shift the hyperbola’s center along the yield axis, changes in the value of c shift the center along the titer axis, and changes to either a or ${{{\rm{MPSP}}}}$ shift the center along both axes (Fig. 3; the influence of coefficients on the shape of the hyperbolae described by Eq. 5 is discussed further in Supplementary Note 1.3 in the Supplementary Information.).

To assess the relative suitability of the developed equation, we compared our approach against two alternative regression approaches: (i) three-coefficient multivariate linear regression with fermentation yield and titer as variables (Supplementary Fig. 9 in the Supplementary Information); and (ii) adding a non-linear term to the equation from (i) to obtain a four-coefficient equation with fermentation yield and titer as variables (Supplementary Fig. 10 in the Supplementary Information). Across simulation results for the 32 biomanufacturing configurations, fitting the developed equation (Eq. 4) resulted in a substantially and consistently higher goodness of fit (coefficient of determination *R*^2^ of 0.992–1.000; Fig. 2 and Supplementary Figs. 1 and 2 in the Supplementary Information) than the two alternative regression approaches (R^2^ of 0.573–0.704 and 0.586–0.838, respectively; Supplementary Figs. 9 and 10 in the Supplementary Information), indicating the developed equation (Eq. 4) represented the wide design and thermodynamic simulation landscape across biomanufacturing configurations more robustly than the alternative approaches evaluated.

Note that despite the generally high goodness of fit (R^2^ of 0.992–1.000 across 32 biomanufacturing configurations; Fig. 2 and Supplementary Figs. 1 and 2 in the Supplementary Information), hotspots did appear where the developed equation did not capture the relationship between fermentation TRY and MPSP as robustly (Supplementary Fig. 8 in the Supplementary Information). For simulations across titer-yield spaces at baseline productivity values (Fig. 2), the absolute MPSP error (calculated as fractional absolute residual values; i.e., the ratio of the absolute residual MPSP value between the simulation and fitting results to the MPSP value from simulation) was 0.9% (median) with a range of 0.0–3.4% [5th–95th percentiles, hereafter in brackets] for biomanufacturing facilities producing TAL or potassium sorbate via TAL, 0.8% [0.0–2.7%] for biomanufacturing facilities producing acrylic acid via 3HP, and 1.0% [0.0%–2.8%] for biomanufacturing facilities producing succinic acid. The developed equation was not substantially biased toward either under-predicting or over-predicting the MPSP, with an MPSP error (calculated as signed fractional residual values rather than absolute values) of 0.0% [−2.4 to +3.3%] for biomanufacturing facilities producing TAL or potassium sorbate via TAL, 0.0% [−2.3% to +2.1%] for biomanufacturing facilities producing acrylic acid via 3HP, and 0.0% [−2.5% to +2.1%] for biomanufacturing facilities producing succinic acid (Supplementary Fig. 8 in the Supplementary Information). Notably, outlier absolute errors of 10% or higher were found at sixteen points among the 80,000 points simulated, specifically for biomanufacturing facilities producing TAL or potassium sorbate via TAL from glucose, specifically. All sixteen points were at four combinations of high values for titer (~100 g∙L^−1^) and low values for yield (~0.05 g∙g^−1^ glucose), indicating the error may be due to changes in the optimal configurations of heat exchanger networks designed at those points due to higher evaporation heating duties, resulting in step changes in system heating utility demands at those four combinations relative to other areas in the evaluated titer-yield spaces.

### Physical interpretation of equation coefficients and cost drivers under uncertainty

Note the overall post-fermentation recovery, r, of the main product p of a biomanufacturing facility may be expressed as follows:

6 $$r=\frac{{\dot{m}}_{p}}{{\dot{m}}_{{fp}}}$$

where ${\dot{m}}_{p}$ is the mass flow rate of the main product p of the biomanufacturing facility in units of kg-*p*·h^−^^1^. Note *r* is in units of kg-*p*·kg-*fp*^−1^. Consider the alternative coefficients:

7 $${k}_{Y}=a*r$$

8 $${k}_{0}=b*r$$

9 $${k}_{V}=c*r$$

10 $${k}_{T}=d*r$$

Then, we can recast Eqs. 4, 7, 8, 9, and 10 as:

11 $${{{\rm{MPSP}}}}=\frac{{k}_{Y}+\frac{{k}_{0}}{{{{\rm{yield}}}}}+\frac{{k}_{V}}{{{{\rm{titer}}}}}+\frac{{k}_{T}}{{{{\rm{yield}}}}*{{{\rm{titer}}}}}}{r}$$

The coefficients $${k}_{Y},\,{k}_{0},\,{k}_{V},$$ and $${k}_{T}$$—in units of $·kg-*fp*^−1^, $·kg-sugars^−1^, $·m^−3^-broth, and $·kg-*fp*·m^−3^-broth·kg-sugars^−1^, respectively—are independent of $${\dot{m}}_{p}$$, enabling comparisons across biomanufacturing facilities with differing main biomanufacturing facility products. For biomanufacturing facilities with separation processes designed for a target product recovery (as for all 32 biomanufacturing facilities evaluated in this study), r does not change substantially with fermentation yield, titer, or productivity (Supplementary Figs. 4 and 6B in the Supplementary Information). Thus, Eq. 4 (a form of Eq. 11 that implicitly assumes r is constant across yield-titer combinations) resulted in a high goodness of fit with empirical coefficients *a, b, c*, and *d* across all biomanufacturing facilities (*R*^2^ of 0.992 − 1.000; Fig. 2 and Supplementary Figs. 1 and 2 in the Supplementary Information).

**Fig. 4:**
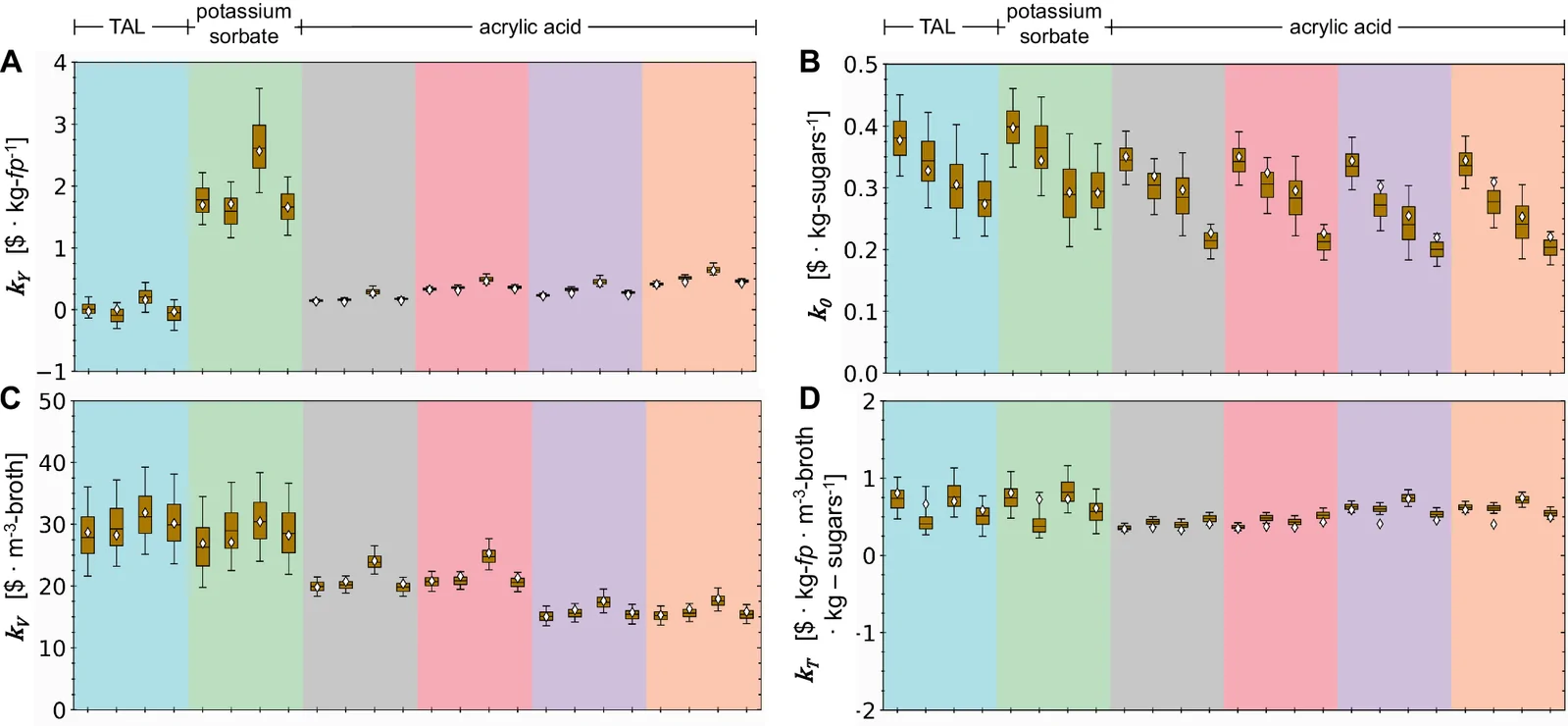
Uncertainty in equation coefficients from the results of 600,000 Monte Carlo simulations of 24 biomanufacturing configurations. Uncertainties (box-and-whisker plots) in the coefficients (**A**) ${k}_{Y},\,$(**B**) ${k}_{0},$ (**C**) ${k}_{V},$ and (**D**) ${k}_{T}$ in Eq. 11 from fitting to results to Monte Carlo simulations. Whiskers, boxes, and middle lines represent 5th/95th, 25th/75th, and 50th percentiles, respectively, from 1000 Monte Carlo simulations at each of 25 fermentation yield-titer combinations at baseline productivity values for each of 24 biomanufacturing facilities (i.e., 600,000 Monte Carlo simulations in total; *n* = 25,000 for each of the 24 plots shown). Plot background colors represent biomanufacturing facilities producing TAL as the main product (blue), biomanufacturing facilities producing potassium sorbate via TAL (green), biomanufacturing facilities producing acrylic acid via 3HP under low-pH (gray) and neutral fermentation regimes (pink) with separation process A, and biomanufacturing facilities producing acrylic acid via 3HP under low-pH (purple) and neutral fermentation regimes (orange) with separation process B. Within each background color, serially from left to right, box-and-whisker plots are shown for biomanufacturing facilities accepting dextrose monohydrate, corn stover, sugarcane, and corn as the feedstock. The main biomanufacturing facility product (*p*) is indicated above. Diamonds represent baseline values (used in Fig. 2; a full list of baseline coefficient values is provided in Supplementary Data 2). Source data for this figure are available in the *Source Data* file.

To elucidate key drivers that change how fermentation performance influences system cost, we performed uncertainty analyses at each of 25 fermentation yield-titer combinations for each of 24 biomanufacturing facilities, with 1000 Monte Carlo simulations (sampled by Latin hypercube sampling) at each yield-titer combination. For each Monte Carlo simulation, we fit coefficients ${k}_{Y},{k}_{0},{k}_{V}$ and ${k}_{T}$ to the MPSP evaluated at the 25 fermentation yield-titer combinations to obtain distributions of each coefficient for 24 biomanufacturing facilities (Fig. 4; full lists of baseline values, uncertainty distributions, and literature references for all parameters included in the uncertainty analyses are provided in Supplementary Data 3, and a detailed description on the choices of parameters’ distribution types and ranges is included in Supplementary Note 1.2 in the Supplementary Information).

Note the terms in Eq. 11 may be rearranged as follows:

12 $${{{\rm{MPSP}}}}=\frac{{\dot{m}}_{{{{\rm{sugars}}}}}*({k}_{Y}*{{{\rm{yield}}}}+{k}_{0}+\frac{{k}_{V}*{{{\rm{yield}}}}}{{{{\rm{titer}}}}}+\frac{{k}_{T}}{{{{\rm{titer}}}}})}{r*{\dot{m}}_{{{{\rm{sugars}}}}}*{{{\rm{yield}}}}}$$

As MPSP is in units of $·kg-*p*^−1^, the denominator of the right-hand side of Eq. 12 is in units of kg-*p*·y^−1^, and the numerator is in units of $·y^−1^ (note this includes production costs, loan interest, and return on investment). Therefore, the term $${\dot{m}}_{{{{\rm{sugars}}}}}*{k}_{Y}*{{{\rm{yield}}}}$$ represents biomanufacturing facility costs that increase linearly with fermentation product yield but do not change with titer. The term $${\dot{m}}_{{{{\rm{sugars}}}}}*{k}_{0}$$ represents biomanufacturing facility costs that are independent of both fermentation yield and titer. The term $${\dot{m}}_{{{{\rm{sugars}}}}}*\frac{{k}_{V}*{{{\rm{yield}}}}}{{{{\rm{titer}}}}}$$ represents biomanufacturing facility costs that increase linearly with the ratio of fermentation yield to titer. Finally, the term $${\dot{m}}_{{{{\rm{sugars}}}}}*\frac{{k}_{T}}{{{{\rm{titer}}}}}$$ represents biomanufacturing facility costs that increase linearly with the inverse of fermentation titer.

Using the results of the uncertainty analyses (Fig. 4), we performed global sensitivity analyses by estimating Spearman’s rank order correlation coefficients (Spearman’s *ρ*) and used them to identify parameters to which coefficients ${k}_{Y},\,{k}_{0},\,{k}_{V}$, and ${k}_{T}$ to were sensitive (i.e., |Spearman’s *ρ*| ≥ 0.10 and *p* value < 0.05) and insensitive (i.e., |Spearman’s *ρ*| < 0.10). By contextualizing Spearman’s *ρ* values and the results from the uncertainty analyses with Eq. 12, we elucidated key drivers of the relationship between fermentation performance and system cost.

Values of coefficient $${k}_{Y}$$ (the facility’s yield-dependent burden coefficient, which influences costs that increase linearly with fermentation yield, but not titer; Eq. 12) were consistently higher for neutral fermentation regimes than for low-pH fermentation regimes. For example, for biomanufacturing facilities producing acrylic acid via 3HP from corn with separation process A, $${k}_{Y}$$ was 0.15 $∙kg-*fp*^−1^ in the baseline case and had a range of 0.15−0.20 $∙kg-*fp*^−1^ [5th−95th percentiles from 1000 Monte Carlo simulations, hereafter in brackets] under low-pH fermentation regimes and an increased 0.33 [0.33−0.40] $∙kg-*fp*^−1^ under neutral fermentation regimes (Fig. 4A). Similarly, $${k}_{Y}$$ was consistently higher with the inclusion of catalytic upgrading. For example, $${k}_{Y}$$ was consistently higher for biomanufacturing facilities producing potassium sorbate via TAL ($${k}_{Y}$$ of 1.69 [1.38−2.22] $∙kg-*fp*^−1^, 1.71 [1.17−2.07] $∙kg-*fp*^−1^, 2.57 [1.89−3.58] $∙kg-*fp*^−1^, and 1.66 [1.20−2.15] $∙kg-*fp*^−1^) than biomanufacturing facilities producing TAL as the main product ($${k}_{Y}$$ of −0.03 [−0.14−0.21] $∙kg-*fp*^−1^, 0.01 [−0.30−0.12] $∙kg-*fp*^−1^, 0.16 [−0.04−0.44] $∙kg-*fp*^−1^, and −0.03 [−0.33−0.16] $∙kg-*fp*^−1^ for dextrose monohydrate, corn stover, sugarcane, and corn biomanufacturing facilities, respectively). Costs associated with both neutral fermentation and catalytic upgrading were expected to increase as the mass flow rate of the fermentation product $${\dot{m}}_{{fp}}$$ being neutralized or catalytically upgraded increased, resulting in an increase in those costs as fermentation yield increased. Further, $${k}_{Y}$$ was consistently higher for biomanufacturing facilities producing acrylic acid via 3HP using separation process B rather than A, as separation process B necessitated costs associated with throughput flow of 3HP rather than dilution water (solvent extraction was downstream of an evaporator that concentrated the crude 3HP stream to a fixed 26 wt% in separation process B^12^, whereas evaporation was the final step in separation process A prior to catalytic upgrading^22^). From the parameters included in the uncertainty analyses that were shared by all 24 biomanufacturing facilities (Supplementary Data 4), $${k}_{Y}$$ was consistently insensitive (i.e., |Spearman’s *ρ*| < 0.10) for all 24 biomanufacturing facilities to feedstock unit price, corn steep liquor unit price, and seed train yield relative to fermentation yield. This was consistent with none of these parameters being associated with biomanufacturing facility flows that were expected to increase as fermentation yield increased but not as titer increased (Supplementary Fig. 4C in the Supplementary Information). Therefore, fermentation regime (neutral vs. low-pH), catalytic upgrading, and the design of separations downstream of any initial separation of water from fermentation broths (to a target concentration of *fp*) were key drivers of system cost but did not influence the relationship between fermentation performance and system cost (governed by coefficient $${k}_{Y}$$; Eq. 12).

Values of coefficient $${k}_{0}$$ (the facility’s TRY-invariant burden coefficient, which influences costs that are independent of fermentation yield and titer; Eq. 12) were very similar for biomanufacturing facilities accepting the same feedstock. For example, $${k}_{0}$$ for corn stover biomanufacturing facilities was 0.33 [0.27−0.42] $·kg-sugars^−1^, 0.34 [0.29−0.45] $·kg-sugars^−1^, 0.32 [0.26−0.35] $·kg-sugars^−1^, 0.32 [0.26−0.35] $·kg-sugars^−1^, 0.30 [0.23−0.31] $·kg-sugars^−1^, and 0.31 [0.24−0.32] $·kg-sugars^−1^ (Fig. 4B). This was because feedstock capacities and unit prices were independent of fermentation yield and titer. The biogas recovered from anaerobic digestion of waste fractions of the feedstock in WWRR, as well as the steam utilities recovered by burning solid waste fractions of the feedstock (e.g., sugarcane bagasse) in CHP, were also independent of fermentation yield and titer. Further, for all 24 biomanufacturing facilities, $${k}_{0}$$ was consistently sensitive (i.e., |Spearman’s *ρ*| ≥ 0.10 and *p* value < 0.05) to feedstock unit price and to fermentation cell mass yield (Supplementary Data 5). In addition, $${k}_{0}$$ was sensitive to feedstock capacity (which influenced the production costs of the sugar solutions) in 22 of 24 biomanufacturing facilities (Supplementary Data 5), except for two dextrose monohydrate biomanufacturing facilities (as mixing for dilution was the only processing step required to produce sugar solutions from dextrose monohydrate). Baseline values and uncertainty distributions for fermentation cell mass yields were based on reported values and assumed to be independent of fermentation yield and titer, so the costs and revenues associated with burning centrifuged cell mass in the boiler combustion chamber to recover steam utilities did not change with fermentation yield or titer. In addition, $${k}_{0}$$ was consistently sensitive to boiler and turbogenerator efficiencies for all 18 sugarcane, corn stover, and corn biomanufacturing facilities but not for the four dextrose monohydrate biomanufacturing facilities (Supplementary Data 5). This was because sugarcane, corn stover, and corn were associated with higher energy waste streams diverted to WWRR and CHP than dextrose monohydrate. Values of $${k}_{0}$$ values for a given main biomanufacturing facility product were consistently the 1st, 2nd, 3rd, and 4th-greatest for dextrose monohydrate, corn stover, sugarcane, and corn, respectively (Fig. 4B), as the production costs of sugars from the acquisition, pretreatment, saccharification, and waste stream management of each of those feedstocks were independent of fermentation yield and titer. Finally, for all 24 biomanufacturing facilities, $${k}_{0}$$ was consistently insensitive to the natural gas unit price, corn steep liquor unit price, fermentation inoculum ratio, seed train yield relative to fermentation yield, and main product storage time (Supplementary Data 5). This was consistent with none of these parameters being associated with biomanufacturing facility flows that were expected to be independent of both fermentation yield and titer (Supplementary Fig. 4 in the Supplementary Information). Therefore, feedstock unit price, the recoverable energy in the waste fractions of the feedstock and fermentation broth, and the efficiency of energy recovery in WWRR and CHP were key drivers influencing the relationship between fermentation yield and system cost (governed by coefficient $${k}_{0}$$; Eq. 12).

Coefficient $${k}_{V}$$ (the facility’s broth volumetric flow-dependent burden coefficient, which influences costs that increase linearly with the ratio of fermentation yield to titer; Eq. 12) had similar values for biomanufacturing facilities with the same separation process, regardless of the inclusion of catalytic upgrading. For example, $${k}_{V}$$ was 31.88 [25.12–39.24] $∙m^−3^-broth for sugarcane biomanufacturing facilities producing TAL as the main product and 30.43 [24.01−38.36] $∙m^−3^-broth for sugarcane biomanufacturing facilities producing potassium sorbate via TAL (Fig. 4C). This was because the total amount of water from which the fermentation product (TAL for both biomanufacturing facilities) was separated and the total volume of the fermentation reactors were both expected to increase linearly with the ratio of fermentation yield to titer at a constant productivity (Supplementary Fig. 4D in the Supplementary Information), while the costs of catalytic upgrading increased with fermentation yield but not titer (discussed earlier). Similarly, $${k}_{V}$$ had similar values for biomanufacturing facilities producing acrylic acid via 3HP using separation process A, regardless of fermentation regime or feedstock choice (Fig. 4C). Further, under the same feedstock choice, biomanufacturing facilities using separation process A were associated with consistently higher values of $${k}_{V}$$ than separation process B, as separation process A included a greater number of unit operations upstream of water removal (after acidulation, gypsum removal, and cell mass removal, an evaporator concentrated the crude 3HP stream to a fixed 26 wt% in separation process B^12^, whereas color removal and ion exchange unit operations were placed prior to evaporation in separation process A^22^). For the same feedstock and fermentation product *fp*, values of $${k}_{V}$$ were slightly but consistently higher for neutral fermentation regimes than for low-pH fermentation regimes (Fig. 4C), as costs associated with the acidulation reactor and gypsum filter (equipment used in biomanufacturing facilities with neutral fermentation regimes) increased with the amount of water in the crude 3HP stream. For all 24 biomanufacturing facilities, $${k}_{V}$$ was consistently sensitive to corn steep liquor (CSL) loading (g-CSL∙L-broth^−1^) during fermentation as well as CSL unit price (Supplementary Data 6), as the total purchase cost of CSL (used as a nitrogen source in the fermentation medium) to maintain a specified CSL loading increased with the amount of water in the broth. In addition, although $${k}_{V}$$ did not change substantially under changes to feedstock choice, $${k}_{V}$$ values for a given main biomanufacturing facility product were consistently the highest for sugarcane and lowest for dextrose monohydrate, respectively (Fig. 4C), as the energy recovered from the waste fractions of feedstocks displaced costs associated with the purchase of energy used by separation processes. Finally, for all 24 biomanufacturing facilities, $${k}_{V}$$ was consistently insensitive to the feedstock unit price, electricity unit price, seed train yield relative to fermentation yield, and main biomanufacturing facility product storage time (Supplementary Data 6), as none of the biomanufacturing facility flows associated with these parameters were expected to increase with the ratio of fermentation yield to titer (Supplementary Fig. 4D in the Supplementary Information). Therefore, the unit prices and loadings for fermentation nutrient sources (such as CSL) and the design of separations, including and upstream of any initial separation of water from fermentation broths (to a target concentration of *fp*) were key drivers influencing the relationship between fermentation titer and system cost (governed by coefficient $${k}_{V}$$; Eq. 12).

In addition, the use of dextrose monohydrate, corn stover, and corn were associated with relatively similar values for $${k}_{Y}$$ for a given main product *p* (baseline$$\,{k}_{Y}$$ values for corn stover and corn differed by at most 18% and 74%, respectively, relative to those for dextrose monohydrate, with the latter representing an absolute difference of 0.03 $∙kg-*fp*^−1^) and relatively similar $${k}_{V}$$ values for a given fermentation product *fp* (baseline $${k}_{V}$$ values for corn and corn stover differed by at most 5% and 7%, respectively, relative to those for dextrose monohydrate), but the use of sugarcane was associated with increased $${k}_{Y}$$ and $${k}_{V}$$ values (baseline values increased by up to 470% and 22%, respectively, relative to values for glucose; Fig. 4 and Supplementary Data 2). This was because the biomanufacturing facility’s operating time was lower for sugarcane (baseline value of 180 d∙y^−1^) than for corn, glucose, or corn stover biomanufacturing facilities (baseline value of 330 d∙y^−1^), resulting in an increased contribution of all equipment purchase costs to the MPSP. This was consistent with both $${k}_{Y}$$ and $${k}_{V}$$ being consistently sensitive to operating time for all 8 sugarcane biomanufacturing facilities (Supplementary Data 4 and 6; full lists of baseline values, uncertainty distributions, and literature references for all parameters included in the uncertainty analyses are provided in Supplementary Data 3).

Across the 24 biomanufacturing facilities evaluated under uncertainty, $${k}_{T}$$ (the facility’s titer-only dependent burden coefficient, which influences costs that depend on titer but not on yield) ranged between 0.22 and 1.16 $·kg-*fp*·m^−3^-broth·kg-sugars^−1^ (minimum of 5th percentiles and maximum of 95th percentiles, respectively, from 600000 Monte Carlo simulations; Fig. 4D). Over the evaluated theoretical fermentation space for all 32 biomanufacturing facilities at the baseline productivity (Fig. 2), the contribution of the term $$\frac{{k}_{T}}{r*{{{\rm{yield}}}}*{{{\rm{titer}}}}}$$ to the MPSP in Eq. 11 ranged from 0.3%−5.1% (5th−95th percentiles from simulations across titer and yield for all biomanufacturing facilities; Supplementary Fig. 5 in the Supplementary Information), indicating $${k}_{T}$$ did not substantially influence the relationship between fermentation performance and system cost.

At a fixed feedstock capacity (as with each biomanufacturing configuration in the baseline case), when fermentation productivity changes at a fixed titer and yield, only fermentation time changes among all parameters described in Eqs. 1, 2, and 3, limiting the influence of productivity on MPSP to costs associated with the purchase and installation of fermentation bioreactors and the power utility demanded for mixing bioreactor contents (Supplementary Fig. 11 in the Supplementary Information). We found the following equations described the influence of fermentation productivity on the coefficients ${k}_{Y}$, ${k}_{0}$, ${k}_{V}$, and ${k}_{T}$ (multiplying coefficient values with their associated productivity values resulted in straight lines not crossing the origin when plotted against productivity for biomanufacturing facilities producing potassium sorbate via TAL from sugarcane; Supplementary Fig. 6 in the Supplementary Information):

13 $${k}_{Y}={a}_{1}+\frac{{a}_{2}}{{{{\rm{productivity}}}}}\,$$

14 $${k}_{0}={b}_{1}+\frac{{b}_{2}}{{{{\rm{productivity}}}}}$$

15 $${k}_{V}={c}_{1}+\frac{{c}_{2}}{{{{\rm{productivity}}}}}$$

16 $${k}_{T}={d}_{1}+\frac{{d}_{2}}{{{{\rm{productivity}}}}}$$

where ${a}_{1}$ and ${a}_{2}$, ${b}_{1}$ and ${b}_{2}$, ${c}_{1}$ and ${c}_{2}$, and ${d}_{1}$ and ${d}_{2}$ can replace empirical coefficients ${k}_{Y}$, ${k}_{0}$, ${k}_{V}$, and ${k}_{T}$, respectively, in Eq. 11, resulting in the following equation to capture the relationship between MPSP and fermentation yield, titer, and productivity:

17 $${{{\rm{MPSP}}}}=\frac{{a}_{1}\,+\,\frac{{a}_{2}}{{{{\rm{productivity}}}}}\,+\,\frac{{b}_{1}+\frac{{b}_{2}}{{{{\rm{productivity}}}}}}{{{{\rm{yield}}}}}\,+\,\frac{{c}_{1}+\frac{{c}_{2}}{{{{\rm{productivity}}}}}}{{{{\rm{titer}}}}}\,+\,\frac{{d}_{1}+\frac{{d}_{2}}{{{{\rm{productivity}}}}}}{{{{\rm{yield}}}}*{{{\rm{titer}}}}}}{r}$$

### Relative importance of fermentation yield and titer for system cost

The developed mathematical equation enables defining and estimating alternative metrics to help prioritize needs in early-stage fermentation research and development. Here, we propose a metric to quantify the relative importance of fermentation yield and titer improvements for MPSP (${{RI}}_{{MPSP}}$) as follows:

18 $${{{{\rm{RI}}}}}_{{{{\rm{MPSP}}}}}={\log }_{10}\frac{\partial ({{{\rm{MPSP}}}})/\partial ({{{\rm{yield}}}})}{\partial ({{{\rm{MPSP}}}})/\partial ({{{\rm{titer}}}})}$$

From Eqs. 4 and 18, we obtain the following:

19 $${{{{\rm{RI}}}}}_{{{{\rm{MPSP}}}}}={\log }_{10}\frac{{{{\rm{tite}}}}{r}^{2}*\left(b+\frac{d}{{{{\rm{titer}}}}}\right)}{{{{{\rm{yield}}}}}^{2}*\left(c+\frac{d}{{{{\rm{yield}}}}}\right)}$$

Effectively, Eq. 19 evaluates the benefit to MPSP from a unit increment to fermentation yield (e.g., 1 g-*fp*∙g-sugars^−1^) relative to the benefit to MPSP from a unit increment to titer (e.g., 1 g-*fp*∙L^−1^), with higher ${{{{\rm{RI}}}}}_{{{{\rm{MPSP}}}}}$ values implying a greater change from incremental improvements to yield and lower ${{{{\rm{RI}}}}}_{{{{\rm{MPSP}}}}}$ implying a greater change from incremental improvements to titer. To evaluate ${{{{\rm{RI}}}}}_{{{{\rm{MPSP}}}}}$ with alternative increments for fermentation yield (e.g., 0.01; g-*fp*∙g-sugars^−1^) and titer, the units of fermentation yield, titer, and coefficients b,c, and d must be changed accordingly (a does not influence ${{{{\rm{RI}}}}}_{{{{\rm{MPSP}}}}}$). Note the coefficients $a,{b},c,$ and d in Eq. 19 may readily be replaced with coefficients ${k}_{Y},\,{k}_{0},{k}_{V},$ and ${k}_{T}$ using Eq. 7−10.

**Fig. 5:**
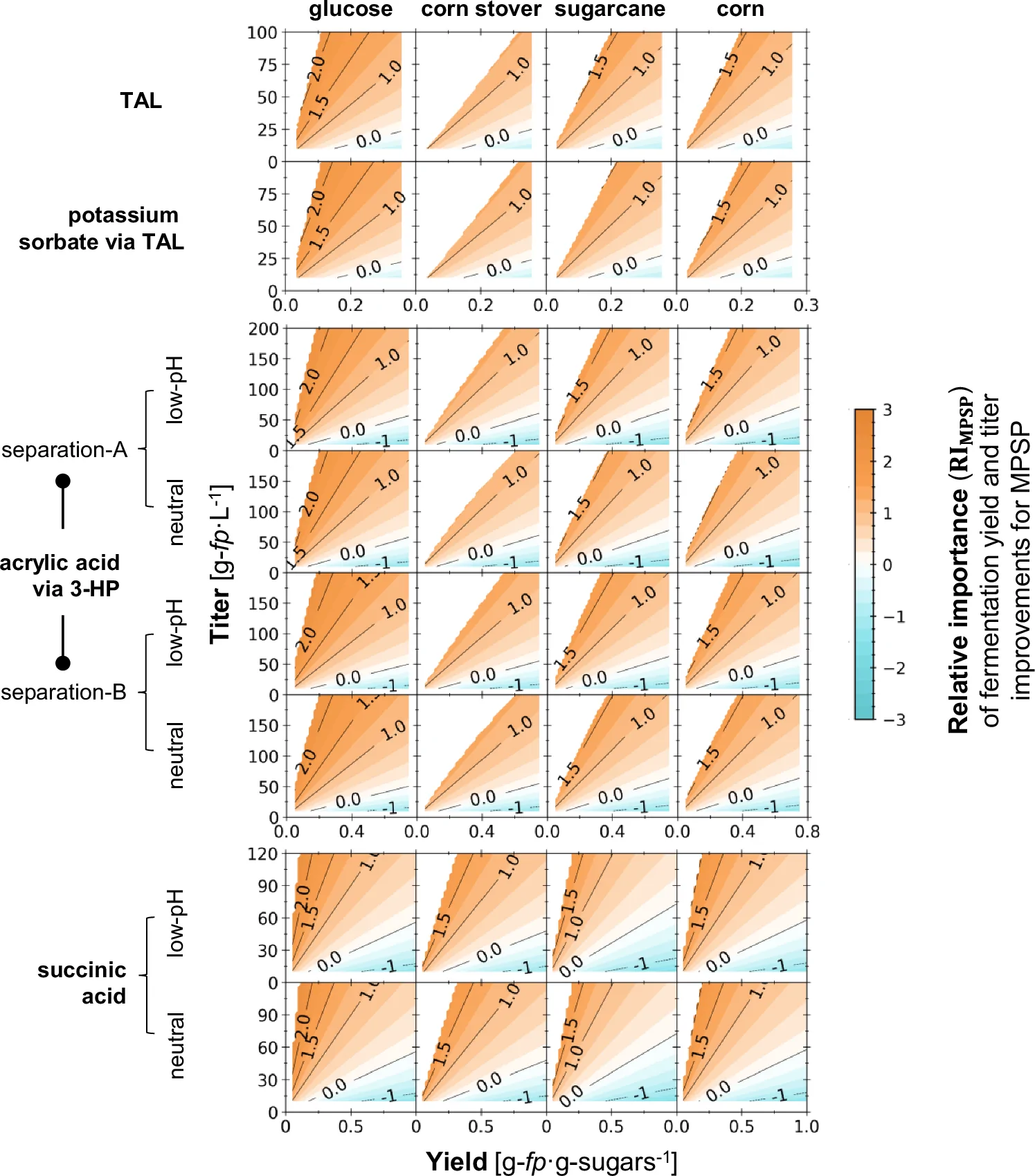
Relative importance of fermentation yield and titer for facility-scale economics. Relative importance of fermentation yield and titer improvements for minimum product selling price (${{RI}}_{{MPSP}}$; Eq. 19) using baseline values for coefficients ${k}_{Y},\,{k}_{0},{k}_{V},$ and ${k}_{T}$ (Supplementary Data 2) across fermentation yields (*x*-axes) and titers (*y*-axes) at baseline productivity values (0.12 g-*fp*·L^−1^·h^−1^ for TAL biomanufacturing facilities, 0.28 g-*fp*·L^−1^·h^−1^ for 3HP biomanufacturing facilities, and 0.66 g-*fp*·L^−1^·h^−1^ for succinic acid biomanufacturing facilities). To estimate ${{RI}}_{{MPSP}}$, yield units were in 0.01 g-*fp*·g-sugars^−1^ and titer units were in g-*fp*·L^−1^ (i.e., at each yield-titer combination, the benefit to minimum product selling price (*MPSP*) from a yield increment of 0.01 g-*fp*·g-sugars^−1^ was compared to that from a titer increment of 1 g-*fp*·L^−1^). For a given point on the figure, the *x-*axis value represents the overall fermentation yield (g-*fp*·g-sugars^−1^), the *y-axis* value represents the titer (g-*fp*·L^−1^), and the color represents ${{RI}}_{{MPSP}}$. The white region to the upper left of each plot represents infeasible yield-titer combinations (described in Supplementary Note 1.1 in the Supplementary Information). Rows represent biomanufacturing facilities producing TAL (*TAL*), potassium sorbate via TAL (*potassium sorbate* via *TAL*), acrylic acid via 3HP (*acrylic acid* via *3HP*) under low-pH (*low-pH*) and neutral fermentation regimes (*neutral*) with separation process A (*separation-A*)^22^ or B (*separation-B*)^12^, facilities producing succinic acid (*succinic acid*) under low-pH (*low-pH*) and neutral fermentation regimes (*neutral*). Columns represent biomanufacturing facilities accepting dextrose monohydrate (*glucose*), sugarcane (*sugarcane*), corn stover (*corn stover*), and corn (*corn*) as the feedstock. All biomanufacturing facility configurations are described in Fig. 1. Source data for this figure are available in the **Source Data** file.

For all 32 biomanufacturing facilities (Fig. 1), we used baseline values of $a,{b},{c},$ and d with Eq. 19 to estimate ${{{{\rm{RI}}}}}_{{{{\rm{MPSP}}}}}$ across the theoretical fermentation performance space (Fig. 5). We observed ${{RI}}_{{MPSP}}$ was higher at higher titer values (e.g., at a yield of 0.2 g-*fp*∙g-sugars^−1^, ${{{{\rm{RI}}}}}_{{{{\rm{MPSP}}}}}$ was 0.11 at 20 g-*fp*∙L^−1^ and 1.28 at 80 g-*fp*∙L^−1^ for biomanufacturing facilities producing TAL from dextrose monohydrate), and lower at higher yield values (e.g., at a titer of 20 g-*fp*∙L^−1^, ${{{{\rm{RI}}}}}_{{{{\rm{MPSP}}}}}$ was 0.11 at 0.2 g-*fp*∙g-sugars^−1^ and −0.23 at 0.3 g-*fp*∙g-sugars^−1^ for biomanufacturing facilities producing TAL from dextrose monohydrate; Fig. 5).

Across all 32 biomanufacturing facilities, the benefit to MPSP from an incremental improvement to yield (by 0.01 g-*fp* ∙ g-sugars^−1^) outweighed that from an incremental improvement to titer (by 1 g-*fp* ∙ L^−1^) in the majority fractions of the evaluated theoretical fermentation spaces (i.e., ${{{{\rm{RI}}}}}_{{{{\rm{MPSP}}}}} > 0$ for 71−95% of each of the evaluated theoretical fermentation spaces; Fig. 5). For biomanufacturing facilities with the same fermentation product and feedstock, the fraction of the theoretical fermentation space where ${{{{\rm{RI}}}}}_{{{{\rm{MPSP}}}}} > 0$ remained virtually unchanged, regardless of fermentation regime (e.g., 82% for biomanufacturing facilities producing succinic acid from corn stover under neutral or low-pH regimes) or the inclusion of catalytic upgrading (e.g., 90% for biomanufacturing facilities producing TAL or producing potassium sorbate via TAL from sugarcane; Fig. 5). This was consistent with ${k}_{0}$ and ${k}_{T}$ remaining relatively unchanged and ${k}_{V}$ only changing slightly under changes to these parameters (Fig. 4B, C, D). The fraction of the theoretical fermentation space where ${{{{\rm{RI}}}}}_{{{{\rm{MPSP}}}}} > 0$ was substantially different for biomanufacturing facilities accepting different feedstocks (e.g., 82% and 71% for biomanufacturing facilities producing succinic acid under low-pH fermentation from corn stover and sugarcane, respectively) regardless of other similarities, as ${k}_{0}$ and ${k}_{V}$ both changed with feedstock choice (Fig. 4B, C). Although ${{{{\rm{RI}}}}}_{{{{\rm{MPSP}}}}}$ will change under alternative increments to fermentation yield and titer, the trends described in this section will hold generally and may be used to prioritize needs in early-stage fermentation research and development.

### Validation with external studies and alternative process simulators

The 32 biomanufacturing facilities used to extract the unifying equation proposed in this work (simplified form shown in Eq. 4; the form used for physical interpretation of coefficients is shown in Eq. 12) were all developed in BioSTEAM by the same authors and involved three fermentation products (triacetic acid lactone, 3-hydroxypropionic acid, and succinic acid) and four final products (triacetic acid lactone, potassium sorbate via triacetic acid lactone, acrylic acid via 3-hydroxypropionic acid, and succinic acid) from four feedstocks (dextrose monohydrate, sugarcane, corn, and corn stover) involving four distinct separation processes (overview of these 32 biomanufacturing configurations shown in Fig. 1). To further ascertain the generalizability of the proposed equation, we also sought to validate results using data from external studies that explored biomanufacturing configurations with different fermentation products and separation processes using different process simulator tools (e.g., Aspen Plus, SuperPro Designer).

Aside from studies published by the authors of this study, we found three previous studies that simulated biomanufacturing facilities across multiple combinations of fermentation titer and/or yield and estimated MPSP at those combinations. Using SuperPro Designer, Gunukula et al. ^19^ designed biomanufacturing facilities: (i) producing isobutanol from corn using an isobutanol separation process that involved distillation and decantation; (ii) producing 3HP from corn using a 3HP separation process involving solvent extraction (using methyl isobutyl ketone) and distillation; (iii) producing 1,3-propanediol from corn under microaerobic fermentation conditions and using a 1,3-propanediol separation process that involved solvent extraction (using hexanol) and distillation; and (iv) producing 1,3-propanediol from corn under aerobic fermentation conditions and using the same separation process as (iii). Gunukula et al. simulated the biomanufacturing facilities across fermentation titer and yield of the respective fermentation products and estimated the MPSP at each simulated combination of fermentation yield and titer. Using Aspen Plus, Janković et al. ^23^ designed a biomanufacturing facility separating pure ethanol from a crude ethanol stream through a process involving vacuum distillation and extractive distillation (using ethylene glycol), and estimated the annual cost of separation across alternative values for ethanol titer in the crude stream. Finally, using Aspen Plus, Sikazwe et al. ^20^ designed a biomanufacturing facility producing 2,3-butanediol from sugarcane A-molasses that used a 2,3-butanediol separation process involving solvent extraction (using oleyl alcohol) and distillation, and estimated the MPSP across alternative combinations of fermentation yield and titer.

We fit Eq. 4 to the results for each of these six biomanufacturing configurations that external studies had simulated and evaluated to estimate MPSP across fermentation yield and/or titer. Across this diverse design and simulation landscape explored using Aspen Plus and SuperPro Designer in the external studies, we found a high goodness of fit (coefficient of determination *R*^2^ of 0.954–1.000; Supplementary Fig. 7 in the Supplementary Information), comparable to the goodness of fit for the 32 biomanufacturing configurations simulated in this work (*R*^2^ of 0.992–1.000). This demonstrates that the unifying equation proposed in this work is generalizable across nth plant TEAs with process designs involving diverse feedstocks, fermentation processes and products, separation processes, and catalytic upgrading regimes to produce commodity chemicals.

### Key considerations for equation applicability

Despite the robustness of the developed equation across vast design and thermodynamic landscapes simulated using multiple process simulators (BioSTEAM, Aspen Plus, and SuperPro Designer), it is worth noting a few considerations regarding the equation’s applicability:

(i). All products of the biomanufacturing facilities explored in this work (triacetic acid lactone, potassium sorbate via triacetic acid lactone, acrylic acid via 3-hydroxypropionic acid, and succinic acid), as well as those explored in the external studies used for validation (ethanol, isobutanol, 1,3-propanediol, 3-hydroxypropionic acid, and 2,3-butanediol), were commodity chemicals simulated with large annual production capacities (>20,000 metric ton∙y^−1^). Although feedstock capacity uncertainties were included in all uncertainty analyses performed in this work (Supplementary Data 3), extending the proposed equation to smaller-scale facilities producing specialty chemicals, pharmaceuticals, and proteins may result in poor goodness of fit relative to the large-scale facilities explored in this work as the exponentially scaling equipment purchase and installation costs are larger contributors to the MPSP at smaller scales and may not be adequately captured by the simple equation proposed in this work.
(ii). As is typical for biomanufacturing TEAs, this work and all external studies used for validation were performed *n*th plant TEA (i.e., it is assumed a successful industry has been established with mature technologies and supply chains). Established methods were used in this work for process design, simulation, and equipment sizing and costing, and rigorous uncertainty and sensitivity analyses were performed to avoid false precision^21,24^. However, it is worth noting that *n*th plant TEAs may not comprehensively capture the actual industrial costs experienced, especially by pioneer facilities (i.e., <*n*th plant). Such facilities navigate several challenges, including navigating technological and scale-up hurdles, developing the workforce, establishing new supply chains and markets, and operating under highly variable conditions, thereby contributing toward maturing a nascent biomanufacturing industry.
(iii). This work was focused on economic indicators of sustainability and did not explore environmental impacts such as carbon intensity (e.g., as 100-year global warming potential) or fossil energy consumption. However, the proposed unifying equation could potentially be used to capture the relationship between a biomanufacturing facility’s environmental impacts (estimated via life cycle assessment, LCA) and fermentation performance, as previously reported life cycle inventory data were based on each facility’s material and energy balance flows from process design, simulation, and TEA^10,12,16,18,25^. A simplified form of the equation may be appropriate for life cycle impacts, as these typically exclude equipment acquisition and installation (consistent with carbon intensity accounting requirements in the U.S. Renewable Fuel Standard, RFS^26^). Consequently, it may not be necessary to represent the non-linear relationships associated with the scaling of equipment purchase and installation costs. However, future work to explore suitably modifying the proposed mathematical equation to capture how the biomanufacturing facility’s life cycle environmental impacts are influenced by fermentation performance will be critical to navigating economic and environmental trade-offs.

## Discussion

In this study, we proposed a simple, physically interpretable mathematical equation (Eq. 12; further simplified form shown in Eq. 4) that robustly captures the relationship between fermentation performance (titer, rate or productivity, and yield, TRY) and system cost (*R*^2^ of 0.992–1.000) under diverse assumptions for biomanufacturing facility design decisions, technological performance, and contextual parameters (32 distinct biomanufacturing feedstock-to-product configurations simulated under uncertainty and across 240,000 combinations of fermentation TRY using BioSTEAM), and validated it using TEA results from external studies that explored six distinct biomanufacturing feedstock-to-product configurations using Aspen Plus and SuperPro Designer (*R*^2^ of 0.954–1.000 on results from external studies). Further, we simulated 24 biomanufacturing configurations under uncertainty at each of 25 yield-titer combinations for a total of 600,000 Monte Carlo simulations to evaluate how uncertain design, technological, and contextual parameters influence the relationship between fermentation TRY and system cost. To the best of the authors’ knowledge, no previous work has suggested a mathematical equation that can capture the relationship between fermentation TRY and system cost to this degree of robustness across the vast design and thermodynamic simulation landscapes evaluated in this work, nor has it performed rigorous uncertainty analyses to generate widely applicable insights into the process drivers of this relationship as presented in this work.

In exploring the physical interpretations of the coefficients of the proposed mathematical equation (Eq. 12) by simulating biomanufacturing facilities under uncertainty (600,000 Monte Carlo simulations), we revealed key drivers and non-drivers of the relationship between fermentation TRY and system cost. By interpreting design, technological, and contextual drivers of the facility’s yield-dependent burden coefficient ${k}_{Y}$, we found that while the fermentation regime (neutral vs. low pH) and the inclusion of catalytic upgrading influenced system cost, they did not substantially alter how fermentation TRY influenced system cost. Similarly, costs associated with the separation process equipment farther downstream (specifically, downstream of separation equipment that removed water from the fermentation broth to achieve a target concentration) influenced system cost, but did not substantially alter the relationship between fermentation performance and system cost. Further, by interpreting the facility’s TRY-invariant burden coefficient ${k}_{0}$, we found the choice of feedstock and the energy content of the waste fractions of the feedstock and fermentation broth substantially affected the influence of fermentation yield on system cost—more expensive feedstocks and lower total recoverable energy in wastes (by WWRR and CHP) amplified this influence. Finally, by interpreting the facility’s broth volumetric flow-dependent burden coefficient ${k}_{V}$, we found costs associated with the separation process equipment immediately downstream of fermentation (specifically, including and upstream of equipment that first removed water from the fermentation broth to achieve a target concentration rather than a target amount of water removal) substantially affected the influence of titer on system cost—more expensive separations including and upstream of target concentration-based water removal amplified this influence. Similarly, an increase in costs associated with non-sugar, fixed-concentration nutrient additives to the fermentation medium (e.g., to satisfy nitrogen and phosphorus requirements) increased the influence of titer on system cost.

The analytical framework developed in this work—performing local sensitivity analyses across vast theoretical technological spaces to uncover underlying equations, followed by rigorous uncertainty and global sensitivity analyses to deepen an understanding of the relationships described by those equations—may be used to reveal broadly applicable recommendations to advance new process design paradigms that maximize the benefit from anticipated technological improvements. For example, with several alternative process flowsheets that result in similar minimum product selling prices, it may be prudent for a process design practitioner to compare values for ${k}_{V}$ across flowsheets and select the flowsheet associated with the larger ${k}_{V}$ term (e.g., due to costlier separations including and prior to initial water removal, or costlier fermentation nutrient media components) if future improvements to fermentation titer are anticipated. Similarly, it may be prudent to select the flowsheet associated with the larger ${k}_{0}$ term (e.g., from costlier feedstocks, or from lower recoverable energy in the waste fractions diverted to wastewater resource recovery) if future improvements to fermentation yield are anticipated. Follow-up studies could also evaluate the sensitivity of the coefficient of determination (R^2^) to uncertain parameters to reveal key zones in the design, technological, and contextual landscape in which the developed equation does not capture the relationship between fermentation TRY and system cost as robustly, thereby guiding further parametrization.

Further, the developed equation is explicit in MPSP and could therefore be used in high-throughput paradigms for early-stage research to estimate MPSP analytically for low computational cost across vast fermentation TRY spaces; i.e., by simply substituting the TRY values of interest into the equation without requiring hundreds of thousands of computationally costly chemical process simulations. For example, the equation may be leveraged to design holistic objective functions or guidance metrics (e.g., ${{{{\rm{RI}}}}}_{{{{\rm{MPSP}}}}}$; Eq. 18) for platforms that aim to engineer optimal metabolic pathways in microbes through automated prototyping^27–30^, especially alongside potential future efforts to develop artificial intelligence-based approaches to predict equation coefficient values from feedstock, product, and process specifications. In addition, using more detailed process models for wastewater resource recovery processes (e.g., anaerobic digestion) that include the influence of potential inhibitory compounds on technological performance may enable using the developed framework to navigate trade-offs in the design of wastewater resource recovery processes in terms of systems-scale sustainability at the current state-of-technology and under potential future fermentation advancements. Finally, future work to explore suitably modifying the proposed mathematical equation to capture how the biomanufacturing facility’s environmental impacts (estimated via life cycle assessment, LCA) are influenced by fermentation performance will be critical to navigating economic and environmental trade-offs. Overall, the conclusions from this study illustrate how agile and robust system analyses can address critical barriers to generating widely applicable insight, enhance mechanistic understanding, elucidate salient trends, and prioritize future research and development needs.

## Methods

### System description

#### Pretreatment, saccharification, fermentation, separation, and catalytic upgrading processes

The biomanufacturing facilities in this study are comprised of four main (inside battery limits) processes (feedstock pretreatment and saccharification, fermentation, separation, and catalytic upgrading) with outside-battery wastewater resource recovery, combined heat and power generation, and miscellaneous facilities. An overview of biomanufacturing facility processes and the configurations of the 32 biomanufacturing facilities evaluated in this study is provided in Fig. 1 and in Supplementary Data 1).

All process models used in this work were developed in previous studies: namely, for feedstock pretreatment and saccharification (corn dry grind process^21^, sugarcane juicing and clarification^21^, and corn stover dilute acid pretreatment and enzymatic saccharification^21^); for fermentation of sugars (to 3HP^18^, TAL^16^, and succinic acid^10^); for separation of fermentation products (3HP^12,22^, TAL^16^, and succinic acid^10^); and for catalytic upgrading of fermentation products (upgrading 3HP to acrylic acid^12^ and TAL to potassium sorbate^25^). The feedstock capacity (1220 metric ton·d^−1^ for dextrose monohydrate, 1370 metric ton·d^−1^ for corn, 11500 metric ton·d^−1^ for sugarcane, and 2060 metric ton·d^−1^ for corn stover) and operating time (330 d·y^−1^ for dextrose monohydrate, corn, and corn stover, and 180 d·y^−1^ for sugarcane) assumed for each biomanufacturing facility depended on the choice of feedstock and were chosen to be consistent with previous TEAs^12,13,25,31,32^ (full lists of baseline values, uncertainty distributions, and literature references for all parameters included in the uncertainty analyses are provided in Supplementary Data 3).

In all fermentation processes modeled, the juice (from sugarcane) or slurry (from corn or corn stover) undergoes a two-step scheme involving multiple-effect evaporation to remove 80% of the water initially present, followed by dilution as needed to achieve the necessary concentration of sugars, depending on the fermentation titer and yield evaluated. The sequentially evaporated and diluted juice or slurry is sent to fermentation with *Issatchenkia orientalis* (to produce 3HP or succinic acid) or *Yarrowia lipolytica* (to produce TAL). Note that, to be consistent with chemical engineering literature, the term *fermentation* is used here to mean microbial conversion (including aerobic conversion) of a substrate to a specific product in a bioreactor.

#### Facilities

Facilities in the biomanufacturing facility include a boiler (for on-site heat utility production), turbogenerator (for on-site electricity production), a cooling tower and chilled water system (for on-site cooling utility production), wastewater resource recovery (for succinic acid, a conventional treatment process that included anaerobic basins for biogas production was used; for 3HP and TAL biomanufacturing facilities, a high-rate process scheme that included internal circulation reactors and anaerobic membrane bioreactors for biogas production was used)^33^, heat exchanger network (HXN, for heat integration to minimize heating and cooling utility demands), process water center (for water reuse), and other auxiliary units for storage, air distribution, and clean-in-place. These facilities were modeled to be consistent with previous TEA studies^10,12,16,18,25^.

#### Open-source system model

All biomanufacturing facilities were designed, simulated, and evaluated using BioSTEAM^21,34^, and the thermodynamic package utilized was Thermosteam^24,35^. Briefly, influent and effluent streams of each unit are simulated in BioSTEAM and coupled with operating parameters and equipment cost algorithms for unit design and cost calculations. Further descriptions of major processes and units are included in the original studies for each process model described in *System Description*. All Python scripts for BioSTEAM and the 32 biomanufacturing facilities evaluated in this work (including biomanufacturing facility setup and system analyses) as well as system reports (including detailed process flowsheets, stream composition and cost tables, unit design specifications, and utilities for the baseline simulations) are available in the online repositories^34,36–39^.

### System analyses under uncertainty

#### Techno-economic analysis (TEA) and life cycle assessment (LCA)

We performed TEA and LCA following established procedures for bioproducts and biofuels^10–15,17^. Briefly, TEA was executed using BioSTEAM’s discounted cash flow rate of return analysis to calculate the minimum product selling price (MPSP, $·kg^−1^) of the final product (acrylic acid for 3HP biomanufacturing facilities; TAL or potassium sorbate for TAL biomanufacturing facilities; succinic acid for succinic acid biomanufacturing facilities) to achieve a net present value of zero with a targeted annual internal rate of return (10% for baseline simulations; baseline values and distributions for all parameters included in the uncertainty analyses are detailed in Supplementary Data 3). The TEA year was set to 2019 (all costs are reported in 2019 US$). Key construction (e.g., warehouse, site development), fixed operating (e.g., labor burden, property insurance), and financial (e.g., depreciation, taxes) parameters followed assumptions in previous studies^40,41^. Breakdowns of the estimated revenues, capital and operating expenditures, as well as additional details on the design, utility requirements, purchase costs, and installed equipment costs for baseline simulations for all biomanufacturing facilities can be found online^36^.

#### Uncertainty and sensitivity analyses

The design, simulation, and evaluation of biomanufacturing facilities over entire theoretical fermentation spaces were performed using models from previous studies^10,12,16,18^. For each of the 32 biomanufacturing facilities evaluated in this work (Fig. 1), 2500 potential combinations of fermentation yield and titer were evaluated at 3 alternative values of fermentation productivity (20%, 100%, and 500% of each of the reported baseline productivity values of 0.12 g-*fp*·L^−1^·h^−1^ for TAL, 0.28 g-*fp*·L^−1^·h^−1^ for 3HP, and 0.66 g-*fp*·L^−1^·h^−1^ for succinic acid) for a total of 240,000 simulations^10,16,18^. For fermentation yield, the lower bounds for evaluation were set to ~5% of the theoretical maximum yields to ensure non-zero product recovery (the theoretical maximum yields were 0.467 g∙g^−1^ glucose-eq. for TAL, 1.000 g∙g^−1^ glucose-eq. for 3HP, and 1.31 g∙g^−1^ glucose-eq. for succinic acid) and the upper bounds were each set to the maximum value at which: (i) the total glucose conversion (alongside other reactions for microbial cell mass formation, co-product formation, and respiration, based on their reported baseline yields^10,16,18^.) did not exceed 100%; and (ii) the fermentation product yield did not exceed the theoretical maximum yield. For fermentation titer, the upper bounds for evaluation were set to 100 g∙L^−1^ for TAL (note that the utilized process models adequately characterize the implications of the low solubility of TAL in water^16^), 200 g∙L^−1^ for 3HP, and 120 g∙L^−1^ for succinic acid based on the bounds used for evaluation in previous studies^10,16,18^.

Further, for 24 biomanufacturing facilities (all biomanufacturing facilities producing TAL, potassium sorbate, or acrylic acid), uncertainty analyses were conducted at each of 25 fermentation yield-titer combinations using Monte Carlo simulations with Latin Hypercube Sampling (1000 simulations at each yield-titer combination) for 31 uncertain parameters (for biomanufacturing facilities producing acrylic acid via 3HP), 27 uncertain parameters (for TAL biomanufacturing facilities without catalytic upgrading), 49 uncertain parameters (for TAL biomanufacturing facilities with catalytic upgrading to potassium sorbate), or 25 uncertain parameters (for succinic acid biomanufacturing facilities), for a total of 600,000 Monte Carlo simulations. Next, for each Monte Carlo simulation, Eq. 11 was fit to values for MPSP obtained over 25 yield-titer combinations, resulting in a distribution of values for each of the empirical coefficients ${k}_{Y}$, ${k}_{0}$, ${k}_{V}$, and ${k}_{T}$ in Eq. 11 for each biomanufacturing facility. The sensitivity of empirical coefficients ${k}_{Y}$, ${k}_{0}$, ${k}_{V}$, and ${k}_{T}$ to all uncertain inputs was determined via Spearman’s rank order correlation coefficients (Spearman’s *ρ*), and only parameters to which the empirical coefficients were most sensitive (i.e., |Spearman’s *ρ*| ≥ 0.10 and *p* value < 0.05) were used to identify key drivers influencing the empirical coefficients ${k}_{Y}$, ${k}_{0}$, ${k}_{V}$, and ${k}_{T}$ in the discussion. Finally, for each of the 32 biomanufacturing facilities evaluated in this work, we computed partial derivatives of Eq. 4 with respect to yield and titer and evaluated the ratio of the partial derivatives across 2500 combinations of fermentation yield and titer values at the reported baseline productivities. Files with comprehensive results of all analyses are available online^36^.

### Statistics and reproducibility

No statistical method was used to predetermine sample size. At each of 25 yield-titer combinations for 32 biomanufacturing facilities, 1000 Monte Carlo simulations (sampled by Latin hypercube sampling) were performed, which were sufficient to result in *p* values < 0.05 in the associated global sensitivity analyses to quantify correlations between equation coefficients and all uncertain parameters discussed in this work. No data were excluded from the analyses. The experiments were not randomized, and the investigators were not blinded to allocation during experiments and outcome assessment.

### Reporting summary

Further information on research design is available in the Nature Portfolio Reporting Summary linked to this article.

## Supplementary information

Supplementary Information

Description of Additional Supplementary Files

Supplementary Data 1

Supplementary Data 2

Supplementary Data 3

Supplementary Data 4

Supplementary Data 5

Supplementary Data 6

Supplementary Data 7

Reporting Summary

Transparent Peer Review file

## Source data

Source Data

## Data Availability

Source data are provided with this paper. All results (including plots and raw data) are available at *sarangbhagwat: Modules to generate widely applicable fermentation insights, 2025* (https://github.com/sarangbhagwat/fermentation_insights/tree/main), *BioSTEAMDevelopmentGroup: Triacetic acid lactone biorefineries, 2025* (https://github.com/BioSTEAMDevelopmentGroup/Bioindustrial-Park/tree/master/biorefineries/TAL), *BioSTEAMDevelopmentGroup: 3-hydroxypropionic acid biorefineries, 2025* (https://github.com/BioSTEAMDevelopmentGroup/Bioindustrial-Park/tree/master/biorefineries/HP), and *BioSTEAMDevelopmentGroup: Succinic acid biorefineries, 2025* (https://github.com/BioSTEAMDevelopmentGroup/Bioindustrial-Park/tree/master/biorefineries/succinic). Source data are provided with this paper.

## Code availability

The software scripts used to generate all results (including plots and raw data) are available open-source at *sarangbhagwat: Modules to generate widely applicable fermentation insights, 2025* (https://github.com/sarangbhagwat/fermentation_insights/tree/main), *BioSTEAMDevelopmentGroup: Triacetic acid lactone biorefineries, 2025* (https://github.com/BioSTEAMDevelopmentGroup/Bioindustrial-Park/tree/master/biorefineries/TAL), *BioSTEAMDevelopmentGroup: 3-hydroxypropionic acid biorefineries, 2025* (https://github.com/BioSTEAMDevelopmentGroup/Bioindustrial-Park/tree/master/biorefineries/HP), and *BioSTEAMDevelopmentGroup: Succinic acid biorefineries, 2025* (https://github.com/BioSTEAMDevelopmentGroup/Bioindustrial-Park/tree/master/biorefineries/succinic). BioSTEAM (version 2.46.1; https://github.com/BioSTEAMDevelopmentGroup/Bioindustrial-Park/tree/49f2e618a81b2fc1a6aae14fdd588e838b1e777f) was used to design and simulate biomanufacturing facilities. All dependencies are listed in the README file in https://github.com/sarangbhagwat/fermentation_insights/tree/main.
