# Supplementary material for: A unifying equation for fermentation sustainability across the titer-rate-yield landscape
Source: Nat Commun. 2026 Jul 10;17:8655. doi: 10.1038/s41467-026-75285-1 (PMC13490486; doi:10.1038/s41467-026-75285-1)
Supplement: Supplementary file 1 [file 41467_2026_75285_MOESM10_ESM.pdf]

nature portfolio

Corresponding author(s): Jeremy S. Guest and Sarang S. Bhagwat

Last updated by author(s): 12/18/2025

Reporting Summary

Nature Portfolio wishes to improve the reproducibility of the work that we publish. This form provides structure for consistency and transparency in reporting. For further information on Nature Portfolio policies, see our Editorial Policies and the Editorial Policy Checklist.

Statistics

For all statistical analyses, confirm that the following items are present in the figure legend, table legend, main text, or Methods section.

n/a	Confirmed	
☐	☒	The exact sample size (*n*) for each experimental group/condition, given as a discrete number and unit of measurement
☒	☐	A statement on whether measurements were taken from distinct samples or whether the same sample was measured repeatedly
☐	☒	The statistical test(s) used AND whether they are one- or two-sided<br>*Only common tests should be described solely by name; describe more complex techniques in the Methods section.*
☒	☐	A description of all covariates tested
☐	☒	A description of any assumptions or corrections, such as tests of normality and adjustment for multiple comparisons
☐	☒	A full description of the statistical parameters including central tendency (e.g. means) or other basic estimates (e.g. regression coefficient) AND variation (e.g. standard deviation) or associated estimates of uncertainty (e.g. confidence intervals)
☐	☒	For null hypothesis testing, the test statistic (e.g. *F*, *t*, *r*) with confidence intervals, effect sizes, degrees of freedom and *P* value noted<br>*Give P values as exact values whenever suitable.*
☒	☐	For Bayesian analysis, information on the choice of priors and Markov chain Monte Carlo settings
☒	☐	For hierarchical and complex designs, identification of the appropriate level for tests and full reporting of outcomes
☐	☒	Estimates of effect sizes (e.g. Cohen's *d*, Pearson's *r*), indicating how they were calculated

Our web collection on statistics for biologists contains articles on many of the points above.

Software and code

Policy information about availability of computer code

Data collection	All 32 biomanufacturing facility configurations evaluated are available at https://github.com/BioSTEAMDevelopmentGroup/Bioindustrial-Park. All analyses were conducted using code available at https://github.com/sarangbhagwat/fermentation_insights/tree/main. The package BioSTEAM v 2.46.1 was used to design and simulate biomanufacturing facilities (https://github.com/BioSTEAMDevelopmentGroup/Bioindustrial-Park/tree/49f2e618a81b2fc1a6aae14fdd588e838b1e777f). All dependencies are listed in the README file in https://github.com/sarangbhagwat/fermentation_insights/tree/main. All code and data used and developed for this work are open-source.
Data analysis	All 32 biomanufacturing facility configurations evaluated are available at https://github.com/BioSTEAMDevelopmentGroup/Bioindustrial-Park. All analyses were conducted using code available at https://github.com/sarangbhagwat/fermentation_insights/tree/main. The package BioSTEAM v 2.46.1 was used to design and simulate biomanufacturing facilities (https://github.com/BioSTEAMDevelopmentGroup/Bioindustrial-Park/tree/49f2e618a81b2fc1a6aae14fdd588e838b1e777f). All dependencies are listed in the README file in https://github.com/sarangbhagwat/fermentation_insights/tree/main. All code and data used and developed for this work are open-source.

For manuscripts utilizing custom algorithms or software that are central to the research but not yet described in published literature, software must be made available to editors and reviewers. We strongly encourage code deposition in a community repository (e.g. GitHub). See the Nature Portfolio guidelines for submitting code & software for further information.

Data

Policy information about availability of data

All manuscripts must include a data availability statement. This statement should provide the following information, where applicable:

- Accession codes, unique identifiers, or web links for publicly available datasets
- A description of any restrictions on data availability
- For clinical datasets or third party data, please ensure that the statement adheres to our policy

All data, figures, and code to generate the same are available open-source at https://github.com/sarangbhagwat/fermentation_insights/tree/main.

Research involving human participants, their data, or biological material

Policy information about studies with human participants or human data. See also policy information about sex, gender (identity/presentation), and sexual orientation and race, ethnicity and racism.

Reporting on sex and gender	No human participants were involved in the data collection for this study. All data collection was purely computational.
Reporting on race, ethnicity, or other socially relevant groupings	No human participants were involved in the data collection for this study. All data collection was purely computational.
Population characteristics	No human participants were involved in the data collection for this study. All data collection was purely computational.
Recruitment	No human participants were involved in the data collection for this study. All data collection was purely computational.
Ethics oversight	No human participants were involved in the data collection for this study. All data collection was purely computational.

Note that full information on the approval of the study protocol must also be provided in the manuscript.

Field-specific reporting

Please select the one below that is the best fit for your research. If you are not sure, read the appropriate sections before making your selection.

☒ Life sciences ☐ Behavioural & social sciences ☐ Ecological, evolutionary & environmental sciences

For a reference copy of the document with all sections, see nature.com/documents/nr-reporting-summary-flat.pdf

Life sciences study design

All studies must disclose on these points even when the disclosure is negative.

Sample size	All data collection in this study was purely computational. In total, 240,000 simulations across yield-titer-productivity (7500 yield-titer-productivity combinations for each of 32 biomanufacturing facility configurations) and 600,000 Monte Carlo simulations (2000 Monte Carlo simulations at each of 25 yield-titer combinations for each of 24 biomanufacturing facilitiy configurations) were performed.
Data exclusions	No collected data were excluded from this study.
Replication	All installation instructions to reproduce the results of this study are detailed in ttps://github.com/sarangbhagwat/fermentation_insights/tree/main and https://github.com/BioSTEAMDevelopmentGroup/Bioindustrial-Park.
Randomization	Randomization is not relevant to our study, as all evaluated configurations were designed by the first author.
Blinding	Blinding is not relevant to our study, as all evaluated configurations were designed by the first author.

Reporting for specific materials, systems and methods

We require information from authors about some types of materials, experimental systems and methods used in many studies. Here, indicate whether each material, system or method listed is relevant to your study. If you are not sure if a list item applies to your research, read the appropriate section before selecting a response.

Materials & experimental systems

n/a	Involved in the study
☒	☐ Antibodies
☒	☐ Eukaryotic cell lines
☒	☐ Palaeontology and archaeology
☒	☐ Animals and other organisms
☒	☐ Clinical data
☒	☐ Dual use research of concern
☒	☐ Plants

Methods

n/a	Involved in the study
☒	☐ ChIP-seq
☒	☐ Flow cytometry
☒	☐ MRI-based neuroimaging

Plants

Seed stocks	No plant specimens were involved in this study.
Novel plant genotypes	No plant specimens were involved in this study.
Authentication	No plant specimens were involved in this study.
