# Supplementary material for: A unifying equation for fermentation sustainability across the titer-rate-yield landscape
Source: Nat Commun. 2026 Jul 10;17:8655. doi: 10.1038/s41467-026-75285-1 (PMC13490486; doi:10.1038/s41467-026-75285-1)
Supplement: Supplementary file 2 [file 41467_2026_75285_MOESM11_ESM.pdf]

nature portfolio

Peer Review File

A Unifying Equation for Fermentation Sustainability across the Titer-Rate-Yield Landscape

Corresponding Author: Professor Jeremy Guest

This file contains all reviewer reports in order by version, followed by all author rebuttals in order by version.

Version 0:

Reviewer comments:

Reviewer #1

(Remarks to the Author)
This manuscript proposes a four-parameter equation (MPSP = a + b/yield + c/titer + d/(yield×titer)) to predict how fermentation titer and yield influence minimum product selling price in biomanufacturing. The authors validate this relationship using over 240,000 techno-economic simulations across 32 facility configurations encompassing three products (TAL, 3HP, succinic acid) and four feedstocks. The reported R^2 values (0.992–1.000 internally; 0.954–1.000 for external validation) are impressive.

The work represents a valuable contribution analogous to foundational engineering relationships such as the economies-of-scale power law. While the mathematical form is intuitive, the true contribution lies in the rigorous empirical validation demonstrating that this simple equation reliably captures cost behavior across diverse biomanufacturing systems. The use of the open-source BioSTEAM platform ensures transparency and facilitates community adoption.

Major Comments
1. Rate independence requires clarification. The authors acknowledge that fermentation rate (productivity) primarily affects capital costs but exclude it from the final equation. Since productivity directly impacts equipment sizing and capital utilization, which are key factors in industrial economics, the authors should clarify the conditions under which rate independence holds, discuss the range of productivities over which the equation remains valid, and consider whether a rate-dependent term might be needed for capital-intensive processes.
2. Sustainability framing versus economic scope. The title emphasizes “Fermentation Sustainability,” yet the analysis focuses exclusively on economics (MPSP). While economic viability is necessary for sustainable biomanufacturing, sustainability encompasses environmental impacts (LCA), resource efficiency, and social factors. The authors mention carbon intensity briefly but do not integrate environmental metrics with their equation. I recommend either modifying the title to better reflect the economic focus (for example, “Fermentation Economics” or “Fermentation Viability”), or expanding the discussion to explicitly address how MPSP relates to broader sustainability metrics, with appropriate caveats about the scope of the current analysis.
3. Independence of external validation. Many of the external validation studies appear to have been conducted using the same BioSTEAM platform, some by overlapping author groups. To strengthen confidence in generalizability, the authors should clearly indicate which external studies used independent simulation frameworks, discuss whether the equation’s success might partly reflect consistency in BioSTEAM’s embedded modeling assumptions, and consider validation against actual industrial cost data if available.

Minor Comments
4. Limitations section. The manuscript would benefit from a brief dedicated section discussing the current product scope (commodity chemicals) and untested extensions (fine chemicals, pharmaceuticals, proteins), the absence of industrial-scale cost validation, and assumptions embedded in the BioSTEAM models.
5. Coefficient interpretation. The Discussion could be strengthened by providing more physical interpretation of the fitted coefficients (a, b, c, d) and how their relative magnitudes reveal underlying cost drivers across different facility configurations.

(Remarks on code availability)

Reviewer #2

(Remarks to the Author)
This work is well designed and performed, which is an important topics for economic biomanufacturing but neglected due to the lack of data and methodology. Although the manuscript is well-written, there are still typos with long paragraphs, which needs to be improved. My suggestions are to take the theoretical yield of the target product of the producing microorganism into consideration for the uncertainty study, to consider the situations of anaerobic and aerobic fermentation, and to include other products that are currently for commercial production besides the three ones in this study such as ethanol, 1,3-PDO, 1,4-BDO, and amino acids as well as single cell protein and PHA. Will the equation fit diverse products of different fermentation conditions?

(Remarks on code availability)

Reviewer #3

(Remarks to the Author)
Bhagwat et al. provide a comprehensive analysis to demonstrate that complex process and economic models can be easily modeled using a small number of parameters. The approach is thorough, using a complex process design with 32 unique arrangements of feedstocks, processes, and products. Their approach is supported with Monte Carlo simulations and sensitivity analyses. The proposed approach for simplifying complex analyses offers a unique perspective, but additional clarification and validation is needed. I recommend the manuscript for major revision before consideration for publication.

The thesis presented by the authors is that a complex process model could be fit by a simple linear model that relies on a small number of parameters. But wouldn't a multivariate linear regression produce the same results, identifying the same key factors that have the most significant influence on MPSP (e.g., MPSP = a + b*X_1 + c*X_2 + d*X_1*X_2)? Can the authors better explain how their approach differs from existing linear regression methods? Would the paper be strengthened if the authors provided a direct comparison of their results against the results from a linear regression approach?

The explanation of the coefficients is not easily understood as presented, when they could be summarized as the sale price of the fermentation product, the cost of feedstock, the cost of processing the fermentation broth, and a complex variable that incorporates cost of processing the feedstock and broth. The proposed equation XI is just a simple cost formula that is dependent on material pricing and is independent of the inputs relevant for capital and operational expenditures. While the authors present their approach as novel, it appears to align with established economic principles (cost of product = cost of raw materials + cost of processing). Clarifying how this work extends or differentiates from existing frameworks would strengthen the contribution. Are the authors unintentionally overcomplicating a simple and well-known approach for process costing?

The utility of any model is the ability to predict outcomes. Can the authors provide any context about the predictive capability of their process models? How do their correlated outcomes compare with any physical systems? Are there other specific advantages of using the proposed reduced order model over traditional process and cost models? As mentioned above, does the outcome of the coefficient values elucidate any key constraints to consider for the process design or economic drivers for a particular process?

Additional comments are provided below to improve the clarity of the manuscript.
Figure 1: Have you considered alternative visualizations to better discern the goodness of fit and where the model may fail to accurately match results? For example, what about a surface plot that shows % difference between the detailed model and the reduced-order fit model? Doing so would identify 'hotspots' where the reduced order model fails to accurately represent details. Figure S5 is a good example.

R-squared values in figure S2 can be difficult to read

Line 316: What is a boiler prism? I don't think this is common terminology that will be easily understood, I don't really know what it is.

Figure references appear to be incorrect.

Examples:

Line 142-143: "32 right-hand side panels in Figure 1 and Figures S2 and S3 in the SI"
This appears to refer to Figures S1 and S2, although Figure S3 also contains 32 right-hand side panels

S1.1 Fermentation Processes, paragraph 3: "This manifests as an infeasible region in the upper-left portion of the fermentation titer-yield space (represented by white regions in Figures 1, S2, S3, S4, S6, and S7)."
Figure S4 is a Sankey diagram, Figure S7 is a black-and-white contour plot, and Figure S1 is similar to Figure S2 but is not included in the list.

In general, the writing should be reviewed for brevity and clarity.

Examples:

Line 120:
"… and two catalytic upgrading processes (3HP [...]; TAL [...]; all biomanufacturing facility configurations are shown in Figure 5 [...])."

The final phrase should not be included in the list of catalytic upgrading processes. Instead, this could be "… and two catalytic upgrading processes (3HP [...]; TAL [...]). All biomanufacturing facility configurations are shown in Figure 5 [...]."

Line 534:
"[...] four main (inside battery limits) processes (feedstock pretreatment [...] and catalytic upgrading) with outside-battery wastewater resource recovery, [...] and miscellaneous facilities (an overview of biomanufacturing facility processes [...] is provided in Figure 5 and in Table S1 in the SI)."

This sentence structure implies that the final parentheses is clarifying the term "miscellaneous facilities". To remove ambiguity, this could be written as "… and miscellaneous facilities. An overview of biomanufacturing facility processes [...] is provided in Figure 5 and in Table S1 in the SI."

Line 554-569:

The following adjacent sentences appear to repeat the same information:

"Models for all process alternatives were from previous studies; specifically: (i) corn dry grind; (ii) sugarcane juicing and clarification; [...] (xii) 3HP catalytic upgrading to acrylic acid. 13 All listed models are available open-source in the online repositories.30–33"

"All process models [...] were developed in previous studies: namely, for feedstock pretreatment and saccharification (corn dry grind process, sugarcane juicing and clarification, [...]; and for catalytic upgrading of fermentation products (upgrading 3HP to acrylic acid [...])."
These sentences could be combined. If they convey different information, the differences should be clarified.

Figure 4 caption, lines 451 – 457:
"Rows represent biomanufacturing facilities producing TAL as the main product (TAL), biomanufacturing facilities producing potassium sorbate via TAL [...], and biomanufacturing facilities producing succinic acid (succinic acid) under low-pH (low-pH) and neutral fermentation regimes (neutral). Columns represent biomanufacturing facilities accepting dextrose monohydrate 458 (glucose), [...], and corn (corn) as the feedstock (all [...] described in Figure 5)." (95 words)

This could be reduced to
"Rows represent biomanufacturing facilities producing TAL, potassium sorbate via TAL, acrylic acid via 3HP under low-pH and neutral fermentation regimes and with separation process A or B, and succinic acid under low-pH and neutral fermentation regimes. Columns represent biomanufacturing facilities accepting glucose, sugarcane, corn stover, and corn as the feedstock." (50 words)

In lines 618 and 629, providing values taken from literature or github could help readers understand the assumptions used in the analysis. This was done in lines 149-152.

(Remarks on code availability)
The code used in the project has been made publicly available on GitHub. The authors have also included thorough documentation of how to use the code and implement the analysis independently.

Reviewer #4

(Remarks to the Author)
I co-reviewed this manuscript with one of the reviewers who provided the listed reports. This is part of the Nature Communications initiative to facilitate training in peer review and to provide appropriate recognition for Early Career Researchers who co-review manuscripts.

(Remarks on code availability)

Version 1:

Reviewer comments:

Reviewer #1

(Remarks to the Author)

The authors have addressed this reviewer's comments.

(Remarks on code availability)
The repository looks good. The README could be improved by including a section for readers that are not familiar with python, github, and/or biosteam. Several technical terms, like repository cloning, could benefit from links to introduction tutorials.

Reviewer #2

(Remarks to the Author)
The authors addressed my comments and significantly improved the manuscript. I recommend it for publishing in NC.

(Remarks on code availability)
The code is available with file for installation.

Reviewer #3

(Remarks to the Author)
Bhagwat et al. have provided a thorough revision to their manuscript. After review of their revised manuscript and rebuttal letter, the authors have reasonably addressed reviewer questions and concerns. I thank the authors for their detailed responses and thorough revision. Overall, the manuscript is acceptable for publication.

As noted in the first review, some text does not perfectly align with the content of certain figures. New instances are noted below for the authors to review before finalizing the publication:

Line 254: "*r* does not change substantially with fermentation yield, titer, or productivity (Figures S4 and S7B in the SI)."

Line 375: "the total volume of the fermentation reactors were both expected to increase linearly with the ratio of fermentation yield to titer at a constant productivity (Figure S4C in the SI)"

Line 401: "none of the biomanufacturing facility flows associated with these parameters were expected to increase linearly with the ratio of fermentation yield to titer (Figure S4 in the SI)"

(Remarks on code availability)
Code for new figures appears to be present and functional.

Reviewer #4

(Remarks to the Author)
I co-reviewed this manuscript with one of the reviewers who provided the listed reports. This is part of the Nature Communications initiative to facilitate training in peer review and to provide appropriate recognition for Early Career Researchers who co-review manuscripts.

(Remarks on code availability)

Open Access This Peer Review File is licensed under a Creative Commons Attribution 4.0 International License, which permits use, sharing, adaptation, distribution and reproduction in any medium or format, as long as you give appropriate credit to the original author(s) and the source, provide a link to the Creative Commons license, and indicate if changes were made.
In cases where reviewers are anonymous, credit should be given to 'Anonymous Referee' and the source.
The images or other third party material in this Peer Review File are included in the article's Creative Commons license, unless indicated otherwise in a credit line to the material. If material is not included in the article's Creative Commons license and your intended use is not permitted by statutory regulation or exceeds the permitted use, you will need to obtain permission directly from the copyright holder.
To view a copy of this license, visit https://creativecommons.org/licenses/by/4.0/

A Unifying Equation for Fermentation Sustainability across the Titer-Rate-Yield Landscape

Responses to Reviewer Comments

REVIEWER 1

1.1. General Comments:

This manuscript proposes a four-parameter equation (MPSP = a + b/yield + c/titer + d/(yield×titer)) to predict how fermentation titer and yield influence minimum product selling price in biomanufacturing. The authors validate this relationship using over 240,000 techno-economic simulations across 32 facility configurations encompassing three products (TAL, 3HP, succinic acid) and four feedstocks. The reported R^2 values (0.992–1.000 internally; 0.954–1.000 for external validation) are impressive.

The work represents a valuable contribution analogous to foundational engineering relationships such as the economies-of-scale power law. While the mathematical form is intuitive, the true contribution lies in the rigorous empirical validation demonstrating that this simple equation reliably captures cost behavior across diverse biomanufacturing systems. The use of the open-source BioSTEAM platform ensures transparency and facilitates community adoption.

Response:
We thank the Reviewer for their positive assessment of the work and for their constructive comments. We have addressed the comments raised by the Reviewer and the responses are detailed below.

1.2. Specific Comments:
1.2.1. *Rate independence requires clarification. The authors acknowledge that fermentation rate (productivity) primarily affects capital costs but exclude it from the final equation. Since productivity directly impacts equipment sizing and capital utilization, which are key factors in industrial economics, the authors should clarify the conditions under which rate independence holds, discuss the range of productivities over which the equation remains valid, and consider whether a rate-dependent term might be needed for capital-intensive processes.*

Response:
The reviewer correctly identifies that for a given feedstock capacity and fermentation yield and titer, the rate (productivity) influences the fermentation time, which dictates the required bioreactor size and therefore primarily its equipment purchase and installation cost. To clarify, we do not claim rate-independence of system costs, but rather provide two forms of the unifying equation:

(1) MPSP vs. fermentation yield and titer at a *constant* productivity:

$$MPSP = \frac{k_Y + \frac{k_0}{yield} + \frac{k_V}{titer} + \frac{k_T}{yield*titer}}{r} \quad \text{(Equation XI)}$$

Results from fitting simulations to **Equation XI** are presented in **Figures 1** and **3** in the Manuscript and in **Figures S1** and **S2** in the SI. (Note: We have changed the symbols for the four coefficients from a', b', c', and d' to instead by k_Y, k_0, k_V, and k_T, respectively. This change was implemented to help address Comment 1.2.5.)

(2) MPSP vs. fermentation yield, titer, and productivity:
We substitute **Equations XIII**, **XIV**, **XV**, and **XVI** (shown below in the updated text) for coefficients in **Equation XI**. Results used to derive these productivity-dependent coefficient relationships are presented in **Figure S6** in the **Supplementary Information**.

However, we agree with the reviewer that this could be better highlighted and discussed in the manuscript. We have accordingly revised the **Results** to include the full form of the resulting productivity-dependent equation for clarity and expand the discussion. In addition to the already present **Figure S6** (added a missing color bar and updated the caption for clarity), we also provide new supporting data in **Figure S11** in the **Supplementary Information**.

Revision:

Results:

[Lines 432–449]

Physical interpretation of equation coefficients and cost drivers under uncertainty

[…]. At a fixed feedstock capacity (as with each biomanufacturing configuration in the baseline case), when fermentation productivity changes at a fixed titer and yield, only fermentation time changes among all parameters described in **Equations I**, **II**, and **III**, limiting the influence of productivity on MPSP to costs associated with the purchase and installation of fermentation bioreactors and the power utility demanded for mixing bioreactor contents (**Figure S11** in the SI). We found the following equations described the influence of fermentation productivity on the coefficients $k_Y, k_0, k_V, and\ k_T$ (multiplying coefficient values with their associated productivity values resulted in straight lines not crossing the origin when plotted against productivity for biomanufacturing facilities producing potassium sorbate via TAL from sugarcane; **Figure S6** in the SI):

$$k_Y = a_1 + \frac{a_2}{productivity} \quad \text{(Equation XIII)}$$

$$k_0 = b_1 + \frac{b_2}{productivity} \quad \text{(Equation XIV)}$$

$$k_V = c_1 + \frac{c_2}{productivity} \quad \text{(Equation XV)}$$

$$k_T = d_1 + \frac{d_2}{productivity} \quad \text{(Equation XVI)}$$

where a_1 and a_2, b_1 and b_2, c_1 and c_2, and d_1 and d_2 can replace empirical coefficients k_Y, k_0, k_V, and k_T, respectively, in **Equation XI**, resulting in the following equation to capture the relationship between MPSP and fermentation yield, titer, and productivity:

$$MPSP = \frac{a_1 + \frac{a_2}{productivity} + \frac{b_1 + \frac{b_2}{productivity}}{yield} + \frac{c_1 + \frac{c_2}{productivity}}{titer} + \frac{d_1 + \frac{d_2}{productivity}}{yield * titer}}{r} \quad \text{(Equation XVII)}$$

Supplementary Information:

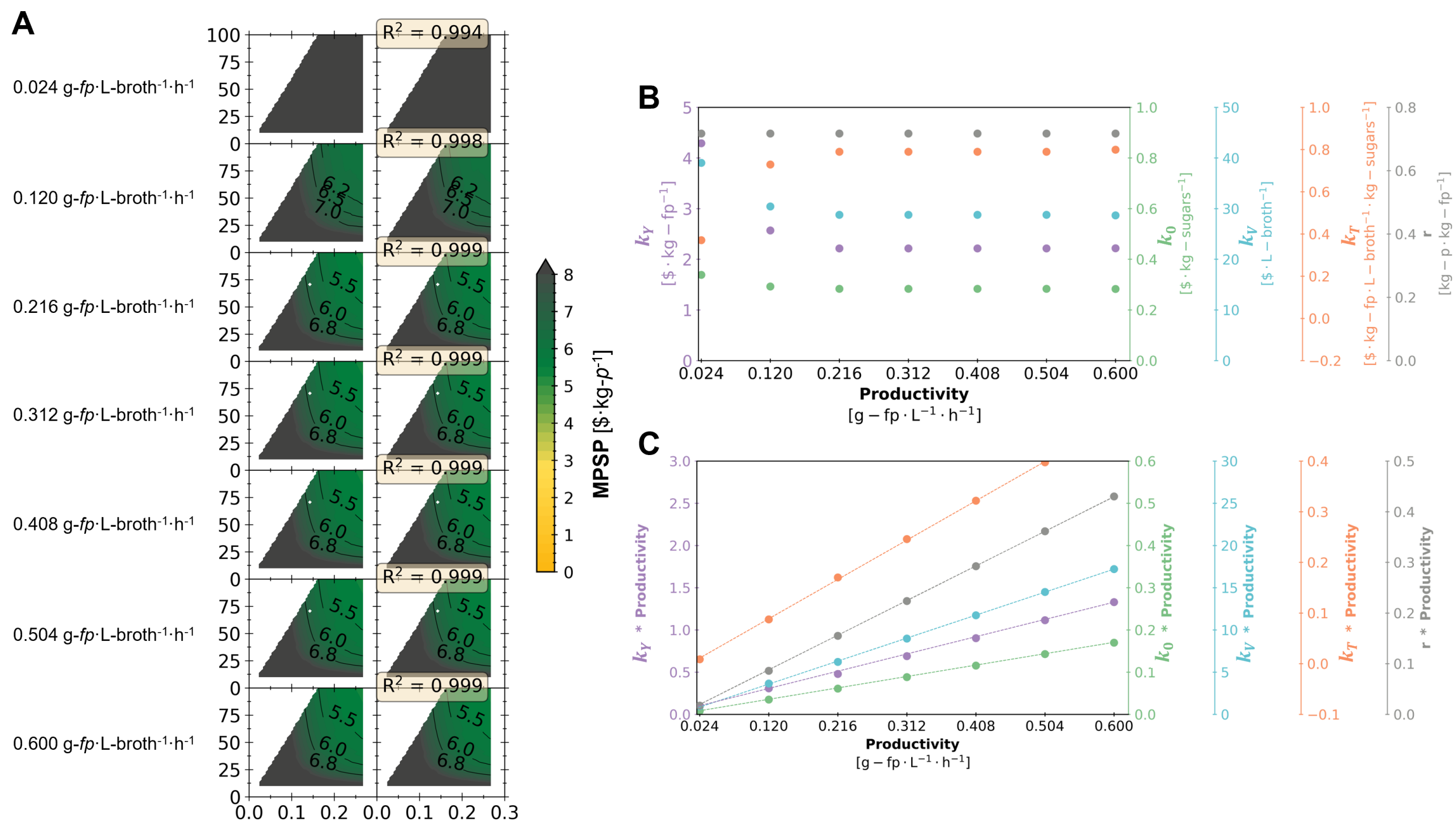

Figure S6. (A) Simulated values for MPSP across 2500 combinations of values for fermentation titer and yield at seven alternative productivities for biomanufacturing facilities producing sorbic acid via TAL from sugarcane (*left column panels*), and results from fitting **Equation XII** to simulation results (*right column panels*). (B) Values for coefficients of **Equation XII** when fit to simulation results for the baseline at alternative values for fermentation productivity. (C) Mathematical product of each coefficient value and fermentation productivity plotted at alternative values of fermentation productivity. Straight lines (dashed) passing through point values indicate the linear relationships described in **Equations XIII–XVI**.

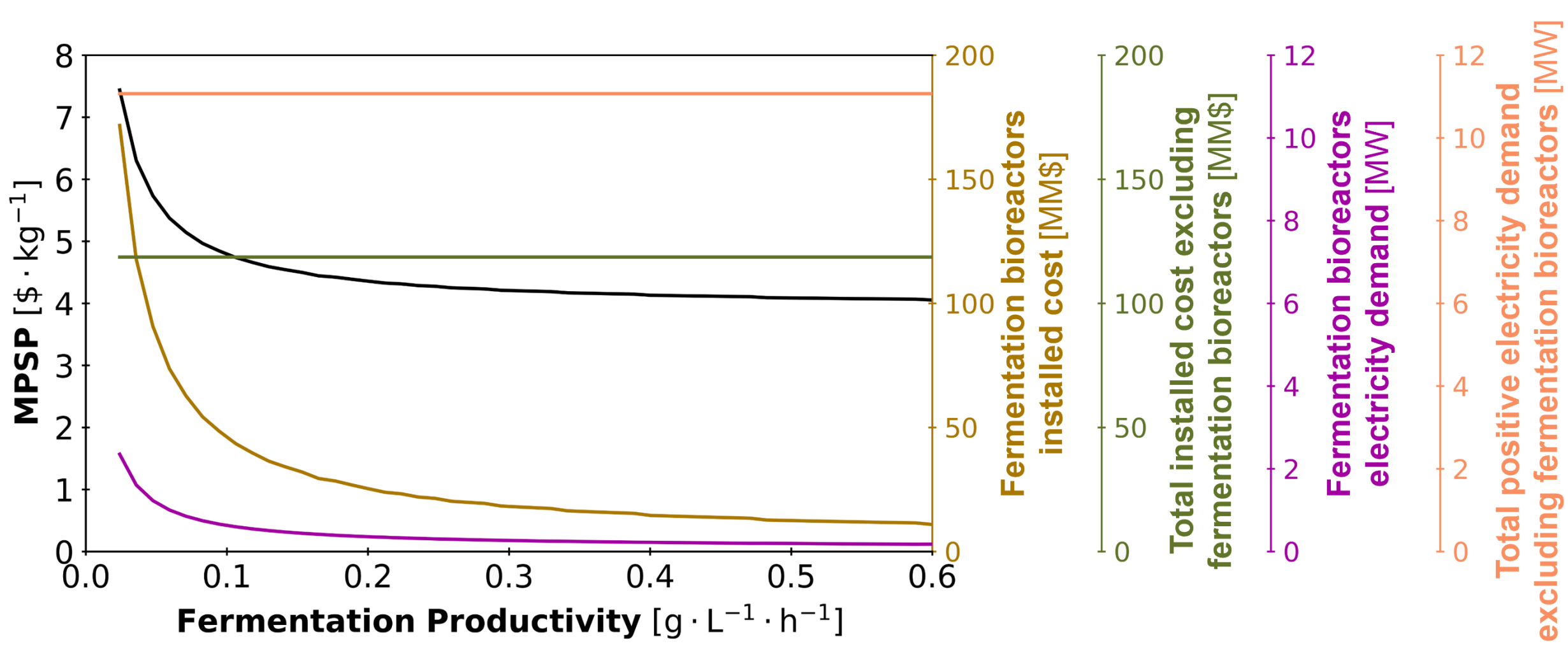

Figure S11. For biomanufacturing facilities producing TAL from sugarcane, the minimum product selling price ($·kg^{-1}), installed equipment cost of bioreactors for fermentation and the seed train (MM$), the total installed equipment cost of the facility excluding bioreactors for fermentation and the seed train (MM$), the electricity demand of bioreactors for fermentation and the seed train

(MW), and the total positive electricity demand of the facility excluding bioreactors for fermentation and the seed train (MW) simulated at 50 alternative values of fermentation productivity ranging from 0.024–6.000 $g \cdot L^{-1} \cdot h^{-1}$ are shown. Axis colors correspond to the colors of the curves.

1.2.2. *Sustainability framing versus economic scope. The title emphasizes "Fermentation Sustainability," yet the analysis focuses exclusively on economics (MPSP). While economic viability is necessary for sustainable biomanufacturing, sustainability encompasses environmental impacts (LCA), resource efficiency, and social factors. The authors mention carbon intensity briefly but do not integrate environmental metrics with their equation. I recommend either modifying the title to better reflect the economic focus (for example, "Fermentation Economics" or "Fermentation Viability"), or expanding the discussion to explicitly address how MPSP relates to broader sustainability metrics, with appropriate caveats about the scope of the current analysis.*

Response:
Thank you for this suggestion. The Reviewer correctly identified that the scope of this work did not include environmental impacts estimated by LCA, and we agree this should be clearly stated in the text. We have also added a discussion on the potential for future work to extend the proposed equation to capture life cycle environmental impacts. In all previous studies from which configurations were used in this study, facility life cycle inventory data were based on material and energy balances from process simulation, analogous to the annual operating cost in TEA. We found this equation is best at capturing system cost when annual operating cost is the predominant cost contributor rather than capital cost. Therefore, this equation could be used to capture life cycle environmental impacts such as carbon intensity (as 100-year global warming potential), fossil energy consumption (FEC), and others, with a system boundary that would exclude equipment acquisition and infrastructure construction (consistent with carbon intensity accounting requirements in the U.S. Renewable Fuel Standard, RFS). We have accordingly revised the **Conclusions** and added a new sub-section to the **Results** named *Key considerations for equation applicability* in which we discuss caveats related to the scope of the current analysis and highlight the potential for future work to expand this scope.

Revision:

Results:

[Lines 555–598]

Key considerations for equation applicability

Despite the robustness of the developed equation across vast design and thermodynamic landscapes simulated using multiple process simulators (BioSTEAM, Aspen Plus, and SuperPro Designer), it is worth noting a few considerations regarding the equation's applicability:

(i) All products of the biomanufacturing facilities explored in this work (triacetic acid lactone, potassium sorbate via triacetic acid lactone, acrylic acid via 3-hydroxypropionic acid, and succinic acid), as well as those explored in the external studies used for validation (ethanol, isobutanol, 1,3-propanediol, 3-hydroxypropionic acid, and 2,3-butanediol), were commodity chemicals simulated with large annual production capacities (>20,000 metric $ton \cdot y^{-1}$). Extending the proposed equation to smaller-scale facilities producing specialty chemicals, pharmaceuticals, and proteins may result in poor goodness of fit relative to the

large-scale facilities explored in this work as the exponentially scaling equipment purchase and installation costs are larger contributors to the MPSP at smaller scales and may not be adequately captured by the simple equation proposed in this work.

(ii) As is typical for biomanufacturing TEAs, this work and all external studies used for validation performed *n*th plant TEAs (i.e., it is assumed a successful industry has been established with mature technologies and supply chains). Established methods were used in this work for process design, simulation, and equipment sizing and costing, and rigorous uncertainty and sensitivity analyses were performed to avoid false precision.[1,2] However, it is worth noting that *n*th plant TEAs may not comprehensively capture the actual industrial costs experienced, especially by pioneer facilities (i.e., <*n*th plant). Such facilities navigate several challenges including navigating technological and scale-up hurdles, developing the workforce, establishing new supply chains and markets, and operating under highly variable conditions, thereby contributing toward maturing a nascent biomanufacturing industry.

(iii) This work was focused on economic indicators of sustainability and did not explore environmental impacts such as carbon intensity (e.g., as 100-year global warming potential) or fossil energy consumption. However, the proposed unifying equation could potentially be used to capture the relationship between a biomanufacturing facility's environmental impacts (estimated via life cycle assessment, LCA) and fermentation performance, as previously reported life cycle inventory data were based on each facility's material and energy balance flows from process design, simulation, and TEA.[3–6] A simplified form of the equation may be appropriate for life cycle impacts, as these typically exclude equipment acquisition and installation (consistent with carbon intensity accounting requirements in the U.S. Renewable Fuel Standard, RFS[7]). Consequently, it may not be necessary to represent the non-linear relationships associated with the scaling of equipment purchase and installation costs. However, future work to explore suitably modifying the proposed mathematical equation to capture how the biomanufacturing facility's life cycle environmental impacts are influenced by fermentation performance will be critical to navigating economic and environmental trade-offs.

Conclusions:

[Lines 671–675]

[…]. Finally, future work to explore suitably modifying the proposed mathematical equation to capture how the biomanufacturing facility's environmental impacts (estimated via life cycle assessment, LCA) are influenced by fermentation performance will be critical to navigating economic and environmental trade-offs.

1.2.3. *Independence of external validation. Many of the external validation studies appear to have been conducted using the same BioSTEAM platform, some by overlapping author groups. To strengthen confidence in generalizability, the authors should clearly indicate which external studies used independent simulation frameworks, discuss whether the equation's success might*

partly reflect consistency in BioSTEAM's embedded modeling assumptions, and consider validation against actual industrial cost data if available.

Response:
Thank you for this comment. The biomanufacturing process models used in this work were indeed developed using BioSTEAM by the authors in previous work (specifically, the 32 configurations for which we performed hundreds of thousands of simulations across fermentation titer-rate-yield combinations and under uncertainty in this work; Figures 1 and 3, and Figures S1, S2, S3, S6, and S8–S10 in the SI). These explored the fermentation products 3-hydroxypropionic acid, triacetic acid lactone, and succinic acid.

However, the data used for validation was from external TEA studies (no overlapping authors) using the tools Aspen Plus and SuperPro Designer, not BioSTEAM. Previously, these were for fermentation products ethanol, 3-hydroxypropionic acid, and 2,3-butanediol. In this revision, we have also added new validation data for fermentation products 1,3-propanediol (under aerobic and microaerobic conditions) and isobutanol. This revision is reflected in the updated Figure S7 in the SI. Note that while no industrial cost data was available for detailed comparison, TEAs in both BioSTEAM and the commercially established Aspen Plus and SuperPro Designer all update equipment purchase and installation prices to the TEA year using the Chemical Engineering Plant Cost Indices (CEPCI, https://www.chemengonline.com/pci-home/) and raw material purchase and co-product selling prices to the TEA year using Chemical Price Indices (from the US bureau of labor statistics, https://www.bls.gov/mxp/publications/industry-pamphlets/charts/chemical-price-indexes.htm).

We agree with the reviewer that the fact that the external studies used for validation had used Aspen Plus and SuperPro Designer (not BioSTEAM) could be better highlighted to underscore the generalizability of our proposed equation. It could also be made clearer that the proposed equation is intended to emulate rigorous TEAs. In addition to adding new data for external validation (reflected in the updated **Figure S7** in the **SI**), we have accordingly revised the **Results** (by adding two new sub-sections, *Validation with external studies and alternative process simulators* and *Key considerations for equation applicability*) and **Conclusions** as detailed below.

Revision:

Results:

[Lines 514–553]

Validation with external studies and alternative process simulators
The 32 biomanufacturing facilities used to extract the unifying equation proposed in this work (simplified form shown in **Equation IV**; the form used for physical interpretation of coefficients is shown in **Equation XII**) were all developed in BioSTEAM by the same authors and involved three fermentation products (triacetic acid lactone, 3-hydroxypropionic acid, and succinic acid) and four final products (triacetic acid lactone, potassium sorbate via triacetic acid lactone, acrylic acid via 3-hydroxypropionic acid, and succinic acid) from four feedstocks (dextrose monohydrate, sugarcane, corn, and corn stover) involving four distinct separation processes (overview of these 32 biomanufacturing configurations shown in **Figure 5**). To further ascertain the generalizability of the proposed equation, we also sought to validate results using data from external studies that explored biomanufacturing configurations with different fermentation products and separation processes using different process simulator tools (e.g., Aspen Plus, SuperPro Designer).

Aside from studies published by the authors of this study, we found three previous studies that simulated biomanufacturing facilities across multiple combinations of fermentation titer and/or yield and estimated MPSP at those combinations. Using SuperPro Designer, Gunukula *et al.*[8] designed biomanufacturing facilities: (i) producing isobutanol from corn using an isobutanol separation process that involved distillation and decantation; (ii) producing 3HP from corn using a 3HP separation process involving solvent extraction (using methyl isobutyl ketone) and distillation; (iii) producing 1,3-propanediol from corn under microaerobic fermentation conditions and using a 1,3-propanediol separation process that involved solvent extraction (using hexanol) and distillation; and (iv) producing 1,3-propanediol from corn under aerobic fermentation conditions and using the same separation process as (iii). Gunukula *et al.* simulated the biomanufacturing facilities across fermentation titer and yield of the respective fermentation products and estimated the MPSP at each simulated combination of fermentation yield and titer. Using Aspen Plus, Janković *et al.*[9] designed a biomanufacturing facility separating pure ethanol from a crude ethanol stream through a process involving vacuum distillation and extractive distillation (using ethylene glycol), and estimated the annual cost of separation across alternative values for ethanol titer in the crude stream. Finally, using Aspen Plus, Sikazwe *et al.*[10] designed a biomanufacturing facility producing 2,3-butanediol from sugarcane A-molasses that used a 2,3-butanediol separation process involving solvent extraction (using oleyl alcohol) and distillation, and estimated the MPSP across alternative combinations of fermentation yield and titer.

We fit **Equation IV** to the results for each of these six biomanufacturing configurations that external studies had simulated and evaluated to estimate MPSP across fermentation yield and/or titer. Across this diverse design and simulation landscape explored using Aspen Plus and SuperPro Designer in the external studies, we found a high goodness of fit (coefficient of determination R^2 of 0.954–1.000; **Figure S7** in the SI), comparable to the goodness of fit for the 32 biomanufacturing configurations simulated in this work (R^2 of 0.992–1.000). This demonstrates the unifying equation proposed in this work is generalizable across nth plant TEAs with process designs involving diverse feedstocks, fermentation processes and products, separation processes, and catalytic upgrading regimes to produce commodity chemicals.

Key considerations for equation applicability

Please see our response to Comment 1.2.2 for updated text. This includes 4 new paragraphs to address these points.

Conclusions:

[Lines 601–609]

In this study, we proposed a simple, physically interpretable mathematical equation (**Equation XII**; further simplified form shown in **Equation IV**) that robustly captures the relationship between fermentation performance (titer, rate or productivity, and yield, TRY) and system cost (R^2 of 0.992−1.000) under diverse assumptions for biomanufacturing facility design decisions, technological performance, and contextual parameters (32 distinct biomanufacturing feedstock-to-product configurations simulated under uncertainty and across 240,000 combinations of fermentation TRY using BioSTEAM), and validated it using TEA results from external studies that explored six distinct biomanufacturing feedstock-to-product configurations using Aspen Plus and SuperPro Designer (R^2 of 0.954−1.000 on results from external studies).

Supplementary Information:

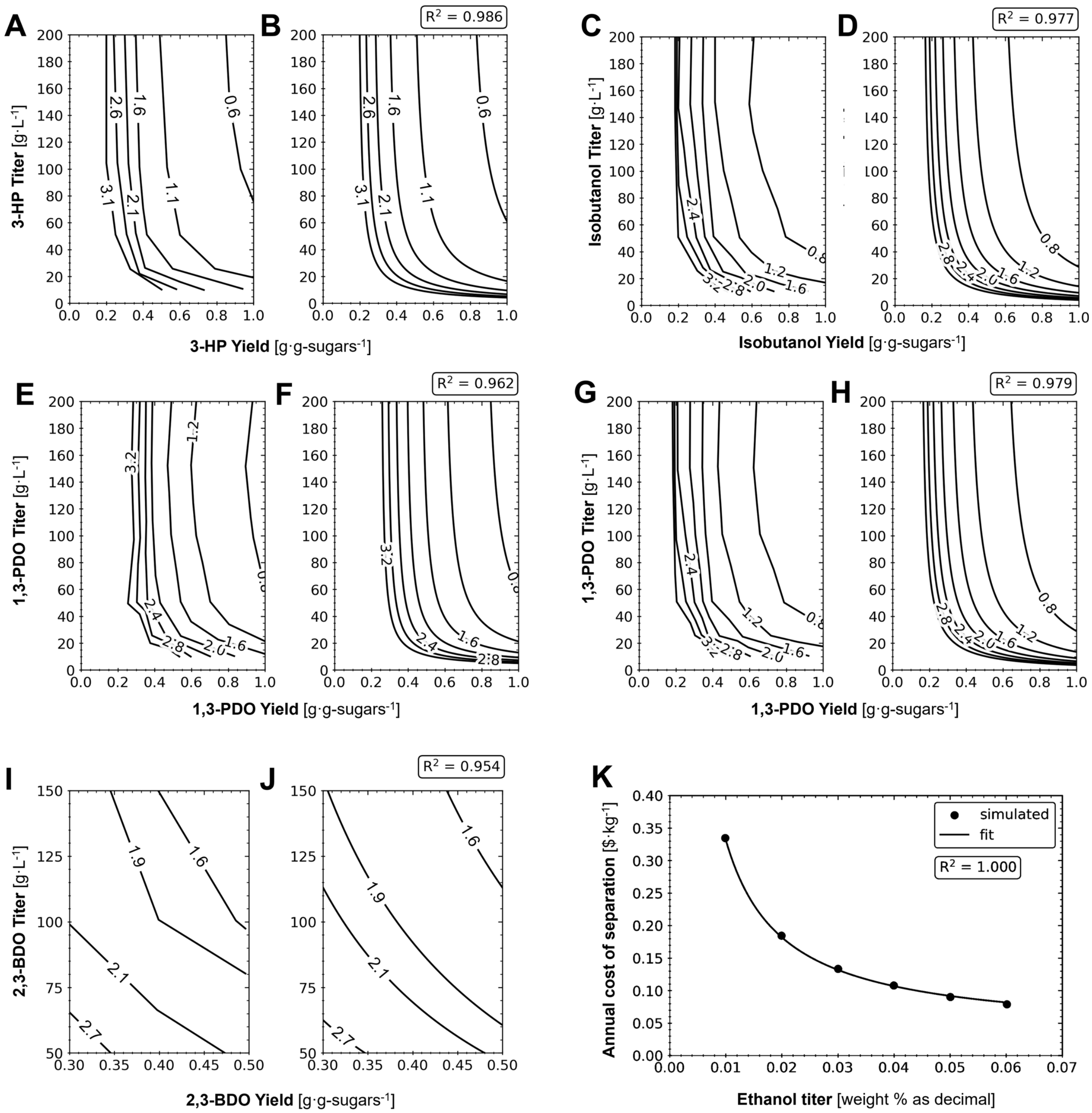

Figure S7. Minimum product selling price (inline contour labels) as a function of fermentation yield and titer of: (A) 3-hydroxypropionic acid (3-HP) from Gunukula *et al.*, who used SuperPro Designer;[11] (C) isobutanol from Gunukula *et al.*, who used SuperPro Designer;[11] (E) 1,3-propanediol (1,3-PDO) produced under aerobic conditions from Gunukula *et al.*, who used SuperPro Designer;[11] (G) 1,3-propanediol (1,3-PDO) produced under microaerobic conditions from Gunukula *et al.*, who used SuperPro Designer;[11] (I) 2,3-butanediol from Sikazwe *et al.*, who used Aspen Plus.[10] Results of fitting the empirical coefficients *a*, *b*, *c*, and *d* in **Equation IV** to the data from (B, D, F, H) Gunukula *et al.* and (J) Sikazwe *et al.* are shown, with values for coefficient of determination (R^2) included near the top-right corner of each panel. (K) Annual cost of separation (*y*-axis) of ethanol at alternative values of crude ethanol concentration (*x*-axis) from Janković *et al.*[9], who used Aspen Plus (*simulated*), and the results of fitting the empirical coefficients *a* and *c* in **Equation IV** (*fit*; *b* and *d* were set to zero) to the data from Janković *et al.*, with an R^2 of 1.000.

1.2.4. *Limitations section. The manuscript would benefit from a brief dedicated section discussing the current product scope (commodity chemicals) and untested extensions (fine chemicals, pharmaceuticals, proteins), the absence of industrial-scale cost validation, and assumptions embedded in the BioSTEAM models.*

Response:
We thank the Reviewer for this suggestion. We have accordingly added a new sub-section to the **Results** named ***Key considerations for equation applicability*** to discuss the limitations of this work, including those mentioned by the Reviewer.

Revision:

Results:

Key considerations for equation applicability

Please see our response to Comment 1.2.2 for updated text. This includes 4 new paragraphs to address these points.

1.2.5. *Coefficient interpretation. The Discussion could be strengthened by providing more physical interpretation of the fitted coefficients (a, b, c, d) and how their relative magnitudes reveal underlying cost drivers across different facility configurations.*

Response:
We thank the reviewer for this suggestion and agree with the value of providing physical interpretation of the fitted equation coefficients. Indeed, we have included a detailed discussion in the **Results** under *Uncertainties and general drivers influencing the relationship between fermentation performance and system cost* (now named *Physical interpretation of equation coefficients and cost drivers under uncertainty*). However, we agree that we could expand on this discussion and better highlight existing discussion of physical interpretations. Therefore, in addition to changing the symbols of the four equation coefficients across the manuscript and SI to better highlight their physical interpretations, we have accordingly expanded the discussion of physical interpretations of equation coefficients in the **Results** and **Conclusions**.

Revision:

Results:

[Lines 301–431]

Physical interpretation of equation coefficients and cost drivers under uncertainty
[…]. Values of coefficient k_Y (the facility's yield-dependent burden coefficient, which influences costs that increase linearly with fermentation yield, but not titer; **Equation XII**) were consistently higher for neutral fermentation regimes than for low-pH fermentation regimes. For example, […]. Therefore, fermentation regime (neutral vs. low-pH), catalytic upgrading, and the design of separations downstream of any initial separation of water from fermentation broths (to a target concentration of *fp*) were key drivers of system cost but did not influence the relationship between fermentation performance and system cost (governed by coefficient k_Y; **Equation XII**).

Values of coefficient k_0 (the facility's TRY-invariant burden coefficient, which influences costs that are independent of fermentation yield and titer; **Equation XII**) were very similar for biomanufacturing facilities accepting the same feedstock. For example, k_0 […]. Therefore, feedstock unit price, the recoverable energy in the waste fractions of the feedstock and fermentation broth, and the efficiency of energy recovery in WWRR and CHP were key drivers influencing the relationship between fermentation yield and system cost (governed by coefficient k_0; **Equation XII**).

Coefficient k_V (the facility's broth volumetric flow-dependent burden coefficient, which influences costs that increase linearly with the ratio of fermentation yield to titer; **Equation XII**) had similar values for biomanufacturing facilities with the same separation process, regardless of the inclusion of catalytic upgrading. For example, k_V was […]. Therefore, the unit prices and loadings for fermentation nutrient sources (such as CSL) and the design of separations including and upstream of any initial separation of water from fermentation broths (to a target concentration of *fp*) were key drivers influencing the relationship between fermentation titer and system cost (governed by coefficient k_V; **Equation XII**).

Across the 24 biomanufacturing facilities evaluated under uncertainty, k_T (the facility's titer-only dependent burden coefficient, which influences costs that depend on titer but not on yield) […]. Over the evaluated theoretical fermentation space […] indicating k_T did not substantially influence the relationship between fermentation performance and system cost.

Conclusions:

[Lines 618–640]

[…]. In exploring the physical interpretations of the coefficients of the proposed mathematical equation by simulating biomanufacturing facilities under uncertainty (600,000 Monte Carlo simulations), we revealed key drivers and non-drivers of the relationship between fermentation TRY and system cost. By interpreting design, technological, and contextual drivers of the facility's yield-dependent burden coefficient k_Y, we found that while the fermentation regime (neutral vs. low pH) and the inclusion of catalytic upgrading influenced system cost, they did not substantially alter how fermentation TRY influenced system cost. Similarly, costs associated with the separation process equipment farther downstream (specifically, downstream of separation equipment that removed water from the fermentation broth to achieve a target concentration) influenced system cost, but did not substantially alter the relationship between fermentation performance and system cost. Further, by interpreting the facility's TRY-invariant burden coefficient k_0, we found the choice of feedstock and the energy content of the waste fractions of the feedstock and fermentation broth substantially affected the influence of fermentation yield on system cost—more expensive feedstocks and lower total recoverable energy in wastes (by WWRR and CHP) amplified this influence. Finally, by interpreting the facility's broth volumetric flow-dependent burden coefficient k_V, we found costs associated with the separation process equipment immediately downstream of fermentation (specifically, including and upstream of equipment that first removed water from the fermentation broth to achieve a target concentration rather than a target amount of water removal) substantially affected the influence of titer on system cost—more expensive separations including and upstream of target concentration-based water removal amplified this influence. Similarly, an increase in costs associated with non-sugar, fixed-concentration nutrient additives to the fermentation medium (e.g., to satisfy nitrogen and phosphorus requirements) increased the influence of titer on system cost.

REVIEWER 2

2.1. General Comments:

This work is well designed and performed, which is an important topics for economic biomanufacturing but neglected due to the lack of data and methodology.

Response:
We thank the Reviewer for their positive assessment of the work and for their constructive comments. We have addressed the comments raised by the Reviewer and the responses are detailed below.

2.2. Specific Comments:
2.2.1. *Although the manuscript is well-written, there are still typos with long paragraphs, which needs to be improved.*

Response:
Thank you for drawing our attention to the typographical errors and lengthy paragraphs. We have revised the manuscript text accordingly, as shown below.

Revision:

Introduction:

[Lines 52–55]

[…]. Industrial fermentation—the use of naturally occurring or genetically engineered microorganisms to produce targeted chemicals—has been identified as […] fuels and commodity chemicals.[12–15]

Results:

[Lines 120–123] […] and two catalytic upgrading processes (3HP [...]; TAL [...]). All biomanufacturing facility configurations are shown in **Figure 5** [...].

[Line 137] […] at each point (32 left-hand side panels in **Figure 1** and **Figures S1** and **S2** in the SI).

[Lines 140–143] […] across fermentation yield and titer (coefficient of determination, R^2 of 0.992−1.000 across 7500 yield-titer-productivity combinations each for 32 biomanufacturing facilities; 32 right-hand side panels in **Figure 1** and **Figures S1** and **S2** in the SI) […].

[Lines 251–257] […]. For biomanufacturing facilities with […] productivity (**Figures S4** and **S7B** in the SI). Thus, **Equation IV** […] facilities (R^2 of 0.992−1.000; **Figure 1** and **Figures S1** and **S2** in the SI).

[Line 257] […] R^2 of 0.992−1.000; **Figure 1** and **Figures S1** and **S2** in the SI […].

[Lines 322–328] […]. From the parameters included in the uncertainty analyses that […] relative to fermentation yield. This was consistent with […] as titer increased (**Figure S4C** in the SI).

[Lines 359–364] […]. Finally, for all 24 biomanufacturing facilities […] product storage time (**Table S5** in the SI). This was consistent with […] and titer (**Figure S4** in the SI).

[Line 467–469] For all 32 biomanufacturing facilities […] fermentation performance space (**Figure 4**).

[Lines 499–505] […]. For biomanufacturing facilities with the same fermentation product and feedstock, the […] (e.g., […]; **Figure 4**). This was consistent with k_0 and k_T remaining relatively unchanged and […] (**Figure 3B,C,D**).

[**Figure 1 caption**] […]. Rows represent biomanufacturing facilities producing TAL (*TAL*), potassium sorbate via TAL (*potassium sorbate via TAL*), acrylic acid via 3HP (*acrylic acid via 3HP*) under low-pH (*low-pH*) and neutral fermentation regimes (*neutral*) with separation process A (*separation-A*)[16] or B (*separation-B*)[4], and succinic acid (*succinic acid*) under low-pH (*low-pH*) and neutral fermentation regimes (*neutral*). Columns represent biomanufacturing facilities accepting dextrose monohydrate (*glucose*), sugarcane (*sugarcane*), corn stover (*corn stover*), and corn (*corn*) as the feedstock. All biomanufacturing facility configurations are described in **Figure 5**.

[**Figure 4 caption**] […]. Rows represent biomanufacturing facilities producing TAL (*TAL*), potassium sorbate via TAL (*potassium sorbate via TAL*), acrylic acid via 3HP (*acrylic acid via 3HP*) under low-pH (*low-pH*) and neutral fermentation regimes (*neutral*) with separation process A (*separation-A*)[16] or B (*separation-B*)[4], and succinic acid (*succinic acid*) under low-pH (*low-pH*) and neutral fermentation regimes (*neutral*). Columns represent biomanufacturing facilities accepting dextrose monohydrate (*glucose*), sugarcane (*sugarcane*), corn stover (*corn stover*), and corn (*corn*) as the feedstock. All biomanufacturing facility configurations are described in **Figure 5**.

Methods:

[Lines 685–687] […] and miscellaneous facilities. An overview of biomanufacturing facility processes and the configurations of the 32 biomanufacturing facilities evaluated in this study is provided in **Figure 5** and in **Table S1** in the SI.

[Lines 703–717] […]. Models for all process alternatives were from previous studies; specifically: (i) corn dry grind; (ii) sugarcane juicing and clarification; (iii) corn stover pretreatment and saccharification;[1] (iv) fermentative TAL production;[5] (v) fermentative 3HP production;[17] (vi) fermentative succinic acid production;[3] (vii) TAL separation by crystallization;[5] (viii) 3HP separation by adsorption, ion exchange, and evaporation (separation-A);[16] (ix) 3HP separation by hexanol-based solvent extraction (separation-B);[4] (x) succinic acid separation by evaporation and crystallization;[3] (xi) TAL catalytic upgrading to potassium sorbate;[6] and (xii) 3HP catalytic upgrading to acrylic acid.[4] All listed models are available open-source in the online repositories.[18–21]

[Adjacent sentence with repeating information removed.]

Supplementary Information:

[*Section S1.1. Fermentation Processes*] […]. This manifests as an infeasible region in the upper-left portion of the fermentation titer-yield space (represented by white regions in **Figures 1**, **S1, S2**, **S3**, **S5**, **S6**, **S8**, **S9**, and **S10**).

[**Figure S1 caption**] […]. Rows represent biomanufacturing facilities producing TAL (*TAL*), potassium sorbate via TAL (*potassium sorbate via TAL*), acrylic acid via 3HP (*acrylic acid via*

3HP) under low-pH (*low-pH*) and neutral fermentation regimes (*neutral*) with separation process A (*separation-A*)[16] or B (*separation-B*)[4], and succinic acid (*succinic acid*) under low-pH (*low-pH*) and neutral fermentation regimes (*neutral*). Columns represent biomanufacturing facilities accepting dextrose monohydrate (*glucose*), sugarcane (*sugarcane*), corn stover (*corn stover*), and corn (*corn*) as the feedstock. All biomanufacturing facility configurations are described in **Figure 5**.

[**Figure S2 caption**] […]. Rows represent biomanufacturing facilities producing TAL (*TAL*), potassium sorbate via TAL (*potassium sorbate via TAL*), acrylic acid via 3HP (*acrylic acid via 3HP*) under low-pH (*low-pH*) and neutral fermentation regimes (*neutral*) with separation process A (*separation-A*)[16] or B (*separation-B*)[4], and succinic acid (*succinic acid*) under low-pH (*low-pH*) and neutral fermentation regimes (*neutral*). Columns represent biomanufacturing facilities accepting dextrose monohydrate (*glucose*), sugarcane (*sugarcane*), corn stover (*corn stover*), and corn (*corn*) as the feedstock. All biomanufacturing facility configurations are described in **Figure 5**.

[**Figure S9 caption**] […]. Rows represent biomanufacturing facilities producing TAL (*TAL*), potassium sorbate via TAL (*potassium sorbate via TAL*), acrylic acid via 3HP (*acrylic acid via 3HP*) under low-pH (*low-pH*) and neutral fermentation regimes (*neutral*) with separation process A (*separation-A*)[16] or B (*separation-B*)[4], and succinic acid (*succinic acid*) under low-pH (*low-pH*) and neutral fermentation regimes (*neutral*). Columns represent biomanufacturing facilities accepting dextrose monohydrate (*glucose*), sugarcane (*sugarcane*), corn stover (*corn stover*), and corn (*corn*) as the feedstock. All biomanufacturing facility configurations are described in **Figure 5**.

[**Figure S10 caption**] […]. Rows represent biomanufacturing facilities producing TAL (*TAL*), potassium sorbate via TAL (*potassium sorbate via TAL*), acrylic acid via 3HP (*acrylic acid via 3HP*) under low-pH (*low-pH*) and neutral fermentation regimes (*neutral*) with separation process A (*separation-A*)[16] or B (*separation-B*)[4], and succinic acid (*succinic acid*) under low-pH (*low-pH*) and neutral fermentation regimes (*neutral*). Columns represent biomanufacturing facilities accepting dextrose monohydrate (*glucose*), sugarcane (*sugarcane*), corn stover (*corn stover*), and corn (*corn*) as the feedstock. All biomanufacturing facility configurations are described in **Figure 5**.

2.2.2. *My suggestions are to take the theoretical yield of the target product of the producing microorganism into consideration for the uncertainty study, to consider the situations of anaerobic and aerobic fermentation, and to include other products that are currently for commercial production besides the three ones in this study such as ethanol, 1,3-PDO, 1,4-BDO, and amino acids as well as single cell protein and PHA. Will the equation fit diverse products of different fermentation conditions?*

Response:
Thank you for these comments. We agree with the Reviewer that the theoretical maximum yield is an important consideration. To clarify, for the three fermentation products (3-hydroxypropionic acid, succinic acid, and triacetic acid lactone) involved in the design landscape we explored in this work, the simulated ranges for fermentation product yields each had a higher bound equal to the maximum value at which: (i) the total glucose conversion (alongside other reactions for microbial cell mass formation, co-product formation, and respiration, based on their reported

baseline yields) did not exceed 100%; and (ii) the fermentation product yield did not exceed the theoretical maximum yield. The additional considerations of cell mass formation, co-product yield, and respiration were to avoid making implicit assumptions about which of those three reaction yields would decrease to accommodate an increase in fermentation product yield. We agree with the Reviewer that these considerations could be better highlighted in the text and we have accordingly revised the **Methods** section.

We also agree with the Reviewer about the need to validate our unifying equation with other commercial products and under different fermentation conditions. Indeed, in addition to the three fermentation products explored in our study, we had originally included validation results using data from external studies that used different process simulators (Aspen Plus and SuperPro Designer) on two different products (ethanol, 2,3-butanediol) and one in common with this study (3-hydroxypropionic acid). However, we agree that the data used for validation could be expanded to other commercial products and different fermentation conditions. We have accordingly revised the text and **Figure S7**, and the expanded validation data therein now includes:

(i) ethanol (as before);
(ii) 2,3-butanediol (as before);
(iii) 3-hydroxypropionic acid (as before);
(iv) 1,3-propanediol under aerobic conditions (added in this revision);
(v) 1,3-propanediol under microaerobic conditions (added in this revision); and
(vi) isobutanol (added in this revision).

Further, we have expanded the discussion of the validation results in a new sub-section of the **Results** named *Validation with external studies and alternative process simulators*. We have also added a new sub-section to the **Results** named *Key considerations for equation applicability* that discusses limitations related to the types of products and facilities suitable for applying the developed equation, among other limitations. Finally, we have revised the **Conclusions** to better highlight the validation efforts performed in this work.

Revision:

Methods:

System analyses under uncertainty

[Lines 764–780]

Uncertainty and Sensitivity Analyses

[…]. For each of the 32 biomanufacturing facilities evaluated in this work (**Figure 5**), 2500 potential combinations of fermentation yield and titer were evaluated at 3 alternative values of fermentation productivity (20%, 100%, and 500% of each of the reported baseline productivity values of 0.12 g-*fp*·L^{-1}·h^{-1} for TAL, 0.28 g-*fp*·L^{-1}·h^{-1} for 3HP, and 0.66 g-*fp*·L^{-1}·h^{-1} for succinic acid) for a total of 240,000 simulations.[3–5] For fermentation yield, the lower bounds for evaluation were set to ~5% of the theoretical maximum yields to ensure non-zero product recovery (the theoretical maximum yields were 0.467 g·g^{-1} glucose-eq. for TAL, 1.000 g·g^{-1} glucose-eq. for 3HP, and 1.31 g·g^{-1} glucose-eq. for succinic acid) and the upper bounds were each set to the maximum value at which: (i) the total glucose conversion (alongside other reactions for microbial cell mass formation, co-product formation, and respiration, based on their reported baseline yields[3–5]) did not exceed 100%; and (ii) the fermentation product yield did not exceed the theoretical maximum yield. For

fermentation titer, the upper bounds for evaluation were set to 100 g·L^{-1} for TAL (note that the utilized process models adequately characterize the implications of the low solubility of TAL in water[5]), 200 g·L^{-1} for 3HP, and 120 g·L^{-1} for succinic acid based on the bounds used for evaluation in previous studies.[3–5]

Results:

Validation with external studies and alternative process simulators

Please see the new text in this section (3 new paragraphs) presented in our response to Comment 1.2.3.

Key considerations for equation applicability

Please see the new text in this section provided in response to Comment 1.2.2. This includes 4 new paragraphs to address these points.

Conclusions:

[Lines 601–609]

In this study, we proposed a simple, physically interpretable mathematical equation (**Equation XII**; further simplified form shown in **Equation IV**) that robustly captures the relationship between fermentation performance (titer, rate or productivity, and yield, TRY) and system cost (R^2 of 0.992−1.000) under diverse assumptions for biomanufacturing facility design decisions, technological performance, and contextual parameters (32 distinct biomanufacturing feedstock-to-product configurations simulated under uncertainty and across 240,000 combinations of fermentation TRY using BioSTEAM), and validated it using TEA results from external studies that explored six distinct biomanufacturing feedstock-to-product configurations using Aspen Plus and SuperPro Designer (R^2 of 0.954−1.000 on results from external studies).

Supplementary Information:

*Please see the new **Figure S7** and corresponding caption in our response to Comment 1.2.3.*

REVIEWER 3

3.1. General Comments:

Bhagwat et al. provide a comprehensive analysis to demonstrate that complex process and economic models can be easily modeled using a small number of parameters. The approach is thorough, using a complex process design with 32 unique arrangements of feedstocks, processes, and products. Their approach is supported with Monte Carlo simulations and sensitivity analyses. The proposed approach for simplifying complex analyses offers a unique perspective, but additional clarification and validation is needed. I recommend the manuscript for major revision before consideration for publication.

Response:
We thank the Reviewer for their positive assessment of the work and for their constructive comments. We have addressed the comments raised by the Reviewer and the responses are detailed below.

3.2. Specific Comments:
3.2.1. *The thesis presented by the authors is that a complex process model could be fit by a simple linear model that relies on a small number of parameters. But wouldn't a multivariate linear regression produce the same results, identifying the same key factors that have the most significant influence on MPSP (e.g., MPSP = a + b*X_1 + c*X_2 + d*X_1*X_2)? Can the authors better explain how their approach differs from existing linear regression methods? Would the paper be strengthened if the authors provided a direct comparison of their results against the results from a linear regression approach?*

Response:
Thank you for this suggestion. We agree it would be worth exploring these alternative equations for regression to compare against our proposed unifying equation. For the equation suggested by the Reviewer, *MPSP = a + b*X_1 + c*X_2 + d*X_1*X_2*, substituting *X_1* for 1/ *yield* and *X_2* for 1/*titer* would result in the same equation as the simplified form of the equation we developed in this study (Equation IV, $MPSP = a + \frac{b}{yield} + \frac{c}{titer} + \frac{d}{yield*titer}$). To ensure we capture the alternative approaches suggested by the Reviewer, we instead substitute *X_1* for *yield* and *X_2* for *titer*.

We have accordingly revised the **Results** section and the **Supplementary Information** (SI) to now include a direct comparison of the results from using our unifying equation against two alternative regression approaches:

(i) Multivariate linear regression (*MPSP = i + j*yield + k*titer*; **Figure S9** in the SI)
(ii) Regression using the alternative equation form suggested by the Reviewer (*MPSP = i + j*yield + k*titer + m*yield*titer*; **Figure S10** in the SI)

Revision:

Results:

[Lines 203–213]

Generalized trends in how fermentation performance influences system cost

[…] To assess the relative suitability of the developed equation, we compared our approach against two alternative regression approaches: (i) three-coefficient multivariate linear regression with fermentation yield and titer as variables (**Figure S9** in the SI); and (ii) adding a non-linear term to the equation from (i) to obtain a four-coefficient equation with fermentation yield and titer as variables (**Figure S10** in the SI). Across simulation results for the 32 biomanufacturing configurations, fitting the developed equation (**Equation IV**) resulted in a substantially and consistently higher goodness of fit (coefficient of determination R^2 of 0.992–1.000; **Figure 1** and **Figures S1** and **S2** in the SI) than the two alternative regression approaches (R^2 of 0.573–0.704 and 0.586–0.838, respectively; **Figures S9** and **S10** in the SI), indicating the developed equation (**Equation IV**) represented the wide design and thermodynamic simulation landscape across biomanufacturing configurations more robustly than the alternative approaches evaluated.

Supplementary Information:

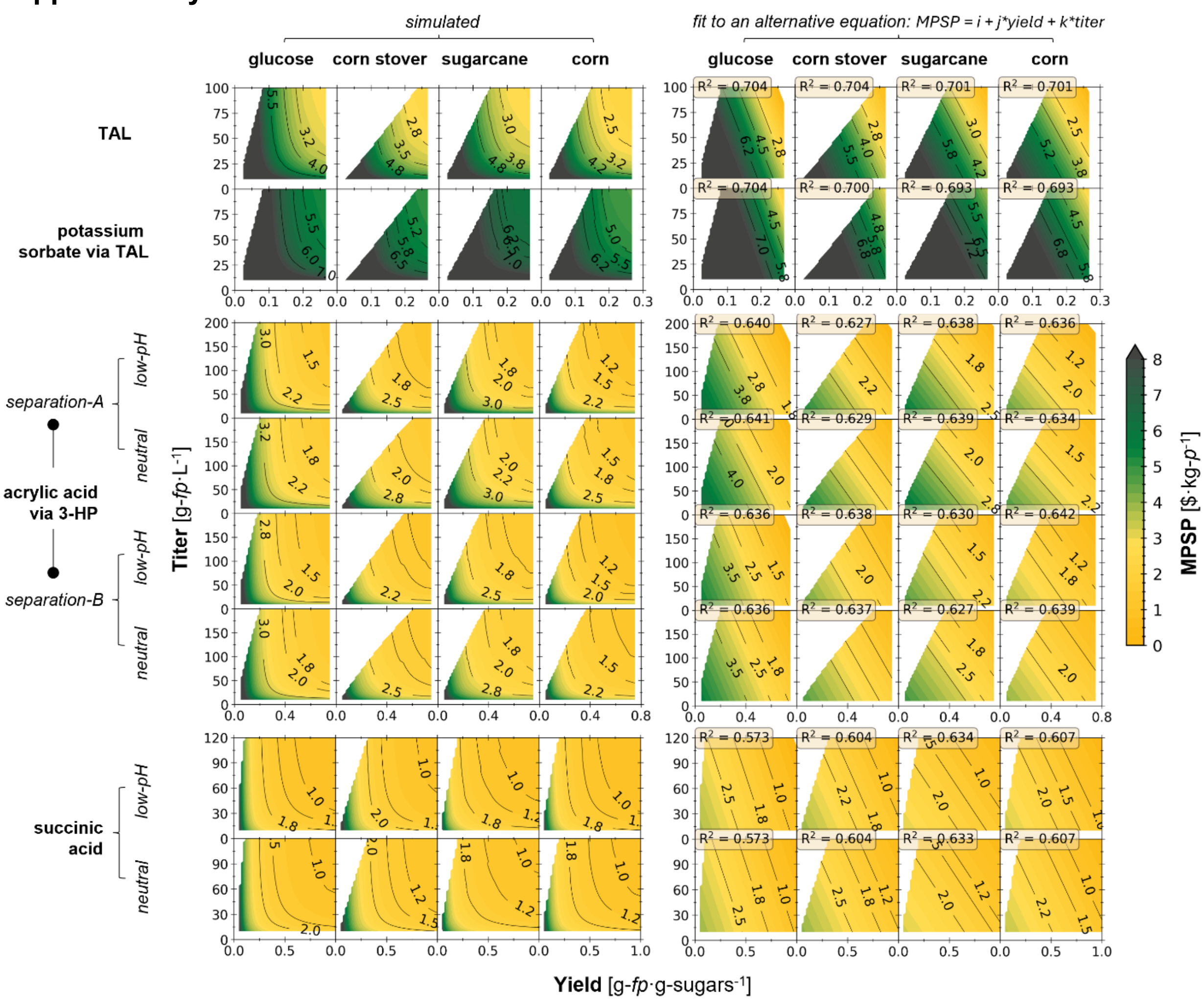

Figure S9. Minimum product selling price (MPSP) of the main biomanufacturing facility product (*p*) across fermentation yields (*x*-axes) and titers (*y*-axes) at one-fifth the baseline productivity values (i.e., at 0.024 g-*fp*·L^{-1}·h^{-1} for TAL biomanufacturing facilities, 0.066 g-*fp*·L^{-1}·h^{-1} for 3HP biomanufacturing facilities, and 0.132 g-*fp*·L^{-1}·h^{-1} for succinic acid biomanufacturing facilities). For a given point on the figure, the *x*-axis value represents the overall fermentation yield (g-*fp*·g-sugars^{-1}), the *y-axis* value represents the titer (g-*fp*·L^{-1}), and the color represents MPSP. The white region to the upper left of each plot represents infeasible yield-titer combinations (described in **Section S1.1** of the **SI**). Panels in the first four columns show results from simulation and

evaluation of biomanufacturing facilities at 2500 fermentation yield-titer combinations each, while panels in **the last four columns show results from fitting to an alternative equation (multivariate linear regression, i.e., fitting *MPSP = i + j*yield + k*titer*, where *i*, *j*, and *k* are empirical coefficients)**. Rows represent biomanufacturing facilities producing TAL (*TAL*), potassium sorbate via TAL (*potassium sorbate via TAL*), acrylic acid via 3HP (*acrylic acid via 3HP*) under low-pH (*low-pH*) and neutral fermentation regimes (*neutral*) with separation process A (*separation-A*) or B (*separation-B*), and succinic acid (*succinic acid*) under low-pH (*low-pH*) and neutral fermentation regimes (*neutral*). Columns represent biomanufacturing facilities accepting dextrose monohydrate (*glucose*), sugarcane (*sugarcane*), corn stover (*corn stover*), and corn (*corn*) as the feedstock (all biomanufacturing facility configurations are described in **Figure 5**).

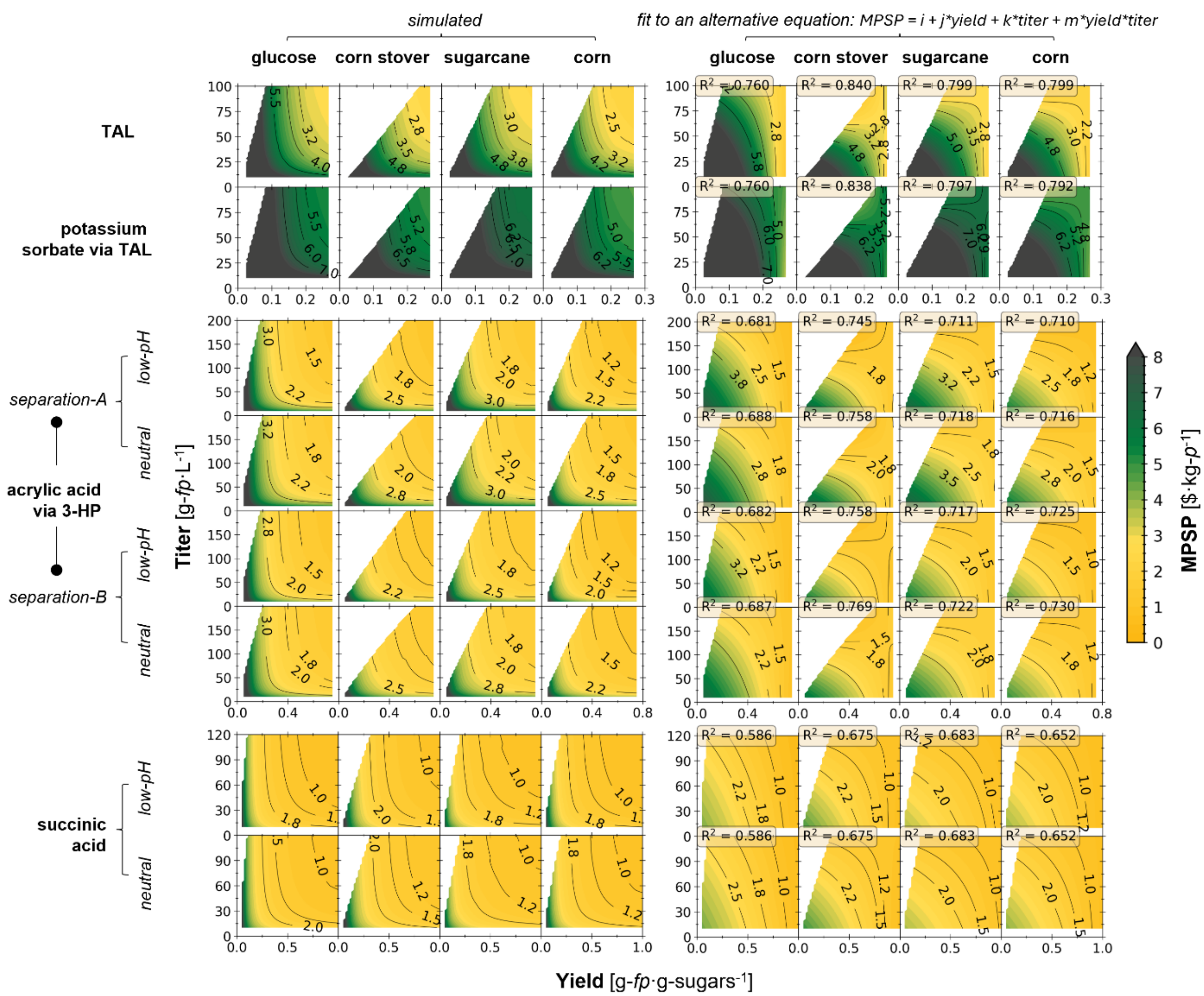

Figure S10. Minimum product selling price (MPSP) of the main biomanufacturing facility product (*p*) across fermentation yields (*x*-axes) and titers (*y*-axes) at one-fifth the baseline productivity values (i.e., at 0.024 g-*fp*·L^{-1}·h^{-1} for TAL biomanufacturing facilities, 0.066 g-*fp*·L^{-1}·h^{-1} for 3HP biomanufacturing facilities, and 0.132 g-*fp*·L^{-1}·h^{-1} for succinic acid biomanufacturing facilities). For a given point on the figure, the *x*-axis value represents the overall fermentation yield (g-*fp*·g-sugars^{-1}), the *y-axis* value represents the titer (g-*fp*·L^{-1}), and the color represents MPSP. The white region to the upper left of each plot represents infeasible yield-titer combinations (described in Section S1.1 of the SI). Panels in the first four columns show results from simulation and evaluation of biomanufacturing facilities at 2500 fermentation yield-titer combinations each, while

panels in **the last four columns show results from fitting to an alternative equation (*MPSP = i + j*yield + k*titer + m*yield*titer*, where *i*, *j*, *k*, and *m* are empirical coefficients)**. Rows represent biomanufacturing facilities producing TAL (*TAL*), potassium sorbate via TAL (*potassium sorbate via TAL*), acrylic acid via 3HP (*acrylic acid via 3HP*) under low-pH (*low-pH*) and neutral fermentation regimes (*neutral*) with separation process A (*separation-A*) or B (*separation-B*), and succinic acid (*succinic acid*) under low-pH (*low-pH*) and neutral fermentation regimes (*neutral*). Columns represent biomanufacturing facilities accepting dextrose monohydrate (*glucose*), sugarcane (*sugarcane*), corn stover (*corn stover*), and corn (*corn*) as the feedstock (all biomanufacturing facility configurations are described in Figure 5).

3.2.2. *The explanation of the coefficients is not easily understood as presented, when they could be summarized as the sale price of the fermentation product, the cost of feedstock, the cost of processing the fermentation broth, and a complex variable that incorporates cost of processing the feedstock and broth. The proposed equation XI is just a simple cost formula that is dependent on material pricing and is independent of the inputs relevant for capital and operational expenditures. While the authors present their approach as novel, it appears to align with established economic principles (cost of product = cost of raw materials + cost of processing). Clarifying how this work extends or differentiates from existing frameworks would strengthen the contribution. Are the authors unintentionally overcomplicating a simple and well-known approach for process costing?*

Response:
Thank you for this comment. The explanation of coefficients the Reviewer suggests – the sale price of the fermentation product, the cost of feedstock, the cost of processing the fermentation broth, and a complex variable that incorporates cost of processing the feedstock and broth – is intuitive, but we find from our uncertainty and sensitivity analyses that this explanation does not capture the complexities inherent to biomanufacturing flowsheets. For example, coefficient *b'* (name changed to k_0 in revisions) does not only represent the cost of the feedstock, but rather all costs that are independent of fermentation yield and titer. These include the cost of treating waste fractions of the feedstock as well as the energy recovered during anaerobic digestion of those fractions during wastewater treatment. Similarly, coefficient *c'* (name changed to k_V in revisions) does not only represent the cost of processing the broth in downstream separations, but also the cost of target-loading nutrient additives to the fermentation medium (e.g., corn steep liquor for nitrogen). These coefficient explanations were detailed in the **Results** under *Uncertainties and general drivers influencing the relationship between fermentation performance and system cost*. However, we agree with the reviewer that these physical interpretations of the equation coefficients could be better highlighted. We have renamed the sub-section *Uncertainties and general drivers influencing the relationship between fermentation performance and system cost* of the **Results** to *Physical interpretation of equation coefficients and cost drivers under uncertainty* and have revised the discussion of coefficients in the **Results** and **Conclusions** sections accordingly. Note we changed the symbols for the four coefficients from a', b', c', and d' previously to k_Y, k_0, k_V, and k_T, respectively, across the manuscript and SI to better highlight the physical interpretations discussed in the text.

The Reviewer correctly points out that the cost of a product equals the sum of the cost of raw materials and the cost of processing, in addition to capital and financing expenses and the discounted time value of money. Indeed, that equality underlies all TEA calculations and can help characterize the influence of contextual parameters (e.g., raw material purchase prices, co-product selling prices). However, that equality on its own is insufficient to predict how the system

cost is actually shaped by design and technological parameters (e.g., fermentation titer, rate, yield, pH control) as these parameters influence fundamentally different material and energy balances that shape cost through complex thermodynamic interactions that ripple through the system, necessitating thousands of simulations for each new biomanufacturing configuration. Note this work performs hundreds of thousands of simulations and evaluations (by TEA) that include capital, financing, and operating expenses (not only material cost) to estimate the minimum product selling price (MPSP) at a given internal rate of return, and the developed unifying equation has a high goodness of fit to these results fitting only four equation coefficients.

The novelty of this work is in: (i) performing hundreds of thousands of rigorous, facility-scale simulations for 32 distinct configurations under uncertainty to reveal which subsets of system costs are influenced by fermentation titer, rate, and yield (TRY); (ii) extracting a simple, generalizable equation that robustly captures the relationship between fermentation TRY and system cost in a way that transcends configurations (R^2 of 0.992–1.000 across hundreds of thousands of simulations for 32 distinct configurations); (iii) revealing generalizable insight into how uncertainties in feedstock, fermentation, separation, catalytic upgrading, and wastewater resource recovery interact to influence the *relationship* between fermentation TRY and system cost (i.e., explicitly associating design, technological, and contextual parameters with equation coefficients); and (iv) validating the developed equation with external studies that simulated alternative biomanufacturing configurations using different process simulators (Aspen Plus, SuperPro Designer) and different bioproducts and processes (1,3-propanediol under aerobic conditions, 1,3-propanediol under microaerobic conditions, and isobutanol). The developed equation can be used by fermentation researchers to rapidly assess the implications of alternative values of fermentation titer, productivity, and yield, without having to perform thousands of new simulations for each new biomanufacturing configuration and/or product in the future. The insights generated have significant implications for biomanufacturing process design methodologies and to prioritize biomanufacturing research, development, and deployment directions.

To the best of the authors' knowledge, no previous work has been able to perform rigorous uncertainty and sensitivity analyses across such a wide design space, produce a simple, generalizable relationship between MPSP and fermentation TRY with such a high goodness of fit, or generate the widely applicable design and technological insights discussed in this work. We agree with the Reviewer that this novelty could be better highlighted in the text and have accordingly updated the text throughout (in response to all comments) and revised the **Conclusions** section to explicitly emphasize these points.

Revision:

Results:

[Lines 301–431]

Physical interpretation of equation coefficients and cost drivers under uncertainty
[…]. Values of coefficient k_Y (the facility's yield-dependent burden coefficient, which influences costs that increase linearly with fermentation yield, but not titer; **Equation XII**) were consistently higher for neutral fermentation regimes than for low-pH fermentation regimes. For example, […]. Therefore, fermentation regime (neutral vs. low-pH), catalytic upgrading, and the design of separations downstream of any initial separation of water from fermentation broths (to a target concentration of *fp*) were key drivers of system cost but did not influence the relationship between fermentation performance and system cost (governed by coefficient k_Y; **Equation XII**).

Values of coefficient k_0 (the facility's TRY-invariant burden coefficient, which influences costs that are independent of fermentation yield and titer; **Equation XII**) were very similar for biomanufacturing facilities accepting the same feedstock. For example, k_0 […]. Therefore, feedstock unit price, the recoverable energy in the waste fractions of the feedstock and fermentation broth, and the efficiency of energy recovery in WWRR and CHP were key drivers influencing the relationship between fermentation yield and system cost (governed by coefficient k_0; **Equation XII**).

Coefficient k_V (the facility's broth volumetric flow-dependent burden coefficient, which influences costs that increase linearly with the ratio of fermentation yield to titer; **Equation XII**) had similar values for biomanufacturing facilities with the same separation process, regardless of the inclusion of catalytic upgrading. For example, k_V was […]. Therefore, the unit prices and loadings for fermentation nutrient sources (such as CSL) and the design of separations including and upstream of any initial separation of water from fermentation broths (to a target concentration of *fp*) were key drivers influencing the relationship between fermentation titer and system cost (governed by coefficient k_V; **Equation XII**).

Across the 24 biomanufacturing facilities evaluated under uncertainty, k_T (the facility's titer-only dependent burden coefficient, which influences costs that depend on titer but not on yield) […]. Over the evaluated theoretical fermentation space […] indicating k_T did not substantially influence the relationship between fermentation performance and system cost.

Conclusions:

[Lines 607–667]

In this study, we proposed a simple, physically interpretable mathematical equation (**Equation XII**; further simplified form shown in **Equation IV**) that robustly captures the relationship between fermentation performance (titer, rate or productivity, and yield, TRY) and system cost (R^2 of 0.992−1.000) under diverse assumptions for biomanufacturing facility design decisions, technological performance, and contextual parameters (32 distinct biomanufacturing feedstock-to-product configurations simulated under uncertainty and across 240,000 combinations of fermentation TRY using BioSTEAM), and validated it using TEA results from external studies that explored six distinct biomanufacturing feedstock-to-product configurations using Aspen Plus and SuperPro Designer (R^2 of 0.954−1.000 on results from external studies). Further, we simulated 24 biomanufacturing configurations under uncertainty at each of 25 yield-titer combinations for a total of 600,000 Monte Carlo simulations to evaluate how uncertain design, technological, and contextual parameters influence the relationship between fermentation TRY and system cost. To the best of the authors' knowledge, no previous work has suggested a mathematical equation that can capture the relationship between fermentation TRY and system cost to this degree of robustness across the vast design and thermodynamic simulation landscapes evaluated in this work, nor performed rigorous uncertainty analyses to generate widely applicable insights of the process drivers of this relationship as presented in this work.

In exploring the physical interpretations of the coefficients of the proposed mathematical equation by simulating biomanufacturing facilities under uncertainty (600,000 Monte Carlo simulations), we revealed key drivers and non-drivers of the relationship between fermentation TRY and system cost. By interpreting design, technological, and contextual drivers of the facility's yield-dependent burden coefficient k_Y, we found that while the fermentation regime (neutral vs. low pH) and the inclusion of catalytic upgrading influenced system cost, they did not substantially alter how fermentation TRY influenced system cost. Similarly, costs associated with the separation process equipment farther downstream (specifically, downstream of separation equipment that removed water from the fermentation broth to achieve a target concentration)

influenced system cost, but did not substantially alter the relationship between fermentation performance and system cost. Further, by interpreting the facility's TRY-invariant burden coefficient k_0, we found the choice of feedstock and the energy content of the waste fractions of the feedstock and fermentation broth substantially affected the influence of fermentation yield on system cost—more expensive feedstocks and lower total recoverable energy in wastes (by WWRR and CHP) amplified this influence. Finally, by interpreting the facility's broth volumetric flow-dependent burden coefficient k_V, we found costs associated with the separation process equipment immediately downstream of fermentation (specifically, including and upstream of equipment that first removed water from the fermentation broth to achieve a target concentration rather than a target amount of water removal) substantially affected the influence of titer on system cost—more expensive separations including and upstream of target concentration-based water removal amplified this influence. Similarly, an increase in costs associated with non-sugar, fixed-concentration nutrient additives to the fermentation medium (e.g., to satisfy nitrogen and phosphorus requirements) increased the influence of titer on system cost.

The analytical framework developed in this work—performing local sensitivity analyses across vast theoretical technological spaces to uncover underlying equations, followed by rigorous uncertainty and global sensitivity analyses to deepen an understanding of the relationships described by those equations—may be used to reveal broadly applicable recommendations to advance new process design paradigms that maximize the benefit from anticipated technological improvements. For example, with several alternative process flowsheets that result in similar minimum product selling prices, it may be prudent for a process design practitioner to compare values for k_V across flowsheets and select the flowsheet associated with the larger k_V term (e.g., due to costlier separations including and prior to initial water removal, or costlier fermentation nutrient media components) if future improvements to fermentation titer are anticipated. Similarly, it may be prudent to select the flowsheet associated with the larger k_0 term (e.g., from costlier feedstocks, or from lower recoverable energy in the waste fractions diverted to wastewater resource recovery) if future improvements to fermentation yield are anticipated. […]

Further, the developed equation is explicit in MPSP and could therefore be used in high-throughput paradigms for early-stage research to estimate MPSP analytically for low computational cost across vast fermentation TRY spaces; i.e., by simply substituting the TRY values of interest into the equation without requiring hundreds of thousands of computational cost-intensive chemical process simulations. For example, the equation may be leveraged to design holistic objective functions or guidance metrics (e.g., RI_{MPSP}; **Equation XVIII**) for platforms that aim to engineer optimal metabolic pathways in microbes through automated prototyping,[22–25] especially alongside potential future efforts to develop artificial intelligence-based approaches to predict equation coefficient values from feedstock, product, and process specifications. […].

3.2.3. *The utility of any model is the ability to predict outcomes. Can the authors provide any context about the predictive capability of their process models? How do their correlated outcomes compare with any physical systems? Are there other specific advantages of using the proposed reduced order model over traditional process and cost models? As mentioned above, does the outcome of the coefficient values elucidate any key constraints to consider for the process design or economic drivers for a particular process?*

Response:
We thank the Reviewer for this comment. We have revised the **Conclusions** to highlight the utility of this work, the reduced computational cost of the develop equation relative to performing

hundreds of thousands of chemical process simulations, and the physical interpretation of equation coefficients, as detailed in our response to **Comment 3.2.2**. Further, although *n*th plant TEAs are designed to use industrial data for individual equipment cost scaling and rigorous thermodynamic equilibrium estimation methods, there are typically no full-facility industrial cost data available for comparisons (as facilities producing those chemicals are typically pioneer facilities; i.e., <*n*th plant, or in some cases have not even been deployed yet). To highlight this, we have added a section to the **Results** named *Key considerations for equation applicability* to describe key limitations in the scope of this work. This discussion includes conditions where *n*th plant TEAs (and therefore the equation that was developed using *n*th plant TEAs performed in this work and validated on results from externally performed *n*th plant TEAs) may not comprehensively capture industrial costs.

Revision:

Results:

Key considerations for equation applicability

Please see the new text in this section provided in response to Comment 1.2.2. This includes 4 new paragraphs to address these points.

3.2.4. *Figure 1: Have you considered alternative visualizations to better discern the goodness of fit and where the model may fail to accurately match results? For example, what about a surface plot that shows % difference between the detailed model and the reduced-order fit model? Doing so would identify 'hotspots' where the reduced order model fails to accurately represent details. Figure S5 is a good example.*

Response:
Thank you for this suggestion. We agree with the Reviewer that a two-dimensional residual plot as described by the Reviewer would be a valuable addition to this work and have accordingly revised the **Results** section and added a new figure (**Figure S8**) to the **Supplementary Information** as below.

Revision:

Results:

[Lines 214–235]

Generalized trends in how fermentation performance influences system cost

[...] Note that despite the generally high goodness of fit (R^2 of 0.992–1.000 across 32 biomanufacturing configurations; **Figure 1** and **Figures S1** and **S2** in the SI), hotspots did appear where the developed equation did not capture the relationship between fermentation TRY and MPSP as robustly (**Figure S8** in the SI). For simulations across titer-yield spaces at baseline productivity values (**Figure 1**), the absolute MPSP error (calculated as fractional absolute residual values; i.e., the ratio of the absolute residual MPSP value between the simulation and fitting results to the MPSP value from simulation) was 0.9% (median) with a range of 0.0–3.4% [5th–95th percentiles, hereafter in brackets] for biomanufacturing facilities producing TAL or potassium sorbate via TAL, 0.8% [0.0–2.7%] for biomanufacturing facilities producing acrylic acid via 3HP, and 1.0% [0.0%–2.8%] for biomanufacturing facilities producing succinic acid. The developed

equation was not substantially biased toward either under-predicting or over-predicting the MPSP, with an MPSP error (calculated as signed fractional residual values rather than absolute values) of 0.0% [-2.4 to +3.3%] for biomanufacturing facilities producing TAL or potassium sorbate via TAL, 0.0% [-2.3% to +2.1%] for biomanufacturing facilities producing acrylic acid via 3HP, and 0.0% [-2.5% to +2.1%] for biomanufacturing facilities producing succinic acid (**Figure S8** in the SI). Notably, outlier absolute errors of 10% or higher were found at sixteen points among the 80000 points simulated, specifically for biomanufacturing facilities producing TAL or potassium sorbate via TAL from glucose, specifically. All sixteen points were at four combinations of high values for titer (~100 $g \cdot L^{-1}$) and low values for yield (~0.05 $g \cdot g^{-1}$ glucose), indicating the error may be due to changes in the optimal configurations of heat exchanger networks designed at those points due to higher evaporation heating duties, resulting in step changes in system heating utility demands at those four points relative to other areas in the evaluated titer-yield spaces.

Supplementary Information:

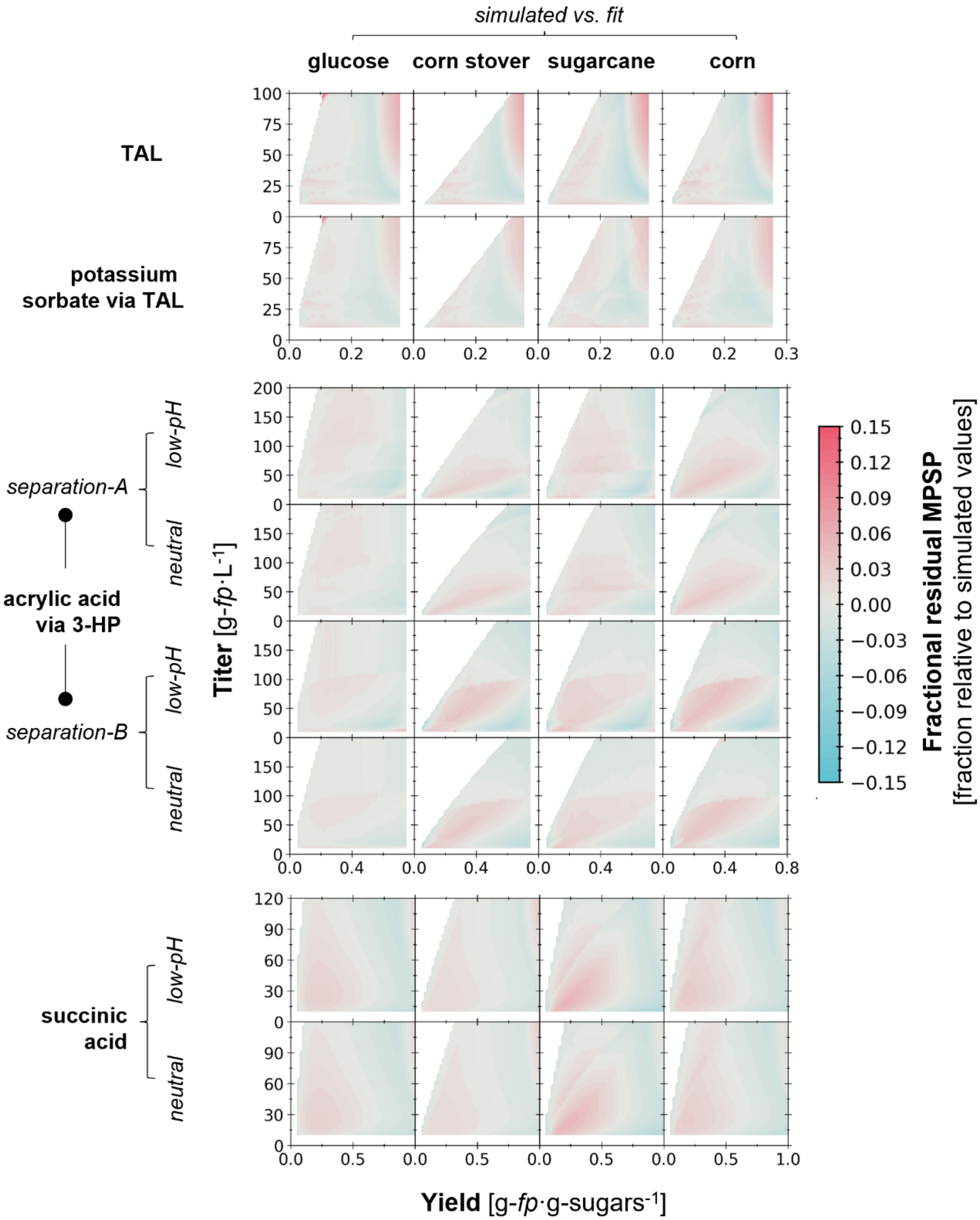

Figure S8. MPSP residuals represented as fractional residual values calculated as the difference between the *simulated* and *fit* MPSP values divided by the *simulated* MPSP value (*simulated* and *fit* MPSP values shown in Figure 1) across fermentation titer and yield at baseline productivity values (0.12 g-*fp*·L^{-1}·h^{-1} for TAL biomanufacturing facilities, 0.28 g-*fp*·L^{-1}·h^{-1} for 3HP biomanufacturing facilities, and 0.66 g-*fp*·L^{-1}·h^{-1} for succinic acid biomanufacturing facilities) for 32 biomanufacturing facilities.

3.2.5. *R-squared values in figure S2 can be difficult to read*

Response:

Thank you for this comment. The R^2 values were indeed overlapping in places with the contour lines and labels due to order-of-drawing issues, and the transparency of the box containing R^2 values was a little too high to be quickly readable. We have resolved these formatting issues accordingly and updated **Figures 1** in the **Results** as well as **Figures S2** and **S3** in the **Supplementary Information**.

Revision:

Results:

Generalized trends in how fermentation performance influences system cost

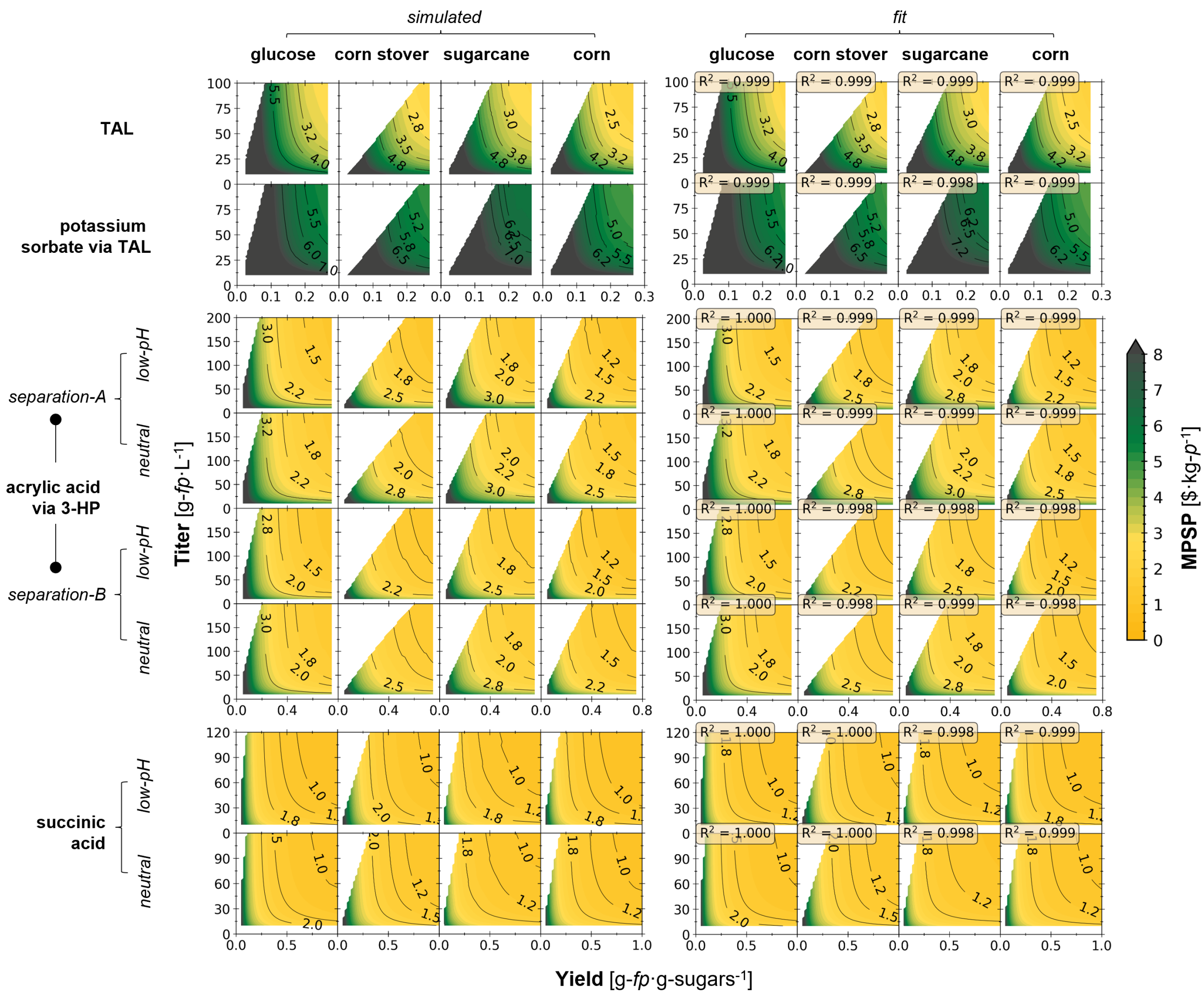

Figure 1. Minimum product selling price (MPSP) of […].

Supplementary Information:

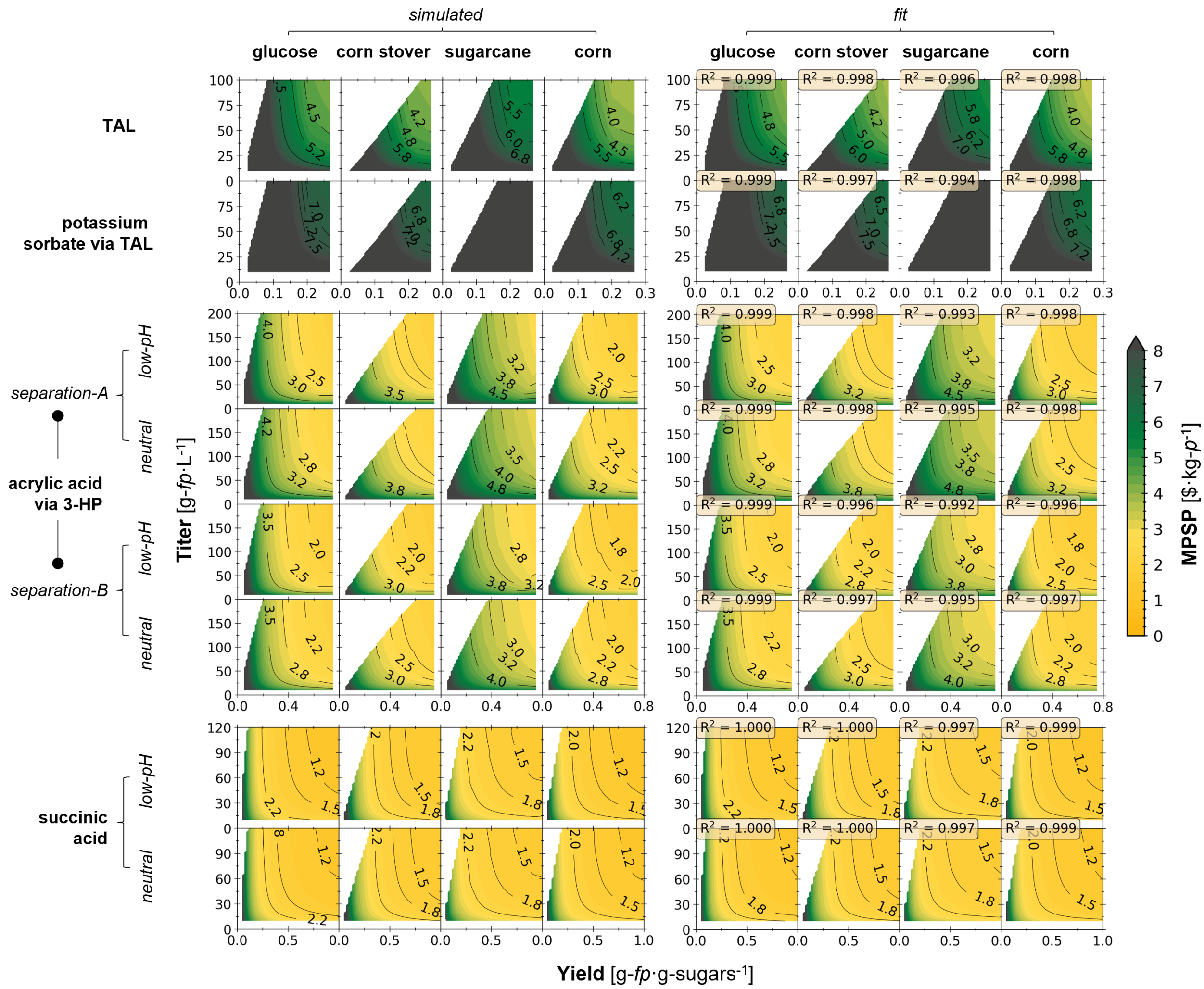

Figure S1. Minimum product selling price (MPSP) of […].

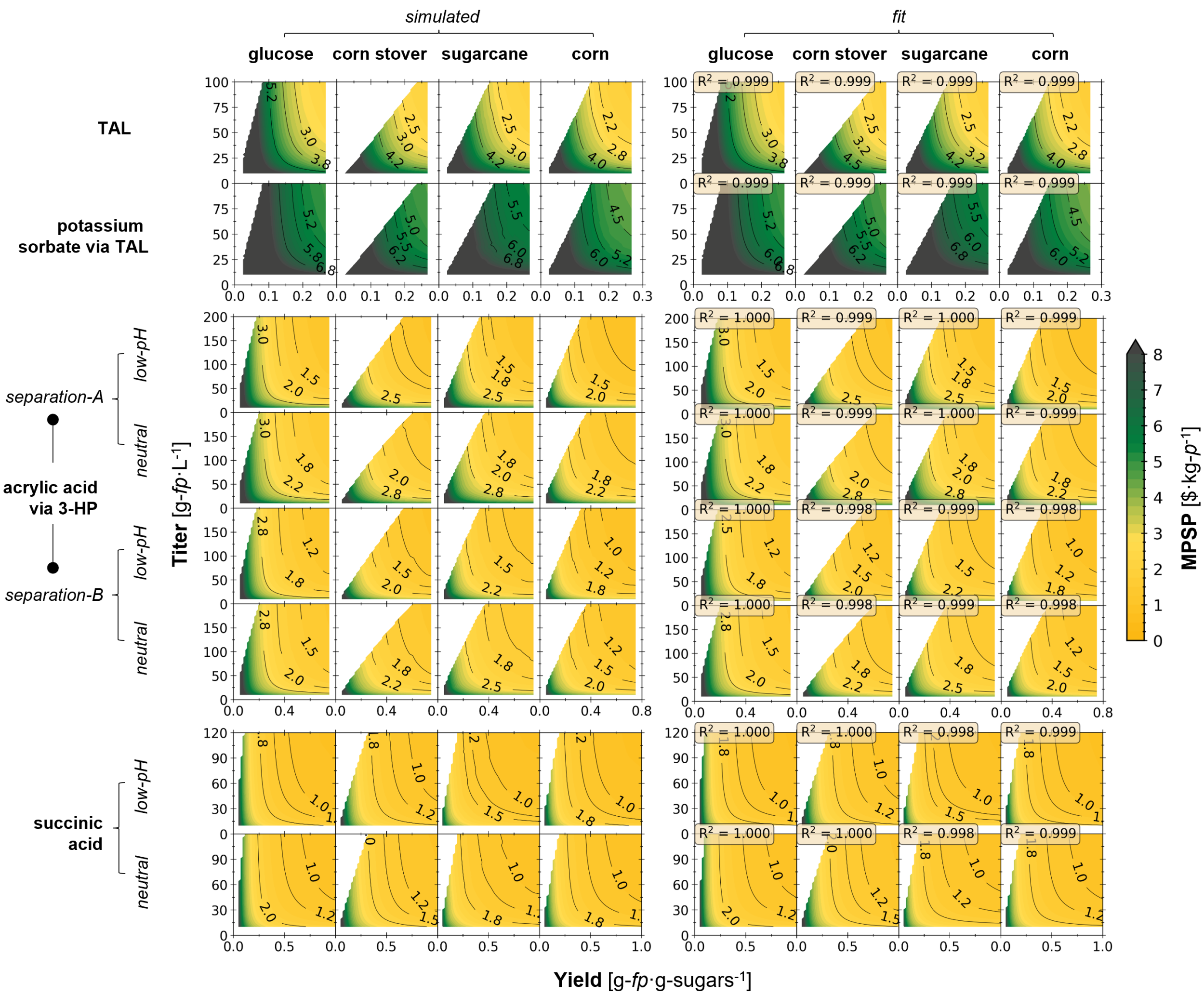

Figure S2. Minimum product selling price (MPSP) of […].

<u>3.2.6.</u> *Line 316: What is a boiler prism? I don't think this is common terminology that will be easily understood, I don't really know what it is.*

Response:
We agree this could be clearer. We have updated the text to replace "boiler prism" with "boiler combustion chamber" (i.e., the space within the boiler in which gases and/or solid wastes are burned to produce heat) as the latter is the more commonly used term.

Revision:

Results:

[Lines 347–350]

Physical interpretation of equation coefficients and cost drivers under uncertainty

[….]. Baseline values and uncertainty distributions for fermentation cell mass yields were based on reported values and assumed to be independent of fermentation yield and titer, so the costs

and revenues associated with burning centrifuged cell mass in the boiler combustion chamber to recover steam utilities did not change with fermentation yield or titer.

3.2.7. *Figure references appear to be incorrect. Examples:*

Line 142-143: "32 right-hand side panels in Figure 1 and Figures S2 and S3 in the SI"

This appears to refer to Figures S1 and S2, although Figure S3 also contains 32 right-hand side panels

S1.1 Fermentation Processes, paragraph 3: "This manifests as an infeasible region in the upper-left portion of the fermentation titer-yield space (represented by white regions in Figures 1, S2, S3, S4, S6, and S7)."

Figure S4 is a Sankey diagram, Figure S7 is a black-and-white contour plot, and Figure S1 is similar to Figure S2 but is not included in the list.

Response:
We thank the reviewer for identifying these incorrect references to figures. We have re-reviewed the manuscript and SI for these and other incorrect figure/table references and have accordingly revised the text as below.

Revision:

Results:

[Line 137] […] at each point (32 left-hand side panels in **Figure 1** and **Figures S1** and **S2** in the SI).

[Lines 140–143] […] across fermentation yield and titer (coefficient of determination, R^2 of 0.992−1.000 across 7500 yield-titer-productivity combinations each for 32 biomanufacturing facilities; 32 right-hand side panels in **Figure 1** and **Figures S1** and **S2** in the SI) […].

[Line 257] […] R^2 of 0.992−1.000; **Figure 1** and **Figures S1** and **S2** in the SI […].

[Line 467–469] For all 32 biomanufacturing facilities […] fermentation performance space (**Figure 4**).

Supplementary Information:

[*Section S1.1. Fermentation Processes*] […]. This manifests as an infeasible region in the upper-left portion of the fermentation titer-yield space (represented by white regions in **Figures 1**, **S1, S2**, **S3**, **S5**, **S6**, **S8**, **S9**, and **S10**).

3.2.8. *In general, the writing should be reviewed for brevity and clarity. Examples:*

Line 120:

"… and two catalytic upgrading processes (3HP [...]; TAL [...]; all biomanufacturing facility configurations are shown in Figure 5 [...])."

The final phrase should not be included in the list of catalytic upgrading processes. Instead, this could be "… and two catalytic upgrading processes (3HP [...]; TAL [...]). All biomanufacturing facility configurations are shown in Figure 5 [...]."

Response:
Thank you for your suggested edits for brevity and clarity. We have re-reviewed the manuscript and SI for these and other lengthy sentences and have accordingly revised the text below (in addition to the edits presented in red throughout this response document).

Revision:
Results:
[Lines 120–123] […] and two catalytic upgrading processes (3HP [...]; TAL [...]). All biomanufacturing facility configurations are shown in **Figure 5** [...].

[Lines 499–505] […]. For biomanufacturing facilities with the same fermentation product and feedstock, the […] (e.g., […]; **Figure 4**). This was consistent with k_0 and k_T remaining relatively unchanged and […] (**Figure 3B,C,D**).

[Lines 251–257] […]. For biomanufacturing facilities with […] productivity (**Figures S4** and **S7B** in the SI). Thus, **Equation IV** […] facilities (R^2 of 0.992−1.000; **Figure 1** and **Figures S1** and **S2** in the SI).

[Lines 322–328] […]. From the parameters included in the uncertainty analyses that […] relative to fermentation yield. This was consistent with […] as titer increased (**Figure S4C** in the SI).

[Lines 359–364] […]. Finally, for all 24 biomanufacturing facilities […] product storage time (**Table S5** in the SI). This was consistent with […] and titer (**Figure S4** in the SI).

3.2.9. *Line 534:*

"[...] four main (inside battery limits) processes (feedstock pretreatment [...] and catalytic upgrading) with outside-battery wastewater resource recovery, [...] and miscellaneous facilities (an overview of biomanufacturing facility processes [...] is provided in Figure 5 and in Table S1 in the SI)."

This sentence structure implies that the final parentheses is clarifying the term "miscellaneous facilities". To remove ambiguity, this could be written as "… and miscellaneous facilities. An overview of biomanufacturing facility processes [...] is provided in Figure 5 and in Table S1 in the SI."

Response:
Thank you for this suggestion to improve clarity. We have accordingly revised the text as below.

Revision:
Methods:

[Lines 685–687] […] and miscellaneous facilities. An overview of biomanufacturing facility processes and the configurations of the 32 biomanufacturing facilities evaluated in this study is provided in **Figure 5** and in **Table S1** in the SI.

3.2.10. *Line 554-569:*

The following adjacent sentences appear to repeat the same information:

"Models for all process alternatives were from previous studies; specifically: (i) corn dry grind; (ii) sugarcane juicing and clarification; [...] (xii) 3HP catalytic upgrading to acrylic acid. 13 All listed models are available open-source in the online repositories.30–33"

"All process models [...] were developed in previous studies: namely, for feedstock pretreatment and saccharification (corn dry grind process, sugarcane juicing and clarification, [...]; and for catalytic upgrading of fermentation products (upgrading 3HP to acrylic acid [...])."

These sentences could be combined. If they convey different information, the differences should be clarified.

Response:
Thank you for identifying these adjacent sentences that repeated information unnecessarily. We have accordingly revised the text to keep only one of these sentences (shown below).

Revision:
Methods:
[Lines 703–717] […]. Models for all process alternatives were from previous studies; specifically: (i) corn dry grind; (ii) sugarcane juicing and clarification; (iii) corn stover pretreatment and saccharification;[1] (iv) fermentative TAL production;[5] (v) fermentative 3HP production;[17] (vi) fermentative succinic acid production;[3] (vii) TAL separation by crystallization;[5] (viii) 3HP separation by adsorption, ion exchange, and evaporation (separation-A);[16] (ix) 3HP separation by hexanol-based solvent extraction (separation-B);[4] (x) succinic acid separation by evaporation and crystallization;[3] (xi) TAL catalytic upgrading to potassium sorbate;[6] and (xii) 3HP catalytic upgrading to acrylic acid.[4] All listed models are available open-source in the online repositories.[18–21]

[Adjacent sentence with repeating information removed.]

3.2.11. *Line 554-569:*

Figure 4 caption, lines 451 – 457:

"Rows represent biomanufacturing facilities producing TAL as the main product (TAL), biomanufacturing facilities producing potassium sorbate via TAL [...], and biomanufacturing facilities producing succinic acid (succinic acid) under low-pH (low-pH) and neutral fermentation regimes (neutral). Columns represent biomanufacturing facilities accepting dextrose monohydrate 458 (glucose), [...], and corn (corn) as the feedstock (all [...] described in Figure 5)." (95 words)

This could be reduced to

"Rows represent biomanufacturing facilities producing TAL, potassium sorbate via TAL, acrylic acid via 3HP under low-pH and neutral fermentation regimes and with separation process A or B, and succinic acid under low-pH and neutral fermentation regimes. Columns represent biomanufacturing facilities accepting glucose, sugarcane, corn stover, and corn as the feedstock." (50 words)

Response:
Thank you for identifying this suggestion for brevity. We have accordingly revised the captions for **Figures 1** and **4** in the **Results** as well as **Figures S1**, **S2**, **S9**, and **S10** in the **SI** to shorten the text while keeping specific labels for clarity as below.

Revision:

Results:

[**Figure 1 caption**] […]. Rows represent biomanufacturing facilities producing TAL (*TAL*), potassium sorbate via TAL (*potassium sorbate via TAL*), acrylic acid via 3HP (*acrylic acid via 3HP*) under low-pH (*low-pH*) and neutral fermentation regimes (*neutral*) with separation process A (*separation-A*)[16] or B (*separation-B*)[4], and succinic acid (*succinic acid*) under low-pH (*low-pH*) and neutral fermentation regimes (*neutral*). Columns represent biomanufacturing facilities accepting dextrose monohydrate (*glucose*), sugarcane (*sugarcane*), corn stover (*corn stover*), and corn (*corn*) as the feedstock. All biomanufacturing facility configurations are described in **Figure 5**.

[**Figure 4 caption**] […]. Rows represent biomanufacturing facilities producing TAL (*TAL*), potassium sorbate via TAL (*potassium sorbate via TAL*), acrylic acid via 3HP (*acrylic acid via 3HP*) under low-pH (*low-pH*) and neutral fermentation regimes (*neutral*) with separation process A (*separation-A*)[16] or B (*separation-B*)[4], and succinic acid (*succinic acid*) under low-pH (*low-pH*) and neutral fermentation regimes (*neutral*). Columns represent biomanufacturing facilities accepting dextrose monohydrate (*glucose*), sugarcane (*sugarcane*), corn stover (*corn stover*), and corn (*corn*) as the feedstock. All biomanufacturing facility configurations are described in **Figure 5**.

Supplementary Information:

[**Figure S1 caption**] […]. Rows represent biomanufacturing facilities producing TAL (*TAL*), potassium sorbate via TAL (*potassium sorbate via TAL*), acrylic acid via 3HP (*acrylic acid via 3HP*) under low-pH (*low-pH*) and neutral fermentation regimes (*neutral*) with separation process A (*separation-A*)[16] or B (*separation-B*)[4], and succinic acid (*succinic acid*) under low-pH (*low-pH*) and neutral fermentation regimes (*neutral*). Columns represent biomanufacturing facilities accepting dextrose monohydrate (*glucose*), sugarcane (*sugarcane*), corn stover (*corn stover*), and corn (*corn*) as the feedstock. All biomanufacturing facility configurations are described in **Figure 5**.

[**Figure S2 caption**] […]. Rows represent biomanufacturing facilities producing TAL (*TAL*), potassium sorbate via TAL (*potassium sorbate via TAL*), acrylic acid via 3HP (*acrylic acid via 3HP*) under low-pH (*low-pH*) and neutral fermentation regimes (*neutral*) with separation process A (*separation-A*)[16] or B (*separation-B*)[4], and succinic acid (*succinic acid*) under low-pH (*low-pH*) and neutral fermentation regimes (*neutral*). Columns represent biomanufacturing facilities accepting dextrose monohydrate (*glucose*), sugarcane (*sugarcane*), corn stover (*corn stover*),

and corn (*corn*) as the feedstock. All biomanufacturing facility configurations are described in **Figure 5**.

[**Figure S9 caption**] […]. Rows represent biomanufacturing facilities producing TAL (*TAL*), potassium sorbate via TAL (*potassium sorbate via TAL*), acrylic acid via 3HP (*acrylic acid via 3HP*) under low-pH (*low-pH*) and neutral fermentation regimes (*neutral*) with separation process A (*separation-A*)[16] or B (*separation-B*)[4], and succinic acid (*succinic acid*) under low-pH (*low-pH*) and neutral fermentation regimes (*neutral*). Columns represent biomanufacturing facilities accepting dextrose monohydrate (*glucose*), sugarcane (*sugarcane*), corn stover (*corn stover*), and corn (*corn*) as the feedstock. All biomanufacturing facility configurations are described in **Figure 5**.

[**Figure S10 caption**] […]. Rows represent biomanufacturing facilities producing TAL (*TAL*), potassium sorbate via TAL (*potassium sorbate via TAL*), acrylic acid via 3HP (*acrylic acid via 3HP*) under low-pH (*low-pH*) and neutral fermentation regimes (*neutral*) with separation process A (*separation-A*)[16] or B (*separation-B*)[4], and succinic acid (*succinic acid*) under low-pH (*low-pH*) and neutral fermentation regimes (*neutral*). Columns represent biomanufacturing facilities accepting dextrose monohydrate (*glucose*), sugarcane (*sugarcane*), corn stover (*corn stover*), and corn (*corn*) as the feedstock. All biomanufacturing facility configurations are described in **Figure 5**.

3.2.12. *In lines 618 and 629, providing values taken from literature or github could help readers understand the assumptions used in the analysis. This was done in lines 149-152.*

Response:
We thank the reviewer for these suggestions. For the sentence beginning on Line 618 (now Line 760 after revisions in the clean, "no tracked edits" version of the manuscript) discusses detailed results data for each of the simulations and TEAs performed in this work. These detailed data span tables with thousands of rows and columns. Traditional, static approaches to TEA are usually limited to one or a few simulations, allowing authors to include these data in the manuscript or supporting information. However, our approach includes uncertainty and sensitivity analyses (>840,000 simulations) across a wide design landscape (32 distinct biomanufacturing configurations). As such, including these detailed data in the **Supplementary Information** (SI) – even if only for the 32 baseline simulations in this work – would result in large, unwieldy SI documents far outsizing the main text. Therefore, although we include detailed lists of assumptions for baseline values and probability distributions for parameters included in the uncertainty analyses (**Table S3**) as well as results for sensitivity analyses (**Tables S4–S7**) in the SI, we make these detailed results data available as human-readable worksheets in the online repositories rather than the SI, making sure to provide clear references for these repositories. This approach is consistent with other manuscripts we have published in *Nature Communications*.[3,17,26,27]

However, we agree with the Reviewer that the sentence beginning on Line 629 (now Line 765 after revisions in the clean version of the manuscript) could mention values for the reader's convenience, and we have accordingly revised the **Methods** section as below.

Revision:

Methods:

[Lines 765–780]

System analyses under uncertainty

Uncertainty and Sensitivity Analyses

[…]. For each of the 32 biomanufacturing facilities evaluated in this work (**Figure 5**), 2500 potential combinations of fermentation yield and titer were evaluated at 3 alternative values of fermentation productivity (20%, 100%, and 500% of each of the reported baseline productivity values of 0.12 g-*fp*·L^{-1}·h^{-1} for TAL, 0.28 g-*fp*·L^{-1}·h^{-1} for 3HP, and 0.66 g-*fp*·L^{-1}·h^{-1} for succinic acid) for a total of 240,000 simulations.[3–5] For fermentation yield, the lower bounds for evaluation were set to ~5% of the theoretical maximum yields to ensure non-zero product recovery (the theoretical maximum yields were 0.467 g·g^{-1} glucose-eq. for TAL, 1.000 g·g^{-1} glucose-eq. for 3HP, and 1.31 g·g^{-1} glucose-eq. for succinic acid) and the upper bounds were each set to the maximum value at which: (i) the total glucose conversion (alongside other reactions for microbial cell mass formation, co-product formation, and respiration, based on their reported baseline yields[3–5]) did not exceed 100%; and (ii) the fermentation product yield did not exceed the theoretical maximum yield. For fermentation titer, the upper bounds for evaluation were set to 100 g·L^{-1} for TAL (note that the utilized process models adequately characterize the implications of the low solubility of TAL in water[5]), 200 g·L^{-1} for 3HP, and 120 g·L^{-1} for succinic acid based on the bounds used for evaluation in previous studies.[3–5]

REVIEWER 4

4.1. General Comments:

I co-reviewed this manuscript with one of the reviewers who provided the listed reports. This is part of the Nature Communications initiative to facilitate training in peer review and to provide appropriate recognition for Early Career Researchers who co-review manuscripts.

Response:
We thank the Reviewer for their assessment of the work and for their constructive comments. We hope this response has adequately addressed any concerns the Reviewer may have had.

OTHER CHANGES

Revisions:

Introduction:

[Lines 101–102] […]. Further, we perform 600,000 Monte Carlo simulations across theoretical fermentation spaces for 24 biomanufacturing facilities.

Results:

[Line 343] […]. In addition, k_0 was sensitive to feedstock capacity […].

[**Figure 3** caption]. Uncertainties (box-and-whisker plots) in the coefficients (A) a', (B) b', (C) c', and (D) d' in **Equation XI** from fitting to results from 1000 Monte Carlo simulations at each of 25 fermentation yield-titer combinations at baseline productivity values for each of 24 biomanufacturing facilities (i.e., 600000 Monte Carlo simulations in total). […].

Conclusions:

[Line 670] […] systems-scale sustainability at the current state-of-technology and under potential future fermentation advancements.

Methods:

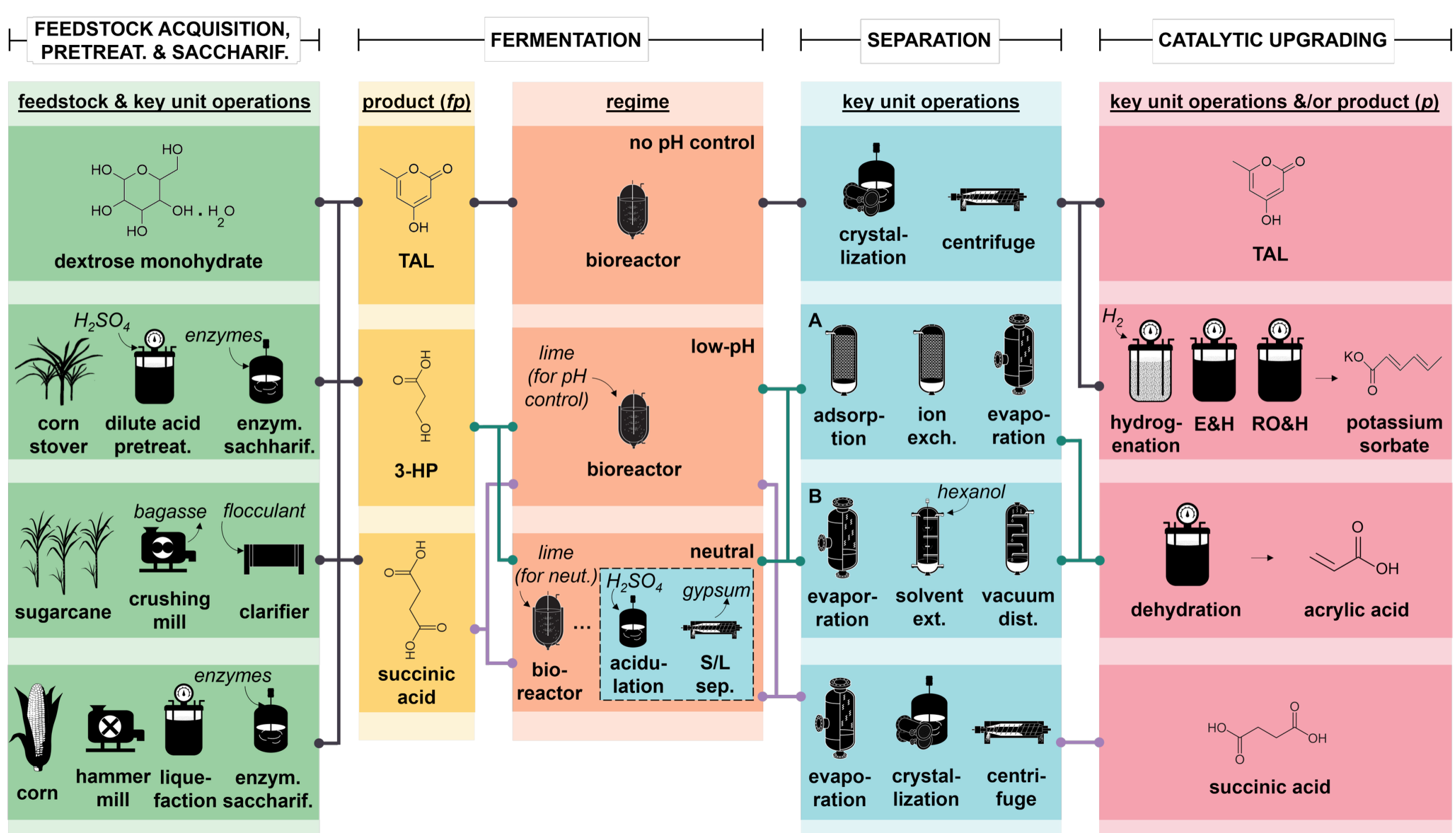

Figure 5 [edited the teal lines for 3HP and purple lines for succinic acid for clarity.] Overview of the 32 biomanufacturing facility configurations evaluated in this study formed by combinations of discrete design decisions. […]

References for the Response document

1. Cortes-Peña, Y., Kumar, D., Singh, V. & Guest, J. S. BioSTEAM: A Fast and Flexible Platform for the Design, Simulation, and Techno-Economic Analysis of Biorefineries under Uncertainty. *ACS Sustain. Chem. Eng.* **8**, 3302–3310 (2020).
2. Cortés-Peña, Y. Thermosteam: BioSTEAM's Premier Thermodynamic Engine. *J. Open Source Softw.* **5**, 2814 (2020).
3. Tran, V. G. *et al.* An end-to-end pipeline for succinic acid production at an industrially relevant scale using Issatchenkia orientalis. *Nat. Commun.* **14**, 6152 (2023).
4. Bhagwat, S. S. *et al.* Sustainable Production of Acrylic Acid via 3-Hydroxypropionic Acid from Lignocellulosic Biomass. *ACS Sustain. Chem. Eng.* **9**, 16659–16669 (2021).
5. Bhagwat, S. S. *et al.* Sustainable Triacetic Acid Lactone Production from Sugarcane and Sweet Sorghum by Fermentation and Fractional Crystallization. *ACS Sustain. Chem. Eng.* **13**, 17794–17805 (2025).
6. Kim, M. S. *et al.* Sustainable potassium sorbate production from triacetic acid lactone in food-grade solvents. *Green Chem.* 10.1039.D4GC04832F (2025) doi:10.1039/D4GC04832F.
7. U.S. EPA. Lifecycle Analysis of Greenhouse Gas Emissions under the Renewable Fuel Standard. https://www.epa.gov/renewable-fuel-standard-program/lifecycle-analysis-greenhouse-gas-emissions-under-renewable-fuel.
8. Gunukula, S., Runge, T. & Anex, R. Assessment of Biocatalytic Production Parameters to Determine Economic and Environmental Viability. *ACS Sustain. Chem. Eng.* **5**, 8119–8126 (2017).
9. Janković, T., Straathof, A. J. J. & Kiss, A. A. Advanced downstream processing of bioethanol from syngas fermentation. *Sep. Purif. Technol.* **322**, 124320 (2023).
10. Sikazwe, M. K., Louw, J. & Görgens, J. F. Microbe and bioprocess performances for sustainable production of biobased 2,3-butanediol in a sugarcane biorefinery; a technoeconomic and environmental analysis. *Clean Technol. Environ. Policy* **26**, 4505–4519 (2024).
11. Gunukula, S., Runge, T. & Anex, R. Assessment of Biocatalytic Production Parameters to Determine Economic and Environmental Viability. *ACS Sustain. Chem. Eng.* **5**, 8119–8126 (2017).
12. Nielsen, J. & Keasling, J. D. Engineering Cellular Metabolism. *Cell* **164**, 1185–1197 (2016).
13. Lodz University of Technology, Faculty of Biotechnology and Food Sciences, Institute of Fermentation Technology and Microbiology, Department of Spirit and Yeast Technology Wolczanska 171/173, PL-90-924 Lodz, Poland, Robak, K., Balcerek, M., & Lodz University of Technology, Faculty of Biotechnology and Food Sciences, Institute of Fermentation Technology and Microbiology, Department of Spirit and Yeast Technology Wolczanska 171/173, PL-90-924 Lodz, Poland. Review of Second-Generation Bioethanol Production from Residual Biomass. *Food Technol. Biotechnol.* **56**, (2018).
14. Dahiya, S. *et al.* Food waste biorefinery: Sustainable strategy for circular bioeconomy. *Bioresour. Technol.* **248**, 2–12 (2018).
15. Gallezot, P. Conversion of biomass to selected chemical products. *Chem. Soc. Rev.* **41**, 1538–1558 (2012).
16. Mahata, C.; Mishra, S. M.; Singh, V. 3-Hydroxypropionic Acid Recovery from Fermentation Broth through Novel Downstream Processing: Technoeconomic Analysis. *Bioresource Technology* **2025**. https://doi.org/10.1016/j.biortech.2026.134094.
17. Tan, S. I.; Bhagwat, S. S.; Martin, T. A.; Mishra, S. M.; Suthers, P. F.; Maranas, C. D.; Singh, V.; Guest, J. S.; Zhao, H. High Yield Production of 3-Hydroxypropionic Acid Using Issatchenkia Orientalis. *Nature Communications* **2025**. https://doi.org/10.1038/s41467-025-67621-8.
18. BioSTEAM Development Group. Modules to generate widely applicable fermentation insights. https://github.com/sarangbhagwat/fermentation_insights/tree/main (accessed 2025-08-27).

19. BioSTEAM Development Group. Triacetic acid lactone biorefineries. https://github.com/BioSTEAMDevelopmentGroup/Bioindustrial-Park/tree/master/biorefineries/TAL (accessed 2025-08-27).
20. BioSTEAM Development Group. 3-Hydroxypropionic acid biorefineries. https://github.com/BioSTEAMDevelopmentGroup/Bioindustrial-Park/tree/master/biorefineries/HP (accessed 2025-08-27).
21. BioSTEAM Development Group. Succinic acid biorefineries. https://github.com/BioSTEAMDevelopmentGroup/Bioindustrial-Park/tree/master/biorefineries/succinic (accessed 2025-08-27).
22. Radivojević, T., Costello, Z., Workman, K. & Garcia Martin, H. A machine learning Automated Recommendation Tool for synthetic biology. *Nat. Commun.* **11**, 4879 (2020).
23. HamediRad, M. *et al.* Towards a fully automated algorithm driven platform for biosystems design. *Nat. Commun.* **10**, 5150 (2019).
24. Karim, A. S. *et al.* In vitro prototyping and rapid optimization of biosynthetic enzymes for cell design. *Nat. Chem. Biol.* **16**, 912–919 (2020).
25. Zhang, J. *et al.* Combining mechanistic and machine learning models for predictive engineering and optimization of tryptophan metabolism. *Nat. Commun.* **11**, 4880 (2020).
26. Tran, V. G. *et al.* Decompartmentalization of the yeast mitochondrial metabolism to improve chemical production in Issatchenkia orientalis. *Nat. Commun.* **16**, 7110 (2025).
27. Lu, J. *et al.* Bio-based Oxalic Acid Production in Issatchenkia orientalis Enables Sustainable Rare Earth Recovery. (2025).

Response to Referees
A Unifying Equation for Fermentation Sustainability across the Titer-Rate-Yield Landscape

REVIEWER 1

1.1. General comments:

The authors have addressed this reviewer's comments.

Response:

We thank the Reviewer again for their constructive review and recommendation to accept this manuscript for publication.

1.2. Remarks on code availability:

The repository looks good. The README could be improved by including a section for readers that are not familiar with python, github, and/or biosteam. Several technical terms, like repository cloning, could benefit from links to introduction tutorials.

Response:

We thank the Reviewer for this suggestion. We have added links in the README to Python, GitHub, and BioSTEAM documentation.

Revision:

Updated the README to include links to Python, GitHub, and BioSTEAM documentation: https://github.com/sarangbhagwat/fermentation_insights/tree/main

REVIEWER 2

2.1. General comments:

The authors addressed my comments and significantly improved the manuscript. I recommend it for publishing in NC.

2.2. Remarks on code availability:

The code is available with file for installation.

Response:

We thank the Reviewer again for their constructive review and recommendation to accept this manuscript for publication.

REVIEWER 3

3.1. General comments:

Bhagwat et al. have provided a thorough revision to their manuscript. After review of their revised manuscript and rebuttal letter, the authors have reasonably addressed reviewer questions and concerns. I thank the authors for their detailed responses and thorough revision. Overall, the manuscript is acceptable for publication.

Response:

We thank the Reviewer again for their constructive review and recommendation to accept this manuscript for publication. We have addressed specific comments below.

3.2. Remarks on code availability:

Code for new figures appears to be present and functional.

3.3. Specific comments:

As noted in the first review, some text does not perfectly align with the content of certain figures. New instances are noted below for the authors to review before finalizing the publication:

3.3.1. Line 254: "r does not change substantially with fermentation yield, titer, or productivity (Figures S4 and S7B in the SI)."

Response:

We thank the Reviewer for identifying this typographical error and have accordingly revised the Manuscript.

Revision:

Line 214: "r does not change substantially with fermentation yield, titer, or productivity (Supplementary Figures 4 and 6B in the Supplementary Information)."

3.3.2. Line 375: "the total volume of the fermentation reactors were both expected to increase linearly with the ratio of fermentation yield to titer at a constant productivity (Figure S4C in the SI)"

Response:

We thank the Reviewer for identifying this typographical error and have accordingly revised the Manuscript.

Revision:

Line 325: "the total volume of the fermentation reactors were both expected to increase linearly with the ratio of fermentation yield to titer at a constant productivity (Supplementary Figure 4D in the Supplementary Information)"

3.3.3. Line 401: "none of the biomanufacturing facility flows associated with these parameters were expected to increase linearly with the ratio of fermentation yield to titer (Figure S4 in the SI)"

Response:

We thank the Reviewer for identifying this typographical error and have accordingly revised the Manuscript.

Revision:

Line 352: "none of the biomanufacturing facility flows associated with these parameters were expected to increase linearly with the ratio of fermentation yield to titer (Supplementary Figure 4D in the Supplementary Information)"

REVIEWER 4

4.1. General comments:

I co-reviewed this manuscript with one of the reviewers who provided the listed reports. This is part of the Nature Communications initiative to facilitate training in peer review and to provide appropriate recognition for Early Career Researchers who co-review manuscripts.

Response:

We thank the Reviewer again for their constructive review and recommendation to accept this manuscript for publication.

OTHER CHANGES

Revisions:

Manuscript:

Figure 3. [edited *y*-axis titles to reflect revised equation coefficient names]

[Lines 210 – 211: corrected new coefficient units] The coefficients k_Y, k_0, k_V, and k_T—in units of $·kg-*fp*$^{-1}$, $·kg-sugars^{-1}, $·m^{-3}-broth, and $·kg-*fp*·m^{-3}-broth·kg-sugars^{-1}, respectively […].

[Lines 284 – 286: corrected new coefficient units] […] 0.33 [0.27−0.42] $·kg-sugars^{-1}, 0.34 [0.29−0.45] $·kg-sugars^{-1}, 0.32 [0.26−0.35] $·kg-sugars^{-1}, 0.32 [0.26−0.35] $·kg-sugars^{-1}, 0.30 [0.23−0.31] $·kg-sugars^{-1}, and 0.31 [0.24−0.32] $·kg-sugars^{-1} […].

[Lines 495 – 497: add citation to Supplementary Data 3] Although feedstock capacity uncertainties were included in all uncertainty analyses performed in this work (**Supplementary Data 3**), extending the proposed equation […].

Supplementary Information:

Supplementary Figure 6 caption. […] for biomanufacturing facilities producing potassium sorbate via TAL from sugarcane […].

Supplementary Figure 9 caption. Minimum product selling price (MPSP) […] at baseline productivity values (i.e., at 0.12 g-*fp*·L^{-1}·h^{-1} for TAL biomanufacturing facilities, 0.28 g-*fp*·L^{-1}·h^{-1} for 3HP biomanufacturing facilities, and 0.66 g-*fp*·L^{-1}·h^{-1} for succinic acid biomanufacturing facilities). […].

Supplementary Figure 10 caption. Minimum product selling price (MPSP) […] at baseline productivity values (i.e., at 0.12 g-*fp*·L^{-1}·h^{-1} for TAL biomanufacturing facilities, 0.28 g-*fp*·L^{-1}·h^{-1} for 3HP biomanufacturing facilities, and 0.66 g-*fp*·L^{-1}·h^{-1} for succinic acid biomanufacturing facilities). […].

Changes to address editorial requests:

See Author Checklist for details.
