# Supplementary material for: A unifying equation for fermentation sustainability across the titer-rate-yield landscape
Source: Nat Commun. 2026 Jul 10;17:8655. doi: 10.1038/s41467-026-75285-1 (PMC13490486; doi:10.1038/s41467-026-75285-1)
Supplement: Supplementary file 3 [file 41467_2026_75285_MOESM1_ESM.pdf]

Supplementary Information for

# A Unifying Equation for Fermentation Sustainability across the Titer-Rate-Yield Landscape

Sarang S. Bhagwat[1,2,*], Christopher V. Rao[1,3], Huimin Zhao[1,3], Vijay Singh[1,4], Jeremy S. Guest[1,2,5,*]

[1] DOE Center for Advanced Bioenergy and Bioproducts Innovation (CABBI), University of Illinois Urbana-Champaign, 1206 W. Gregory Drive, Urbana, IL 61801, USA

[2] The Grainger College of Engineering, Department of Civil and Environmental Engineering, University of Illinois Urbana-Champaign, 3221 Newmark Civil Engineering Laboratory, 205 N. Mathews Avenue, Urbana, IL 61801, USA

[3] Department of Chemical and Biomolecular Engineering, University of Illinois Urbana-Champaign, 215 Roger Adams Laboratory, 600 S. Mathews Avenue, Urbana, IL 61801, USA

[4] Department of Agricultural and Biological Engineering, University of Illinois Urbana-Champaign, 360 G Agricultural Engr Sciences Bld, 1304 W Pennsylvania Ave, Urbana, IL 61801, USA

[5] Institute for Sustainability, Energy, and Environment (iSEE), University of Illinois Urbana-Champaign, 1101 W. Peabody Drive, Urbana, IL 61801, USA

[*] Co-corresponding authors

Number of pages: 16

Number of figures: 11

**Table of Contents**

**Supplementary Section 1. Supplementary Notes including Process Description, Analysis Methods, and Results** .......... 3

***Supplementary Note 1.1. Fermentation Processes*** .......... 3

***Supplementary Note 1.2. Selection of Uncertainty Distributions*** .......... 3

***Supplementary Note 1.3. Influence of Coefficient Values on the Shape of Hyperbolae Described by Equation 5*** .......... 4

**Supplementary Section 2. Supplementary Figures** .......... 5

**Supplementary References** .......... 16

## Supplementary Section 1. Supplementary Notes including Process Description, Analysis Methods, and Results

### *Supplementary Note 1.1. Fermentation Processes*

The fermentation mode was assumed to be fed-batch for all biomanufacturing facilities. The retention time for the fermentation reactor was determined through the designated titer and productivity.

The sugar solution stream from the pretreatment and saccharification of the feedstock needs further processing so that the sucrose, glucose, and xylose are present at the concentrations required to achieve the targeted fermentation product (*fp*) concentration. To facilitate this, the sugar solution stream is passed through a multiple-effect evaporator which initially concentrates the stream to a total sugar concentration of 29.8 wt% (for corn stover slurry streams), 52.8 wt% (for sugarcane juice streams), or 53.3 wt% (for corn slurry streams). Feed dextrose monohydrate has a glucose content of 90.9 wt% and is not evaporated. The stream is then diluted such that the final concentration of *fp* in the fermentation reactor is the target concentration (hereafter referred to as the titer). Note that biomanufacturing facilities can be configured to evaporate sugar solutions directly to the concentration required to achieve the targeted titer of the product (based on the fermentation yield).[1–4] However, optimized feeding strategies can often involve feeding more highly concentrated sugar solutions into the fermentation reactor,[5] especially with high initial working volume fractions. Therefore, we conservatively assumed sugar solutions were evaporated to concentrations higher than required to achieve any of the targeted titers for the 32 biorefineries (biomanufacturing facilities) evaluated in this work.

As the multiple effect evaporator is set to concentrate sugar solutions up to fixed concentrations of sugars, there is an implicit maximum titer that can be achieved at any given yield on sugars during fermentation. This manifests as an infeasible region in the upper-left portion of the fermentation titer-yield space (represented by white regions in **Figure 1** and **Supplementary Figures 1, 2**, **3B**, **5**, **6A**, **8**, **9**, and **10**).

Lists of all units comprising TAL,[1,2] 3HP,[3] and succinic acid biomanufacturing facilities,[4] including fermentation process units, are detailed in the original studies developing fermentation process models for those biomanufacturing facilities.

### *Supplementary Note 1.2. Selection of Uncertainty Distributions*

Uncertain parameters and their baseline values and distributions were based on the original studies that performed uncertainty analyses for biomanufacturing facilities producing TAL,[1] potassium sorbate,[2] acrylic acid via 3HP,[3] and succinic acid[4] for all parameters, except as follows:

1. Fermentation yield, titer, and productivity were set based on the yield-titer-productivity combination being evaluated, without uncertainty attributed to them. (Uncertainty analyses were performed at each of 25 yield-titer combinations at baseline productivity values for each of 24 biomanufacturing facilities; **Figure 1** and **Supplementary Figures 1 and 2**)
2. Baseline feedstock capacities were set to 1220 metric ton·$d^{-1}$ for dextrose monohydrate, 1370 metric ton·$d^{-1}$ for corn, 11500 metric ton·$d^{-1}$ for sugarcane, and 2060 metric ton·$d^{-1}$ for corn stover. Triangular distributions with the lower bound, most common value, and upper bound represented by 80%, 100%, and 120% of baseline values, respectively, were used in the uncertainty analyses rather than production capacities.

For all uncertainty analyses, samples were generated for each of the parameters listed in **Supplementary Data 3** using Latin hypercube sampling.

***Supplementary Note 1.3. Influence of Coefficient Values on the Shape of Hyperbolae Described by Equation 5***

In **Equation 5**, when $a$ changes, the shape and position (along both yield and titer axes) of a contour associated with a particular MPSP changes (**Figure 3**). However, although increasing $a$ results in each yield-titer combination being associated with an increased MPSP, it does not affect the overall shape of a contour plot depicting MPSP at alternative yield-titer combinations. Increasing $b$ shifts all contours farther along the yield axis (**Figure 3B**). While the shape of the associated contour changes to have less sharp features (i.e., lower linear eccentricity), the contour is consistently sharper than the contour that resided in the same position (i.e., with the same asymptotes) prior to increasing $b$, resulting in a contour plot that shows greater benefits to MPSP from improvements to yield than prior to increasing $b$. Similarly, increasing $c$ shifts all contours along the titer axis (**Figure 3C**) and—despite reducing the sharpness of each contour—results in a contour plot that shows greater benefits to MPSP from improvements to titer than prior to increasing $c$. Increasing $d$ does not shift the position of contours associated with any MPSP, but reduces the sharpness of each contour (**Figure 3D**), resulting in a contour plot that reveals more evenly balanced benefits to MPSP from improvements to either yield or titer than prior to increasing $d$. Finally, the effects of each of the coefficients $a$, $b$, $c$, and $d$ were greater for contours associated with lower MPSPs, as this resulted in smaller denominators for the asymptotes (**Supplementary Equations 1.3.1 and 1.3.2** represent asymptotes parallel to the titer and yield axes, respectively) and the linear eccentricity (**Supplementary Equation 1.3.3**).

$$\text{yield} - \frac{b}{\text{MPSP} - a} = 0 \qquad \text{(Supplementary Equation 1.3.1)}$$

$$\text{titer} - \frac{c}{\text{MPSP} - a} = 0 \qquad \text{(Supplementary Equation 1.3.2)}$$

$$\text{linear eccentricity} = 2 * \sqrt{\left(\frac{d + \frac{b*c}{\text{MPSP} - a}}{\text{MPSP} - a}\right)} \qquad \text{(Supplementary Equation 1.3.3)}$$

## Supplementary Section 2. Supplementary Figures

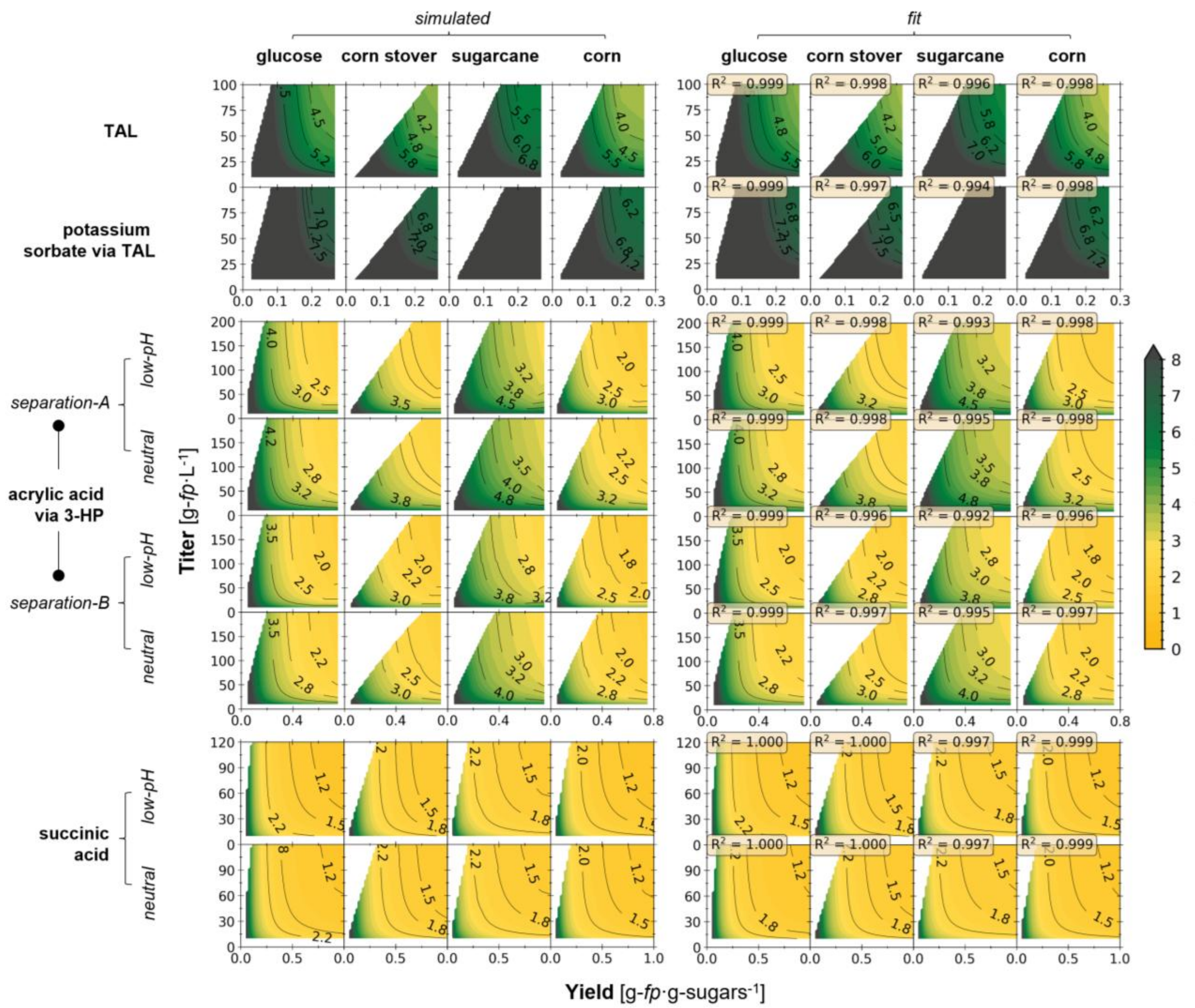


**Supplementary Figure 1.** Minimum product selling price (MPSP) of the main biomanufacturing facility product (*p*) across fermentation yields (*x*-axes) and titers (*y*-axes) at one-fifth the baseline productivity values (i.e., at 0.024 g-*fp*·$L^{-1}$·$h^{-1}$ for TAL biomanufacturing facilities, 0.066 g-*fp*·$L^{-1}$·$h^{-1}$ for 3HP biomanufacturing facilities, and 0.132 g-*fp*·$L^{-1}$·$h^{-1}$ for succinic acid biomanufacturing facilities). For a given point on the figure, the *x*-axis value represents the overall fermentation yield (g-*fp*·g-$sugars^{-1}$), the *y-axis* value represents the titer (g-*fp*·$L^{-1}$), and the color represents MPSP. The white region to the upper left of each plot represents infeasible yield-titer combinations (described in **Supplementary Note 1.1**). Panels in the first four columns show results from simulation and evaluation of biomanufacturing facilities at 2500 fermentation yield-titer combinations each, while panels in the last four columns show results from fitting the empirical coefficients $a$, $b$, $c$, and $d$ in **Equation IV** to the results from simulation and evaluation (maximizing $R^2$, shown to the upper left of each figure; values of empirical coefficients are provided in **Supplementary Data 2**). Rows represent biomanufacturing facilities producing TAL (*TAL*), potassium sorbate via TAL (*potassium sorbate via TAL*), acrylic acid via 3HP (*acrylic acid via 3HP*) under low-pH (*low-pH*) and neutral fermentation regimes (*neutral*) with separation process A (*separation-A*)[6] or B (*separation-B*)[7], and succinic acid (*succinic acid*) under low-pH (*low-pH*) and neutral fermentation regimes (*neutral*). Columns represent biomanufacturing facilities accepting dextrose monohydrate (*glucose*), sugarcane (*sugarcane*), corn stover (*corn stover*), and corn (*corn*) as the feedstock. All biomanufacturing facility configurations are described in **Figure 1**. Source data for this figure are available in the **Source Data** file.

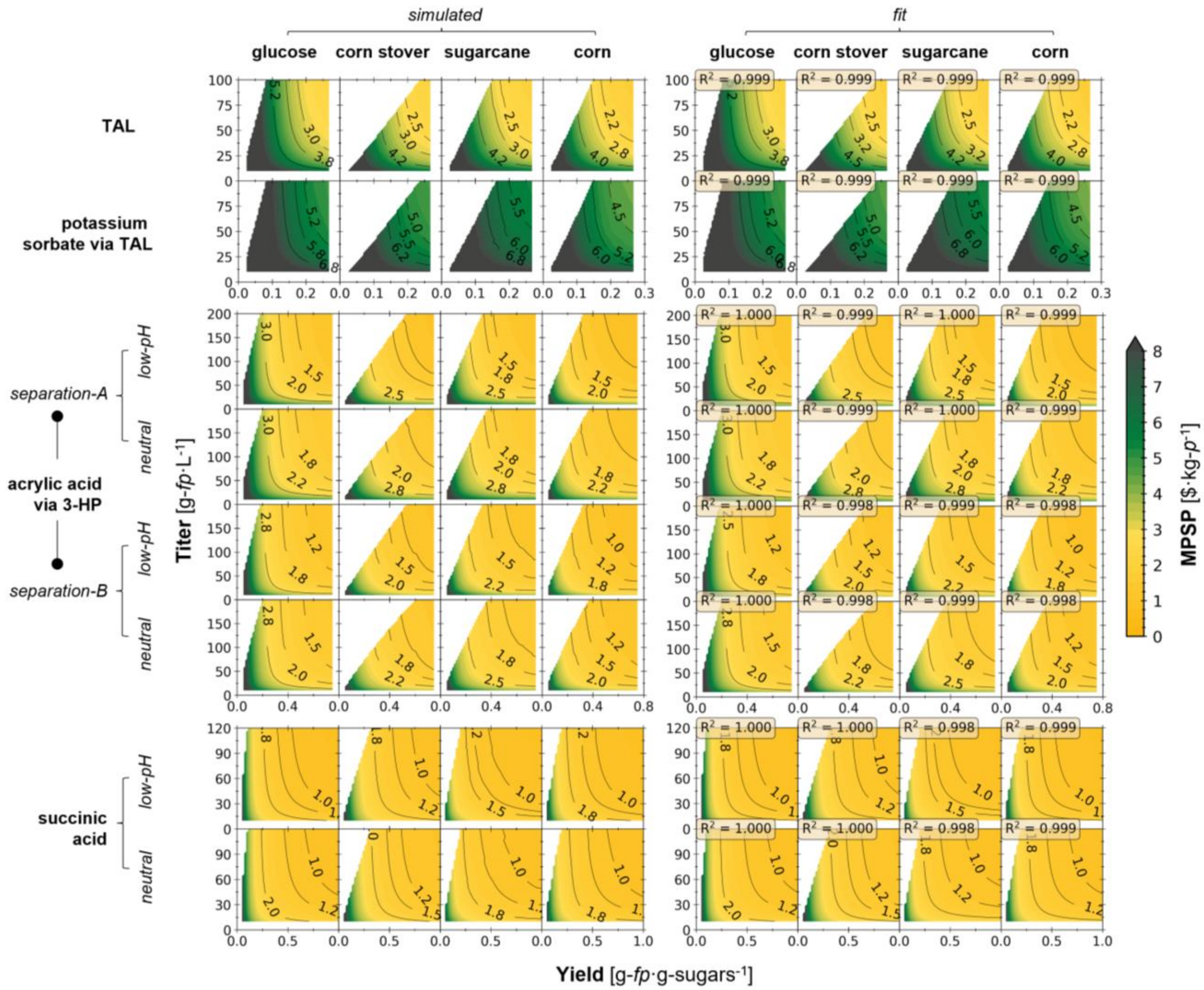


**Supplementary Figure 2.** Minimum product selling price (MPSP) of the main biomanufacturing facility product (*p*) across fermentation yields (*x*-axes) and titers (*y*-axes) at five times the baseline productivity values (i.e., at 0.600 g-*fp*·$L^{-1}$·$h^{-1}$ for TAL biomanufacturing facilities, 1.650 g-*fp*·$L^{-1}$·$h^{-1}$ for 3HP biomanufacturing facilities, and 3.300 g-*fp*·$L^{-1}$·$h^{-1}$ for succinic acid biomanufacturing facilities). For a given point on the figure, the *x*-axis value represents the overall fermentation yield (g-*fp*·g-$sugars^{-1}$), the *y-axis* value represents the titer (g-*fp*·$L^{-1}$), and the color represents MPSP. The white region to the upper left of each plot represents infeasible yield-titer combinations (described in **Supplementary Note 1.1**). Panels in the first four columns show results from simulation and evaluation of biomanufacturing facilities at 2500 fermentation yield-titer combinations each, while panels in the last four columns show results from fitting the empirical coefficients $a$, $b$, $c$, and $d$ in **Equation IV** to the results from simulation and evaluation (maximizing $R^2$, shown to the upper left of each figure; values of empirical coefficients are provided in **Supplementary Data 2**). Rows represent biomanufacturing facilities producing TAL (*TAL*), potassium sorbate via TAL (*potassium sorbate via TAL*), acrylic acid via 3HP (*acrylic acid via 3HP*) under low-pH (*low-pH*) and neutral fermentation regimes (*neutral*) with separation process A (*separation-A*)[6] or B (*separation-B*)[7], and succinic acid (*succinic acid*) under low-pH (*low-pH*) and neutral fermentation regimes (*neutral*). Columns represent biomanufacturing facilities accepting dextrose monohydrate (*glucose*), sugarcane (*sugarcane*), corn stover (*corn stover*), and corn (*corn*) as the feedstock. All biomanufacturing facility configurations are described in **Figure 1**. Source data for this figure are available in the **Source Data** file.

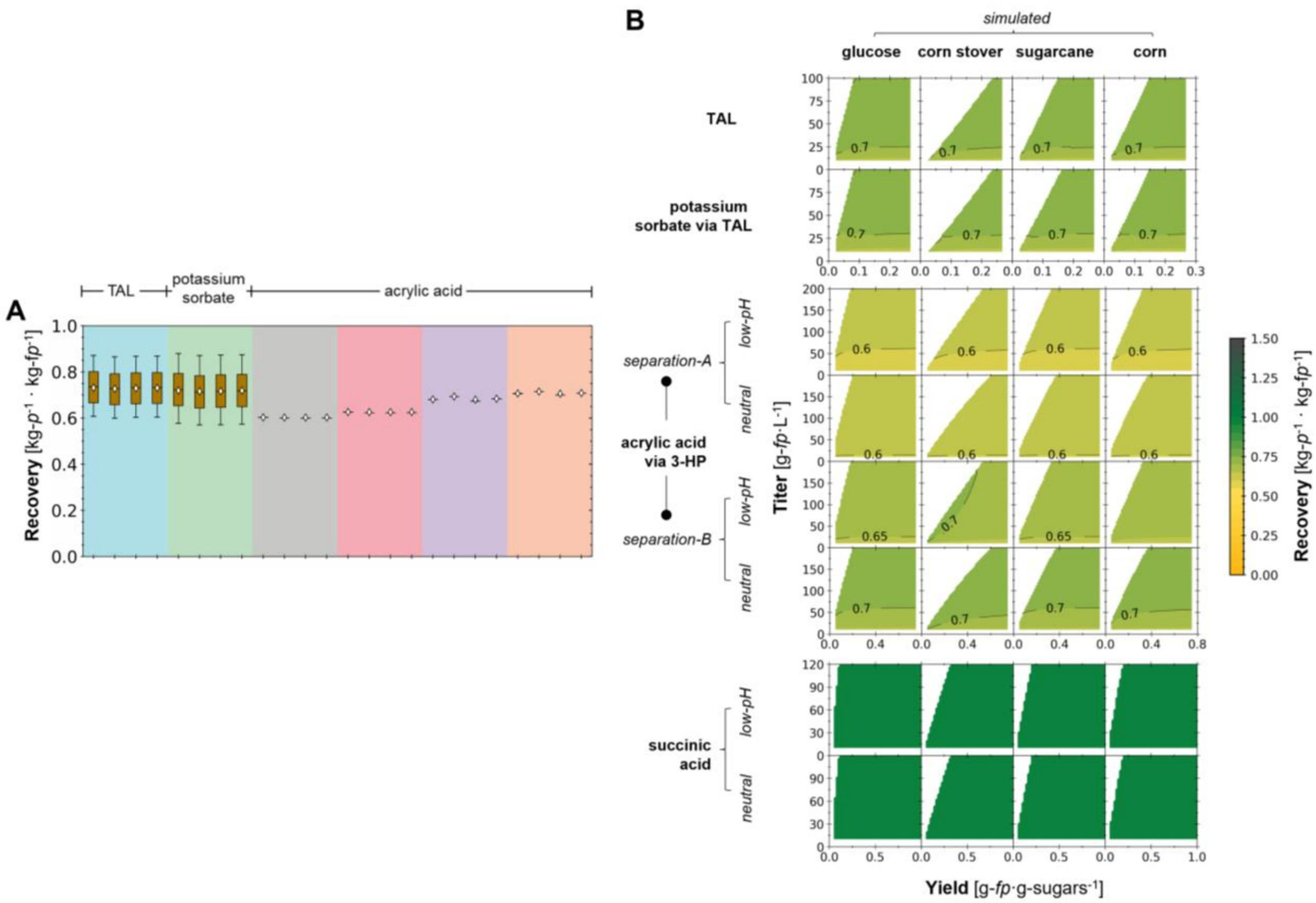


**Supplementary Figure 3.** (A) Uncertainties (box-and-whisker plots) in post-fermentation recoveries, and (B) post-fermentation recoveries across yield-titer combinations at baseline productivity values (0.12 g-*fp*·L$^{-1}$·h$^{-1}$ for TAL biomanufacturing facilities, 0.28 g-*fp*·L$^{-1}$·h$^{-1}$ for 3HP biomanufacturing facilities, and 0.66 g-*fp*·L$^{-1}$·h$^{-1}$ for succinic acid biomanufacturing facilities) for 32 biomanufacturing facilities. In the box-and-whisker plots, whiskers, boxes, and middle lines represent 5th/95th, 25th/75th, and 50th percentiles, respectively, from 1000 Monte Carlo simulations at each of 25 fermentation yield-titer combinations at baseline productivity values for each of 24 biomanufacturing facilities (i.e., 600000 Monte Carlo simulations in total; *n* = 25000 for each of the 24 plots shown). Plot background colors represent biomanufacturing facilities producing TAL as the main product (blue), biomanufacturing facilities producing potassium sorbate via TAL (green), biomanufacturing facilities producing acrylic acid via 3HP under low-pH (gray) and neutral fermentation regimes (pink) with separation process A, and biomanufacturing facilities producing acrylic acid via 3HP under low-pH (purple) and neutral fermentation regimes (orange) with separation process B. Within each background color, serially from left to right, box-and-whisker plots are shown for biomanufacturing facilities accepting dextrose monohydrate, corn stover, sugarcane, and corn as the feedstock. The main biomanufacturing facility product (*p*) is indicated above. Diamonds represent baseline values (used in **Figure 2**; a full list of baseline coefficient values is provided in **Supplementary Data 2**). Source data for this figure are available in the **Source Data** file.

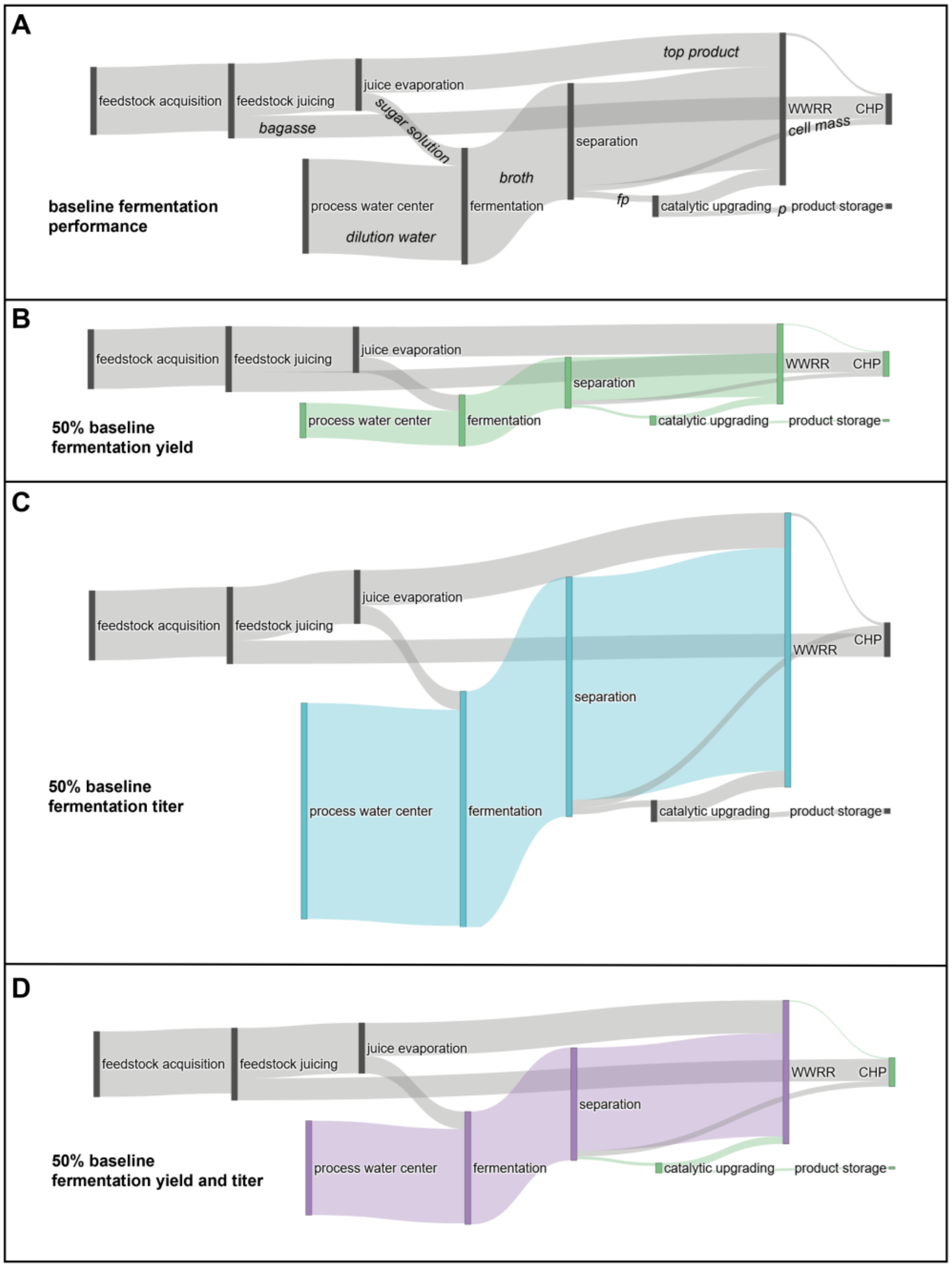


**Supplementary Figure 4.** Sankey diagrams depicting process-level mass flows (stream names in italicized text) for simulations of the biomanufacturing facility producing acrylic acid via 3HP at low pH from sugarcane using separation process B. Vertical bars indicate process blocks corresponding to adjacent names (non-italicized text). Abbreviations denote wastewater resource recovery (WWRR) and combined heat and power generation (CHP). Simulations with (A) baseline fermentation performance (fermentation yield of 0.73 g-*fp*·g-sugars$^{-1}$, titer of 62.5 g-*fp*·L$^{-1}$, and productivity of 0.28 g-*fp*·L$^{-1}$·h$^{-1}$), (B) fermentation yield changed to 50% of the baseline value, (C) fermentation titer changed to 50% of the baseline value, and (D) both fermentation yield and titer changed to 50% of their respective baseline values are shown. For (B) and (C), green and blue colors indicate altered flows and unit operation designs relative to the baseline simulation. For (D), purple streams indicate flows and unit operation designs that were altered relative to the baseline for (B) and (C), but not for (D), as those flows changed linearly with the ratio of fermentation yield to titer. For (D), green streams indicate flows and unit operation designs that were altered for both (B) and (D) relative to the baseline simulation, as those flows changed linearly with fermentation yield but did not change with titer. Source data for this figure are available in the **Source Data** file.

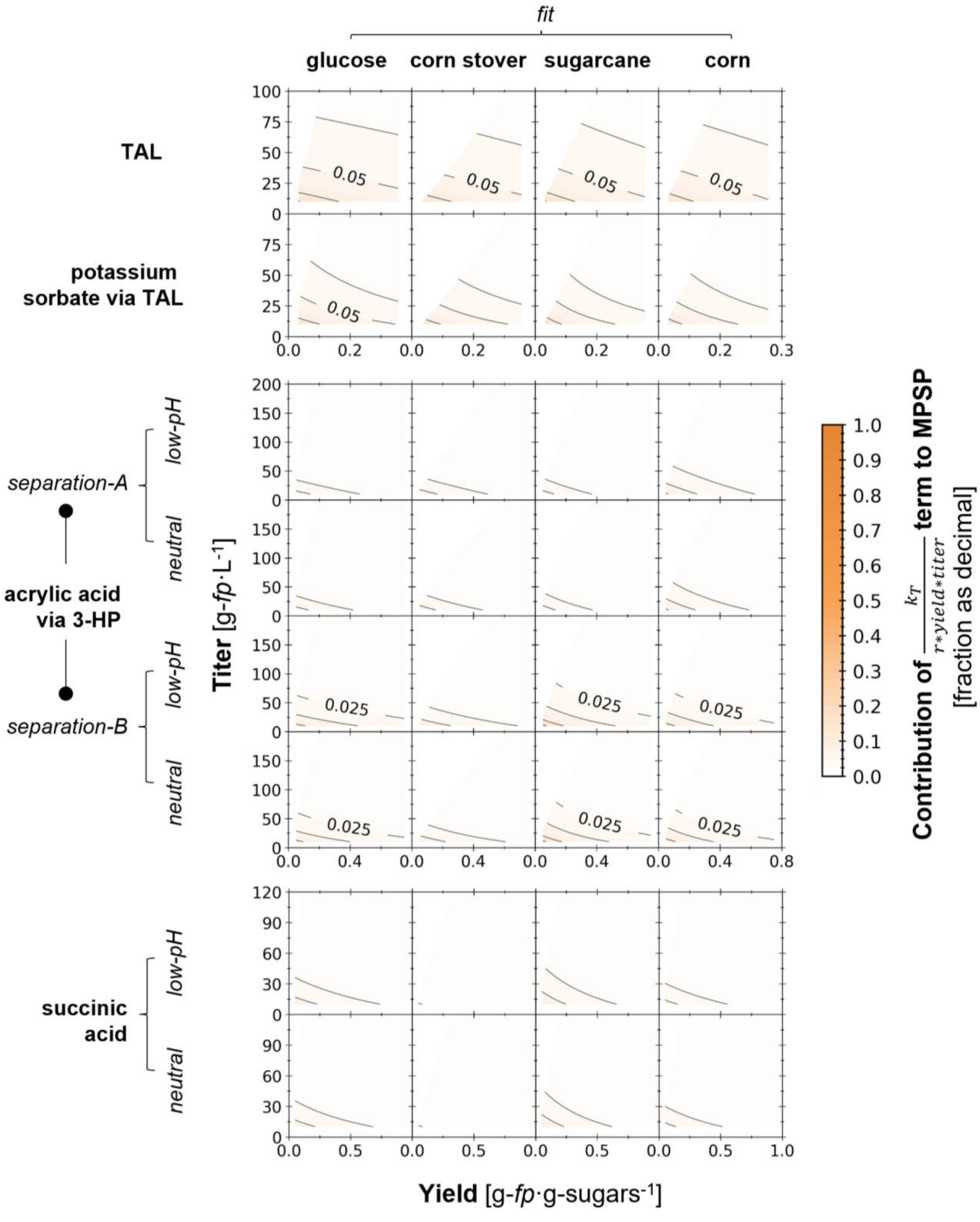


**Supplementary Figure 5.** Contribution of the term $\frac{k_T}{r*yield*titer}$ to MPSP (estimated using **Equation XI**) vs fermentation titer and yield at baseline productivity values (0.12 g-*fp*·$L^{-1}$·$h^{-1}$ for TAL biomanufacturing facilities, 0.28 g-*fp*·$L^{-1}$·$h^{-1}$ for 3HP biomanufacturing facilities, and 0.66 g-*fp*·$L^{-1}$·$h^{-1}$ for succinic acid biomanufacturing facilities) for 32 biomanufacturing facilities. Source data for this figure are available in the **Source Data** file.

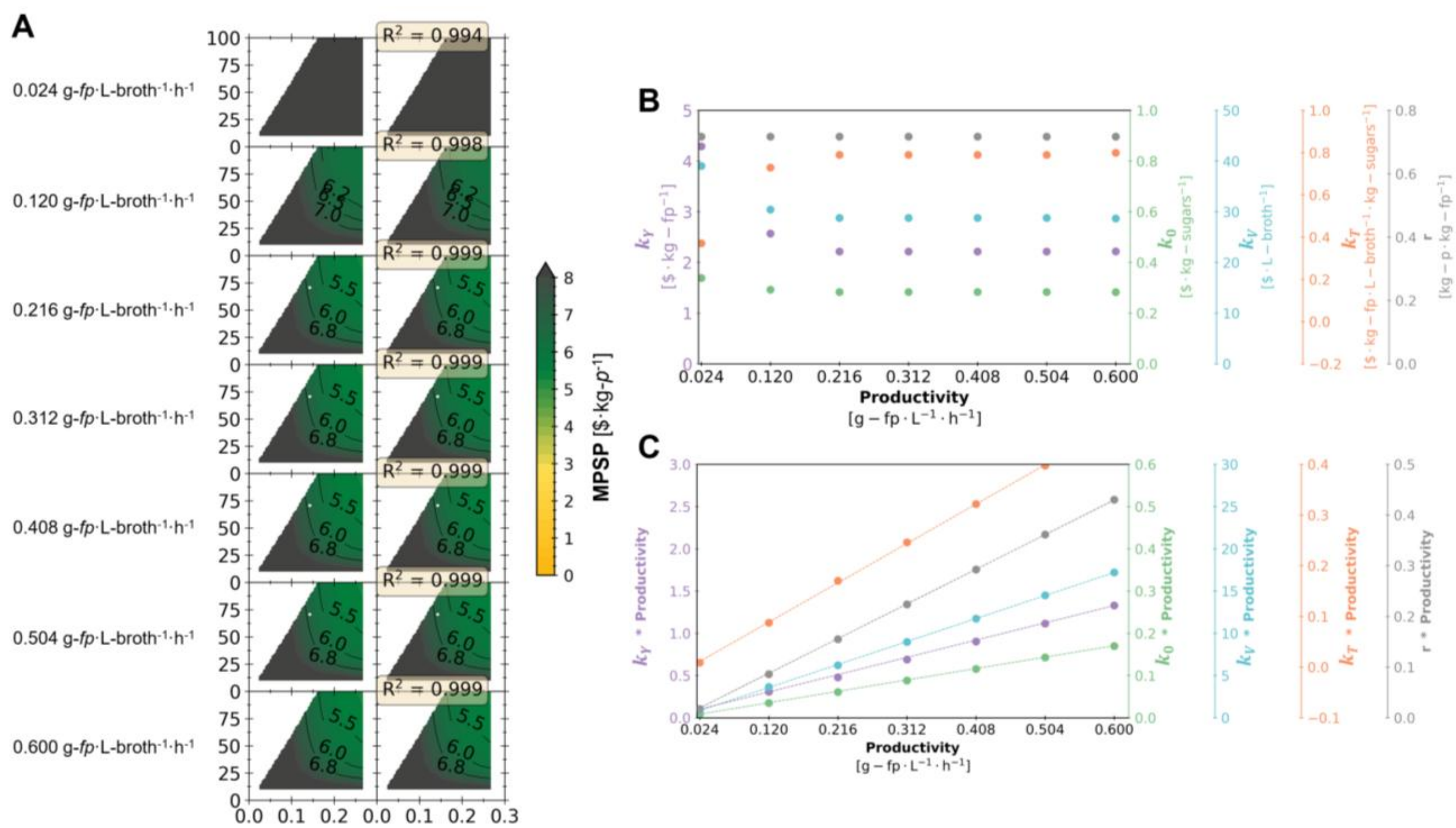


**Supplementary Figure 6.** (A) Simulated values for MPSP across 2500 combinations of values for fermentation titer and yield at seven alternative productivities for biomanufacturing facilities producing potassium sorbate via TAL from sugarcane (*left column panels*), and results from fitting **Equation XII** to simulation results (*right column panels*). (B) Values for coefficients of **Equation XII** when fit to simulation results for the baseline at alternative values for fermentation productivity. (C) Mathematical product of each coefficient value and fermentation productivity plotted at alternative values of fermentation productivity. Straight lines (dashed) passing through point values indicate the linear relationships described in **Equations XIII–XVI**. Source data for this figure are available in the **Source Data** file.

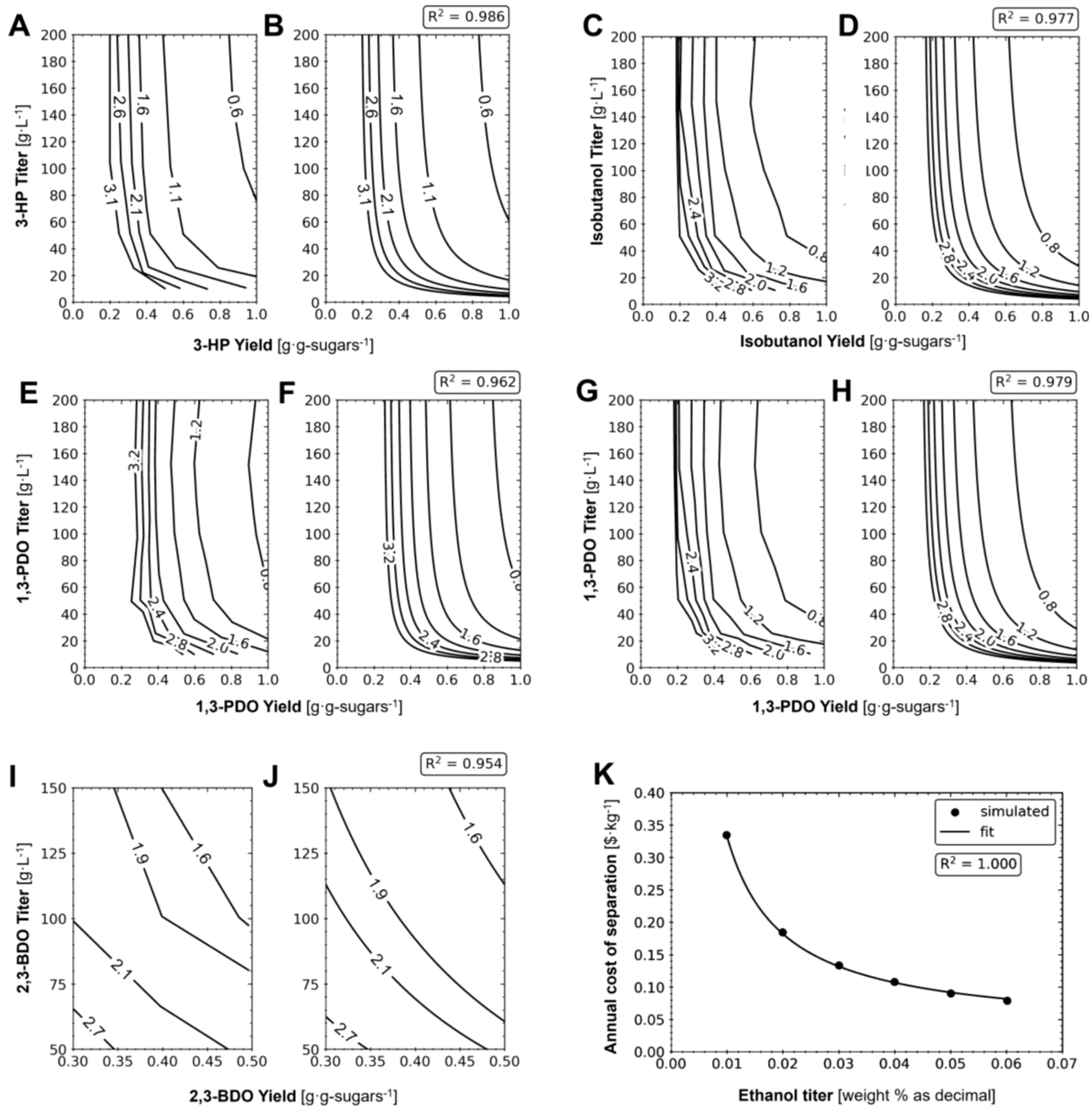


**Supplementary Figure 7.** Minimum product selling price (inline contour labels) as a function of fermentation yield and titer of: (A) 3-hydroxypropionic acid (3-HP) from Gunukula *et al.*, who used SuperPro Designer;[8] (C) isobutanol from Gunukula *et al.*, who used SuperPro Designer;[8] (E) 1,3-propanediol (1,3-PDO) produced under aerobic conditions from Gunukula *et al.*, who used SuperPro Designer;[8] (G) 1,3-propanediol (1,3-PDO) produced under microaerobic conditions from Gunukula *et al.*, who used SuperPro Designer;[8] (I) 2,3-butanediol from Sikazwe *et al.*, who used Aspen Plus.[9] Results of fitting the empirical coefficients *a*, *b*, *c*, and *d* in **Equation IV** to the data from (B, D, F, H) Gunukula *et al.* and (J) Sikazwe *et al.* are shown, with values for coefficient of determination ($R^2$) included near the top-right corner of each panel. (K) Annual cost of separation (*y*-axis) of ethanol at alternative values of crude ethanol concentration (*x*-axis) from Janković *et al.*[10], who used Aspen Plus (*simulated*), and the results of fitting the empirical coefficients *a* and *c* in **Equation IV** (*fit*; *b* and *d* were set to zero) to the data from Janković *et al.*, with an $R^2$ of 1.000. Source data for this figure are available in the **Source Data** file.

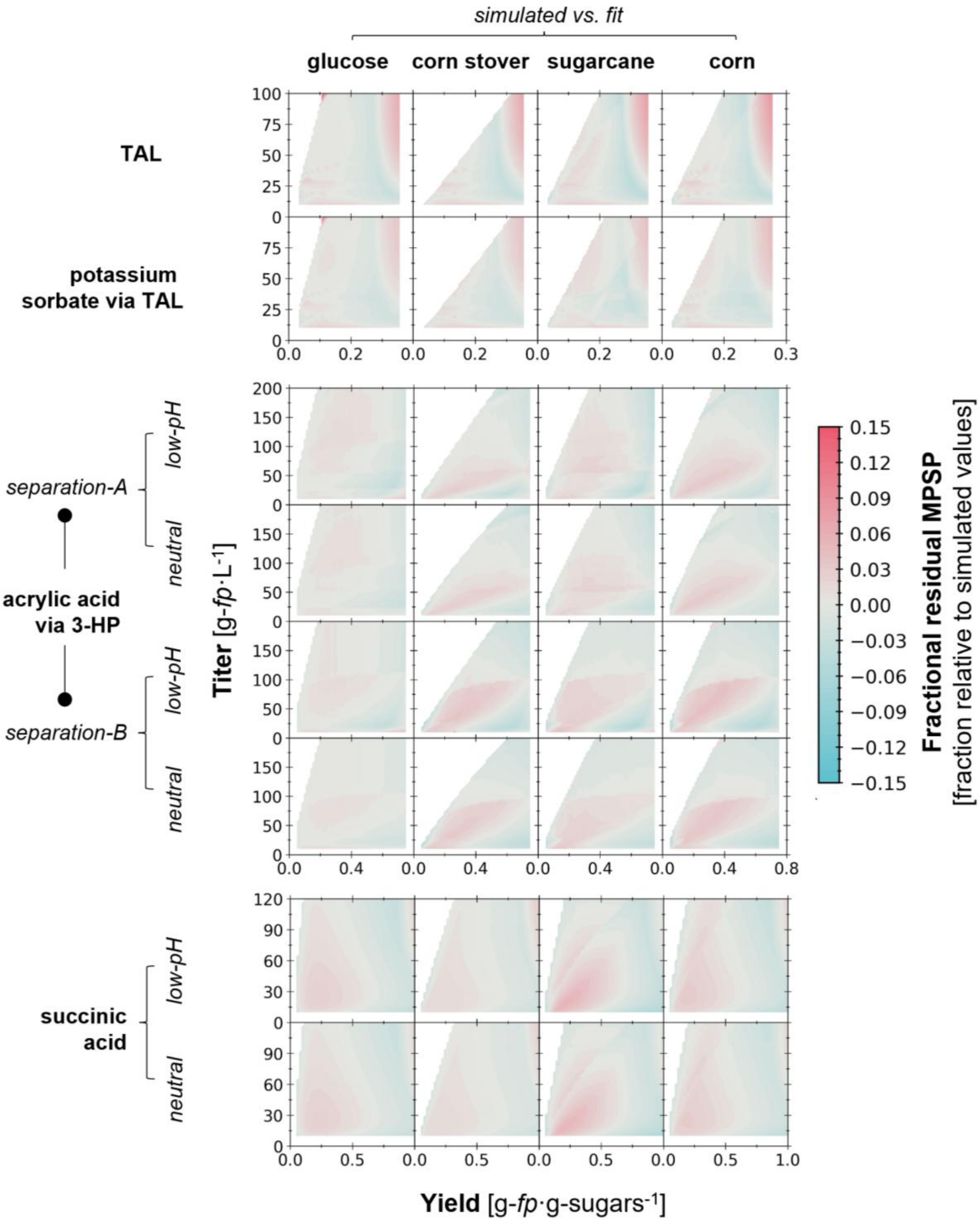


**Supplementary Figure 8.** MPSP residuals represented as fractional residual values calculated as the difference between the *simulated* and *fit* MPSP values divided by the *simulated* MPSP value (*simulated* and *fit* MPSP values shown in **Figure 2**) across fermentation titer and yield at baseline productivity values (0.12 g-*fp*·$L^{-1}$·$h^{-1}$ for TAL biomanufacturing facilities, 0.28 g-*fp*·$L^{-1}$·$h^{-1}$ for 3HP biomanufacturing facilities, and 0.66 g-*fp*·$L^{-1}$·$h^{-1}$ for succinic acid biomanufacturing facilities) at 2500 fermentation yield-titer combinations each for 32 biomanufacturing facilities. Source data for this figure are available in the **Source Data** file.

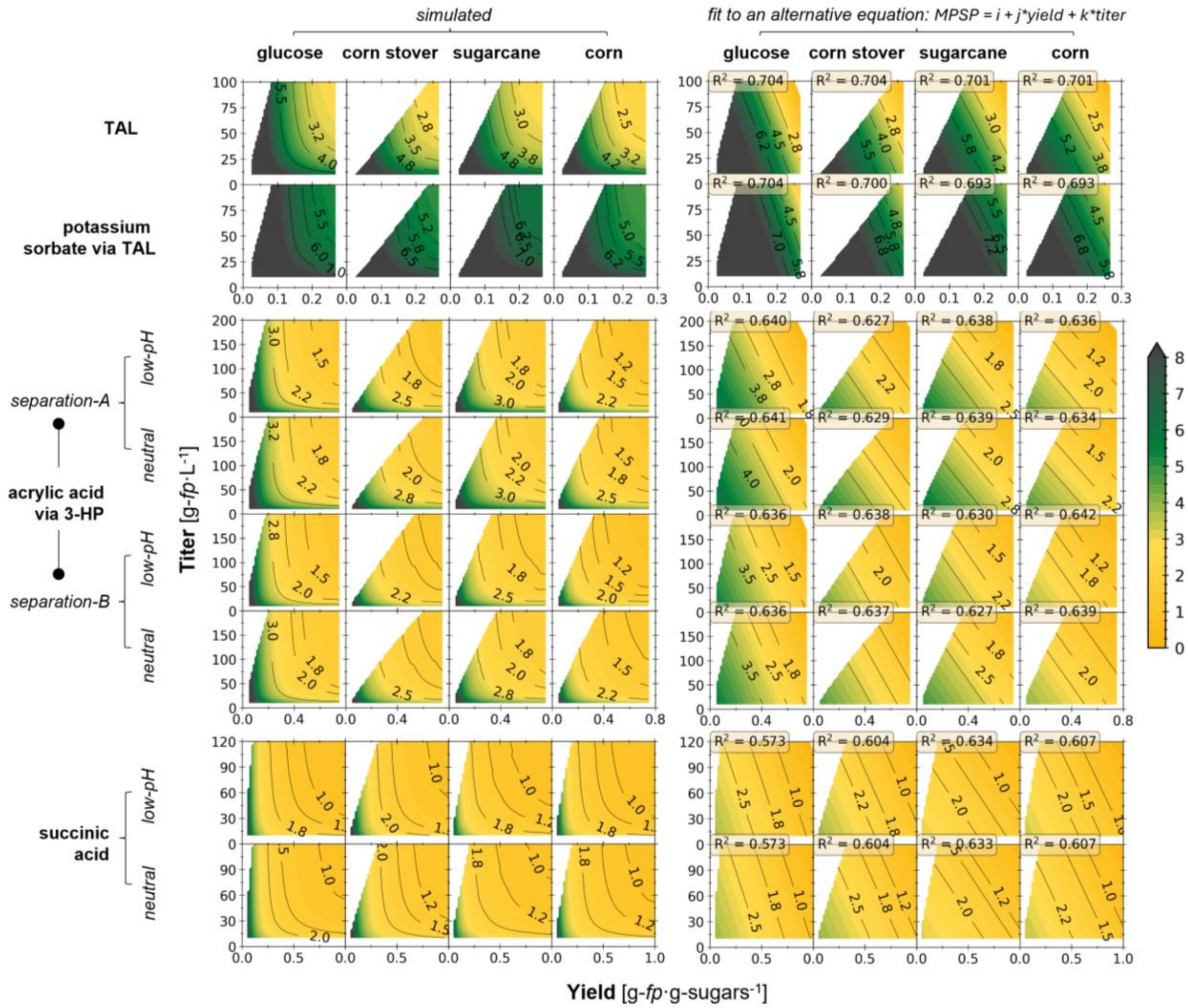


**Supplementary Figure 9.** Minimum product selling price (MPSP) of the main biomanufacturing facility product (*p*) across fermentation yields (*x*-axes) and titers (*y*-axes) at baseline productivity values (i.e., at 0.12 g-*fp*·$L^{-1}$·$h^{-1}$ for TAL biomanufacturing facilities, 0.28 g-*fp*·$L^{-1}$·$h^{-1}$ for 3HP biomanufacturing facilities, and 0.66 g-*fp*·$L^{-1}$·$h^{-1}$ for succinic acid biomanufacturing facilities). For a given point on the figure, the *x*-axis value represents the overall fermentation yield (g-*fp*·g-$sugars^{-1}$), the *y-axis* value represents the titer (g-*fp*·$L^{-1}$), and the color represents MPSP. The white region to the upper left of each plot represents infeasible yield-titer combinations (described in **Supplementary Note 1.1**). Panels in the first four columns show results from simulation and evaluation of biomanufacturing facilities at 2500 fermentation yield-titer combinations each, while panels in the last four columns show results from fitting to an alternative equation (multivariate linear regression, i.e., fitting *MPSP = i + j*yield + k*titer*, where *i*, *j*, and *k* are empirical coefficients). Rows represent biomanufacturing facilities producing TAL (*TAL*), potassium sorbate via TAL (*potassium sorbate via TAL*), acrylic acid via 3HP (*acrylic acid via 3HP*) under low-pH (*low-pH*) and neutral fermentation regimes (*neutral*) with separation process A (*separation-A*) or B (*separation-B*), and succinic acid (*succinic acid*) under low-pH (*low-pH*) and neutral fermentation regimes (*neutral*). Columns represent biomanufacturing facilities accepting dextrose monohydrate (*glucose*), sugarcane (*sugarcane*), corn stover (*corn stover*), and corn (*corn*) as the feedstock (all biomanufacturing facility configurations are described in **Figure 1**). Source data for this figure are available in the **Source Data** file.

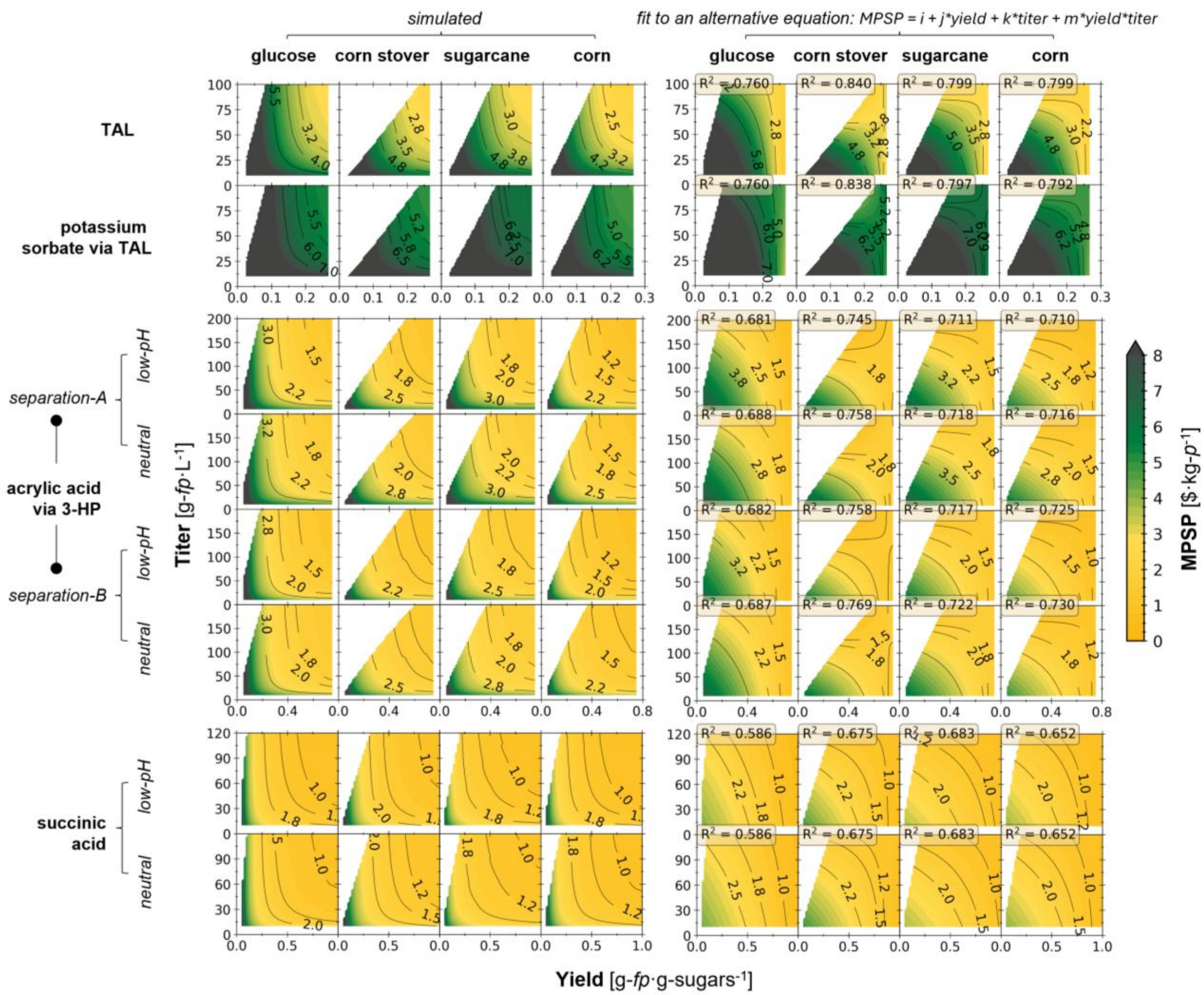


**Supplementary Figure 10.** Minimum product selling price (MPSP) of the main biomanufacturing facility product (*p*) across fermentation yields (*x*-axes) and titers (*y*-axes) at baseline productivity values (i.e., at 0.12 g-*fp*·$L^{-1}$·$h^{-1}$ for TAL biomanufacturing facilities, 0.28 g-*fp*·$L^{-1}$·$h^{-1}$ for 3HP biomanufacturing facilities, and 0.66 g-*fp*·$L^{-1}$·$h^{-1}$ for succinic acid biomanufacturing facilities). For a given point on the figure, the *x*-axis value represents the overall fermentation yield (g-*fp*·g-$sugars^{-1}$), the *y-axis* value represents the titer (g-*fp*·$L^{-1}$), and the color represents MPSP. The white region to the upper left of each plot represents infeasible yield-titer combinations (described in **Supplementary Note 1.1**). Panels in the first four columns show results from simulation and evaluation of biomanufacturing facilities at 2500 fermentation yield-titer combinations each, while panels in the last four columns show results from fitting to an alternative equation (*MPSP* = *i* + *j*yield* + *k*titer* + *m*yield*titer*, where *i*, *j*, *k*, and *m* are empirical coefficients). Rows represent biomanufacturing facilities producing TAL (*TAL*), potassium sorbate via TAL (*potassium sorbate via TAL*), acrylic acid via 3HP (*acrylic acid via 3HP*) under low-pH (*low-pH*) and neutral fermentation regimes (*neutral*) with separation process A (*separation-A*) or B (*separation-B*), and succinic acid (*succinic acid*) under low-pH (*low-pH*) and neutral fermentation regimes (*neutral*). Columns represent biomanufacturing facilities accepting dextrose monohydrate (*glucose*), sugarcane (*sugarcane*), corn stover (*corn stover*), and corn (*corn*) as the feedstock (all biomanufacturing facility configurations are described in **Figure 1**). Source data for this figure are available in the **Source Data** file.

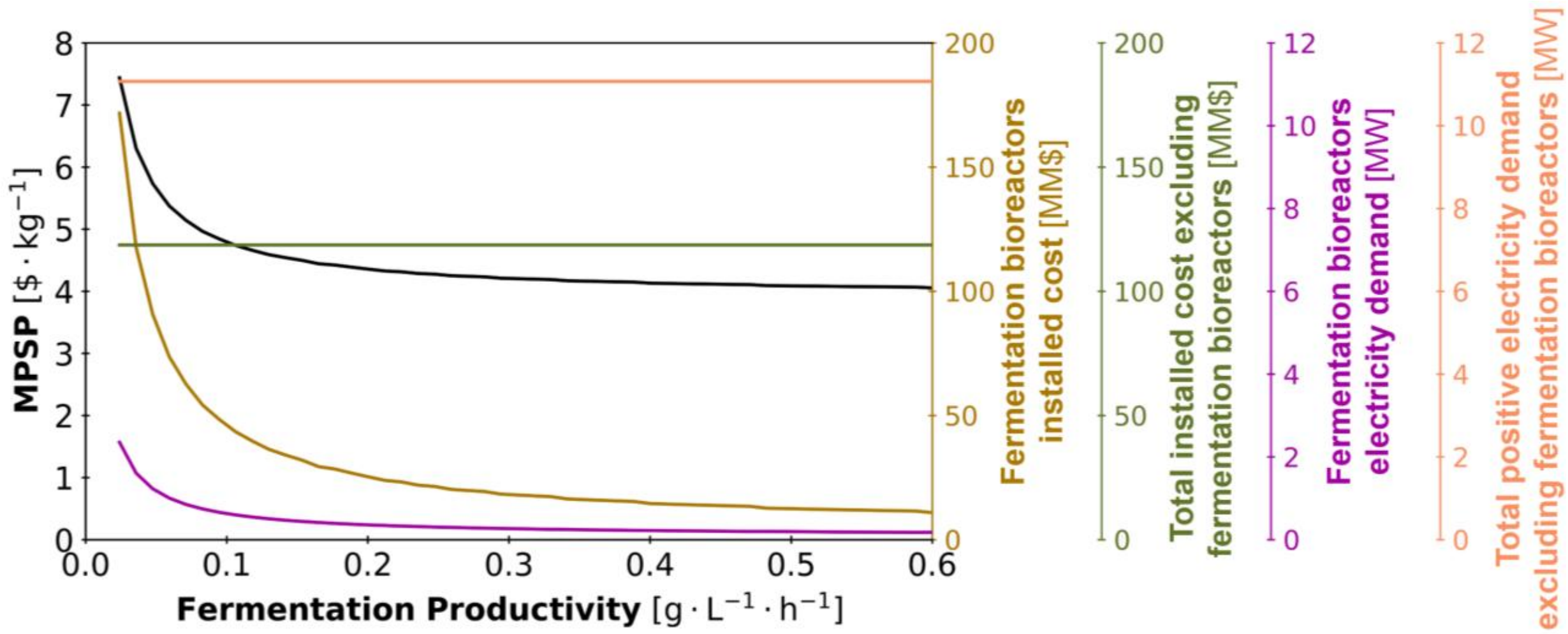


**Supplementary Figure 11.** For biomanufacturing facilities producing TAL from sugarcane, the minimum product selling price ($\$\cdot kg^{-1}$), installed equipment cost of bioreactors for fermentation and the seed train (MM$), the total installed equipment cost of the facility excluding bioreactors for fermentation and the seed train (MM$), the electricity demand of bioreactors for fermentation and the seed train (MW), and the total positive electricity demand of the facility excluding bioreactors for fermentation and the seed train (MW) simulated at 50 alternative values of fermentation productivity ranging from 0.024–6.000 $g\cdot L^{-1}\cdot h^{-1}$ are shown. Axis colors correspond to the colors of the curves. Source data for this figure are available in the **Source Data** file.

**Supplementary References**


1. Bhagwat, S. S. *et al.* Sustainable Triacetic Acid Lactone Production from Sugarcane and Sweet Sorghum by Fermentation and Fractional Crystallization. *ACS Sustainable Chem. Eng.* **13**, 17794–17805 (2025).
2. Kim, M. S. *et al.* Sustainable potassium sorbate production from triacetic acid lactone in food-grade solvents. *Green Chem.* **27**, 6087–6104 (2025).
3. Tan, S. I.; Bhagwat, S. S.; Martin, T. A. *et al.* High yield production of 3-hydroxypropionic acid using Issatchenkia orientalis. *Nat. Commun.* **17**, 899 (2026).
4. Tran, V. G. *et al.* An end-to-end pipeline for succinic acid production at an industrially relevant scale using Issatchenkia orientalis. *Nat. Commun.* **14**, 6152 (2023).
5. Mears, L., Stocks, S. M., Sin, G. & Gernaey, K. V. A review of control strategies for manipulating the feed rate in fed-batch fermentation processes. *Journal of Biotechnology* **245**, 34–46 (2017).
6. Mahata, C., Mishra, S. M. & Singh, V. 3-Hydroxypropionic acid recovery from fermentation broth through novel downstream processing: technoeconomic analysis. *Bioresour. Technol.* **445**, 134094 (2026).
7. Bhagwat, S. S. *et al.* Sustainable Production of Acrylic Acid via 3-Hydroxypropionic Acid from Lignocellulosic Biomass. *ACS Sustainable Chem. Eng.* **9**, 16659–16669 (2021).
8. Gunukula, S., Runge, T. & Anex, R. Assessment of Biocatalytic Production Parameters to Determine Economic and Environmental Viability. *ACS Sustainable Chem. Eng.* **5**, 8119–8126 (2017).
9. Sikazwe, M. K., Louw, J. & Görgens, J. F. Microbe and bioprocess performances for sustainable production of biobased 2,3-butanediol in a sugarcane biorefinery; a technoeconomic and environmental analysis. *Clean Techn Environ Policy* **26**, 4505–4519 (2024).
10. Janković, T., Straathof, A. J. J. & Kiss, A. A. Advanced downstream processing of bioethanol from syngas fermentation. *Separation and Purification Technology* **322**, 124320 (2023).
