# Supplementary material for: A unifying equation for fermentation sustainability across the titer-rate-yield landscape
Source: Nat Commun. 2026 Jul 10;17:8655. doi: 10.1038/s41467-026-75285-1 (PMC13490486; doi:10.1038/s41467-026-75285-1)
Supplement: Supplementary file 4 [file 41467_2026_75285_MOESM2_ESM.pdf]

Description of Additional Supplementary Files

Supplementary Data 1. List of the 32 biomanufacturing facilities evaluated in this study, shown in the same order as for panels in **Figure 2** in the manuscript (when members of each row are read left-to-right for all rows read top-to-bottom). An overview of process alternatives is shown in **Figure 1**.

Supplementary Data 2. Values of empirical coefficients of **Equation XI** after maximizing the coefficient of determination, R^2, to fit to the results from simulating all 32 biomanufacturing facilities across 2500 combinations of fermentation yield and titer values at 3 alternative productivity values. Median values of simulated recoveries are also shown. Biomanufacturing facility indices correspond to the configurations listed in **Supplementary Data 1**.

Supplementary Data 3. List of parameters included in uncertainty and sensitivity analyses for the 24 biomanufacturing facilities for which they were performed. Note all chemical prices were converted to the TEA year, 2019 (conversion method detailed in the scripts[1–3]).

Supplementary Data 4. Spearman's rank order correlation coefficients for empirical coefficient k_Y with respect to uncertain parameters common to all 24 biomanufacturing facilities for which sensitivity analyses were performed. "Index" denotes biomanufacturing facility indices (1–24) from **Supplementary Data 1**. Uncertain parameters common to all 24 biomanufacturing facilities for which sensitivity analyses were performed were as follows: (i) fermentation cell mass yield; (ii) feedstock unit price; (iii) feedstock capacity; (iv) biomanufacturing facility operating time; (v) natural gas unit price; (vi) corn steep liquor unit price; (vii) corn steep liquor loading; (viii) electricity unit price; (ix) fermentation inoculum ratio; (x) seed train fermentation yield relative to fermentation yield; (xi) main biomanufacturing facility product storage time; (xii) boiler efficiency; and (xiii) turbogenerator efficiency.

Supplementary Data 5. Spearman's rank order correlation coefficients for empirical coefficient k_0 with respect to uncertain parameters common to all 24 biomanufacturing facilities for which sensitivity analyses were performed. "Index" denotes biomanufacturing facility indices (1–24) from **Supplementary Data 1**. Uncertain parameters common to all 24 biomanufacturing facilities for which sensitivity analyses were performed were as follows: (i) fermentation cell mass yield; (ii) feedstock unit price; (iii) feedstock capacity; (iv) biomanufacturing facility operating time; (v) natural gas unit price; (vi) corn steep liquor unit price; (vii) corn steep liquor loading; (viii) electricity unit price; (ix) fermentation inoculum ratio; (x) seed train fermentation yield relative to fermentation yield; (xi) main biomanufacturing facility product storage time; (xii) boiler efficiency; and (xiii) turbogenerator efficiency.

Supplementary Data 6. Spearman's rank order correlation coefficients for empirical coefficient k_V with respect to uncertain parameters common to all 24 biomanufacturing facilities for which sensitivity analyses were performed. "Index" denotes biomanufacturing facility indices (1–24) from **Supplementary Data 1**. Uncertain parameters common to all 24 biomanufacturing facilities for which sensitivity analyses were performed were as follows: (i) fermentation cell mass yield; (ii) feedstock unit price; (iii) feedstock capacity; (iv) biomanufacturing facility operating time; (v) natural gas unit price; (vi) corn steep liquor unit price; (vii) corn steep liquor loading; (viii) electricity unit price; (ix) fermentation inoculum ratio; (x) seed train fermentation yield relative to fermentation yield; (xi) main biomanufacturing facility product storage time; (xii) boiler efficiency; and (xiii) turbogenerator efficiency.

Supplementary Data 7. Spearman's rank order correlation coefficients for empirical coefficient k_T with respect to uncertain parameters common to all 24 biomanufacturing facilities for which sensitivity analyses

were performed. “Index” denotes biomanufacturing facility indices (1–24) from **Supplementary Data 1**. Uncertain parameters common to all 24 biomanufacturing facilities for which sensitivity analyses were performed were as follows: (i) fermentation cell mass yield; (ii) feedstock unit price; (iii) feedstock capacity; (iv) biomanufacturing facility operating time; (v) natural gas unit price; (vi) corn steep liquor unit price; (vii) corn steep liquor loading; (viii) electricity unit price; (ix) fermentation inoculum ratio; (x) seed train fermentation yield relative to fermentation yield; (xi) main biomanufacturing facility product storage time; (xii) boiler efficiency; and (xiii) turbogenerator efficiency.

References for Description of Additional Supplementary Information

(1) BioSTEAM Development Group. *Triacetic Acid Lactone Biorefineries.* https://github.com/BioSTEAMDevelopmentGroup/Bioindustrial-Park/tree/master/biorefineries/TAL (**2026**).

(2) BioSTEAM Development Group. *Succinic Acid Biorefineries.* https://github.com/BioSTEAMDevelopmentGroup/Bioindustrial-Park/tree/master/biorefineries/succinic (**2026**).

(3) BioSTEAM Development Group. *3-Hydroxypropionic Acid Biorefineries.* https://github.com/BioSTEAMDevelopmentGroup/Bioindustrial-Park/tree/master/biorefineries/HP (**2026**).
